# A scale-free analysis of the HIV-1 genome demonstrates multiple conserved regions of structural and functional importance

**DOI:** 10.1371/journal.pcbi.1007345

**Published:** 2019-09-23

**Authors:** Jordan P. Skittrall, Carin K. Ingemarsdotter, Julia R. Gog, Andrew M. L. Lever

**Affiliations:** 1 Department of Medicine, University of Cambridge, Addenbrooke’s Hospital, Cambridge, United Kingdom; 2 Department of Applied Mathematics and Theoretical Physics, University of Cambridge, Centre for Mathematical Sciences, Cambridge, United Kingdom; 3 Yong Loo Lin School of Medicine, National University of Singapore, Singapore; University of California San Francisco, UNITED STATES

## Abstract

HIV-1 replicates via a low-fidelity polymerase with a high mutation rate; strong conservation of individual nucleotides is highly indicative of the presence of critical structural or functional properties. Identifying such conservation can reveal novel insights into viral behaviour. We analysed 3651 publicly available sequences for the presence of nucleic acid conservation beyond that required by amino acid constraints, using a novel scale-free method that identifies regions of outlying score together with a codon scoring algorithm. Sequences with outlying score were further analysed using an algorithm for producing local RNA folds whilst accounting for alignment properties. 11 different conserved regions were identified, some corresponding to well-known *cis*-acting functions of the HIV-1 genome but also others whose conservation has not previously been noted. We identify rational causes for many of these, including *cis* functions, possible additional reading frame usage, a plausible mechanism by which the central polypurine tract primes second-strand DNA synthesis and a conformational stabilising function of a region at the 5′ end of *env*.

## Author summary

HIV-1 is a very rapidly mutating organism, however some parts of its genetic material change more than others. We looked for coding regions of HIV-1 that change relatively little, by turning the problem of finding such regions into a problem in signal processing, and solving this using a novel analytical approach that we recently described. We investigated why the regions we identified change less, including using the genetic code in the regions we found to prime an algorithm to predict their structures. In some cases there are already known functions for the features we found, in others they provide new insights into the properties of known regions, and in some cases we identify new regions that vary less for as yet unknown functional reasons.

## Introduction

Human Immunodeficiency Virus (HIV) infection remains a significant global health burden, with an estimated 36.7 million people worldwide living with the virus and 1.0 million AIDS-related deaths in 2016 [1]. Despite the success of existing interventions in reducing the annual incidence of HIV, there is still substantial scope for further progress in limiting new infections and optimising diagnosis and treatment of those with HIV. Improving our knowledge of the viral genomic structure and function permits better understanding of the viral lifecycle and host/virus interactions and may suggest interventions to target viral replication and survival.

The HIV-1 genome is one of the most intensively studied genetic sequences. It contains a large number of *cis*-acting regions whose function depends on the structures into which the RNA folds. Many of these have been studied and solved at a secondary structure level and for some there are three-dimensional data. The whole genome has been analysed biochemically at a secondary structure level and many of the known functional regions have been mapped [2]. Much of the genome, however, has been determined to possess conserved structure by such techniques, but with no functional data. Similarly, there are regions where the function of the individual sequence nucleotides, either directly or through coding function, is the main constraint on the sequence and primary structure dominates over secondary structural requirements.

HIV-1 has a high mutation rate, with genome-wide estimates varying from 5.3 × 10^−5^ to 5.8 × 10^−3^ per base per cycle, values of estimates depending upon a number of factors including how reverse transcriptase variability is measured, estimates of recombination rates, and the contribution of cellular apolipoprotein B messenger RNA-editing enzyme-catalytic polypeptide-like 3 (APOBEC3) proteins to variability [3–7]. Notwithstanding variations in the mutation rate from base to base, this number may be sufficiently high to expect every possible point mutation to occur every day in an individual infected with HIV-1 [8]. Conservation within observed sequences is therefore significant and strongly indicative of evolutionary selection pressure, and points towards the presence of structures or functional regions necessary for viral survival. Detection of such conservation can help direct experimental searches to reveal novel structural and/or functional aspects. Without *a priori* knowledge of the dimensions of individual structural or functional elements, analytical techniques need to be able to detect conserved elements regardless of scale.

To investigate further the possible structural form of the viral RNA and to seek evidence of conservation within the genome that might reveal previously unidentified *cis*-acting regions and functions, we performed an intensive bioinformatic analysis of HIV-1 genetic data. We analysed sequence variation, controlling for codon usage, using a novel scale-free method we have developed for finding regions of conservation when the underlying driver of conservation and hence the order of magnitude of the size of conserved regions is not known. This method differs from previous analyses in that it makes no assumptions about the scale of features of interest. As such it will reveal regions that are missed by previous methodologies. Our analysis reveals eleven regions of sequence conservation. Some of these coincide with clusters of established important *cis*-acting functions such as splice sites and their regulatory regions but for others there is no existing explanation; however, some highly likely causes can be attributed to a number of these. Analysing the secondary structure of these sequences reveals some known, but also a number of novel highly conserved structures including a previously undescribed highly stable structure around the central polypurine tract, with clear parallels between this and the structure we identify for the 3′ polypurine tract. Sequence preserved regions such as these represent promising targets for design of HIV therapeutics.

## Materials and methods

### Sequences

For each gene in the HIV-1 genome, as well as for the entire genome, non-recombinant B-subtype sequence data collated between 2009 and 2015 were downloaded from the Los Alamos National Laboratory Filtered Web Alignments database [9]. This database ensures only one sequence is included per patient, that a single representative is included of very similar sequences, and that sequences unlikely to represent natural viable viruses are excluded. Sequence data were further filtered to exclude sequences with uncertain or missing nucleotides in the region being studied, and to exclude sequences whose length indicated that they were incomplete or contained large insertions. A full listing of the sequences used can be found in the supporting information (S1–S10 Tables).

### nMPD analysis

Sequences for each gene were aligned at amino acid level using MUSCLE [10]. Each gene was analysed at codon level with Mathematica [11] using the normalised mean pairwise distance (nMPD) method, as previously described [12], subject to the modification that positions with invariant tryptophan and methionine codons were recorded as uninformative rather than having nMPD set to 1. The nMPD method scores each codon locus by summing the pairwise Hamming distances between all codons. It then normalises to take into account amino acid usage and codon bias, by dividing by the expected distance sum taking into account the amino acids used at that locus and the distribution of codons encoding those amino acids across the genome. At each codon locus a non-negative nMPD score is produced, with low scores representing unexpected conservation of nucleotides after amino acid conservation and codon bias have been taken into account. Such unexpected conservation may represent important structural elements or important functional elements (or both).

The analysis of HIV alignments using the nMPD method raised an additional issue that the gap percentage is higher than that found in alignments of pathogens previously studied using this method, meaning that the way in which gaps are handled can have a substantial effect upon nMPD scores and consequently on the presence or absence of runs of low scores. Gaps were handled by defining the Hamming distance between a gap and any other nucleotide (including a gap) to be zero, but by labelling as uninformative codons where the gap percentage level exceeded 10%. This choice ensures that a minority variant in which a small motif is absent from or present in an otherwise highly conserved region does not result in the entire region being excluded from recognition.

The original nMPD analysis [12] used sliding windows to identify runs of unexpectedly low scores, corresponding to regions of amino acid conservation. Here we instead used the novel scale-free analysis method we recently described [13]. This method is more versatile in that it more easily allows agnostic treatment of uninformative regions and it makes no *a priori* assumptions about the length of conserved motifs, although it still requires conserved regions to be relatively contiguous. The method converts nMPD data to have the appearance of a series of random walks, where at each stage of analysis the presence of an unusually steep descent corresponds to a region of unexpected nucleotide conservation.

Regions determined by the above algorithm to be significantly conserved were aligned to the HXB2 ([14], GenBank accession K03455) and NL4-3 ([15], GenBank accession AF324493) sequences for reporting and analysis.

### Computational folding and structural representation

Regions determined by the algorithm to be significantly conserved were further analysed by taking the relevant section of the whole-genome alignment and folding the region using RNAalifold [16, 17], with default parameters except for using ribosum scoring and disallowing helices of length 1 (lonely pairs). RNAalifold combines phylogenetic information from an input sequence alignment with thermodynamic parameter calculation to determine a consensus secondary structure. The output allows local structures to be analysed for conserved features of interest and conserved loci and reciprocal mutations. Structures are presented in their original RNAalifold format and not modified on the basis of other published structural data as both our novel nMPD analysis and RNAalifold are designed to generate data from multiple sequences, whereas structural prediction from biochemical analyses such as selective 2′-hydroxyl acylation analyzed by primer extension (SHAPE) use only a single sequence as input. Generating a hybrid approach to constrain secondary structure using primary structure data from multiple sequences and e.g. SHAPE data from a single separate sequence would be of uncertain validity.

### Validation

As an additional validation step, we acquired sequences and undertook the nMPD analysis step for A-subtype sequence data (including sequences labelled with sub-subtypes within the A-subtype), in the same way as described for the B-subtype sequence data, *mutatis mutandis*. A full listing of the sequences used can be found in the supporting information (S11–S20 Tables). The purpose of this step was to provide additional validation of the main B-subtype nMPD analysis results and we did not perform further structural analysis of the A-subtype data.

## Results

### nMPD analysis and structural representation

**Fig 1:**
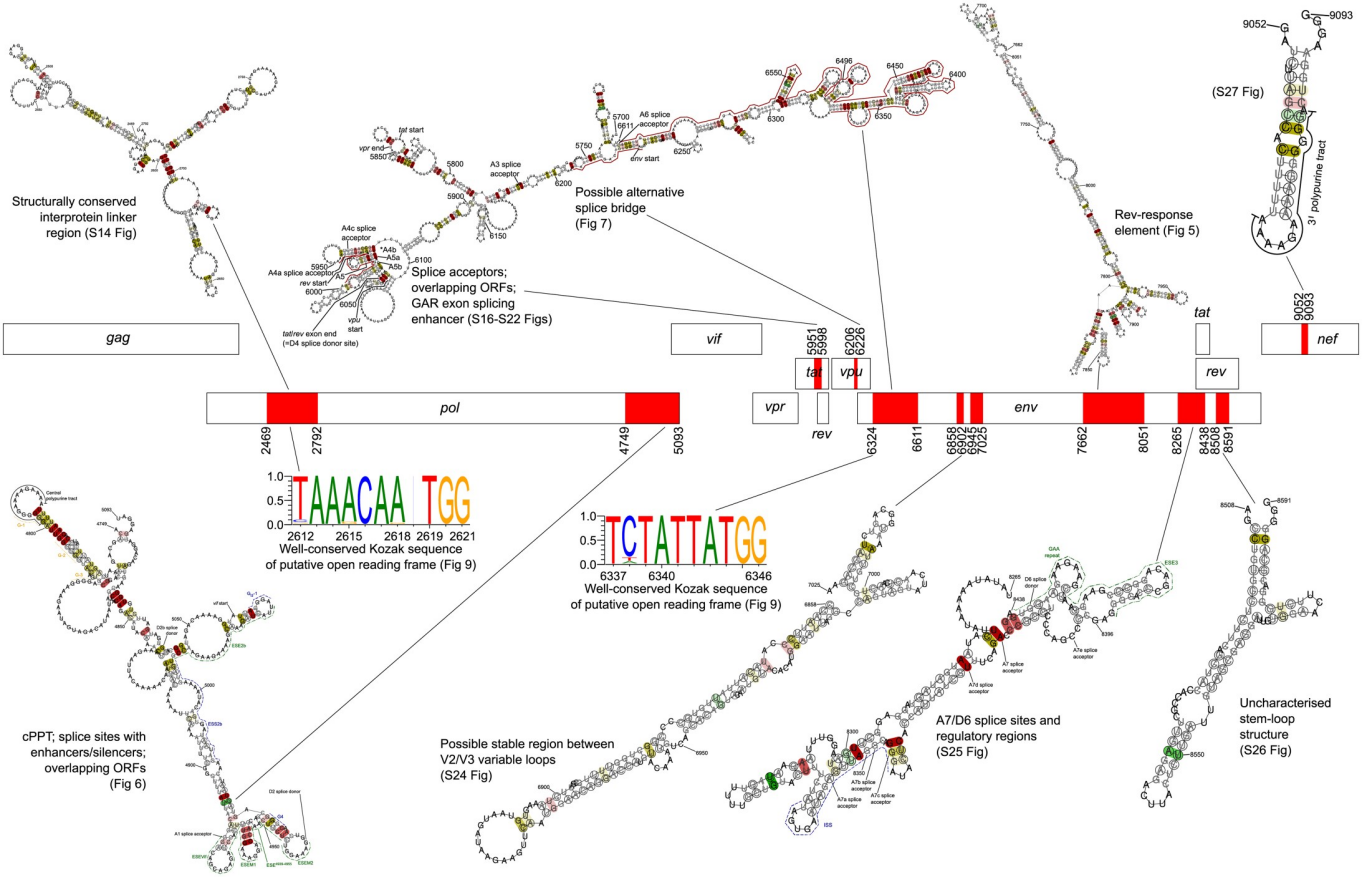
Coding regions of the HIV-1 genome. Regions shown to be conserved by our analysis are highlighted in red. Features of interest are plotted around the schematic representation of the coding regions, with short textual descriptions. Greater detail is given in the text and other figures as indicated in the schematic representation. HXB2 cDNA nucleotide references of significantly conserved regions are given. cPPT = central polypurine tract, ORF = open reading frame.

**Fig 2:**
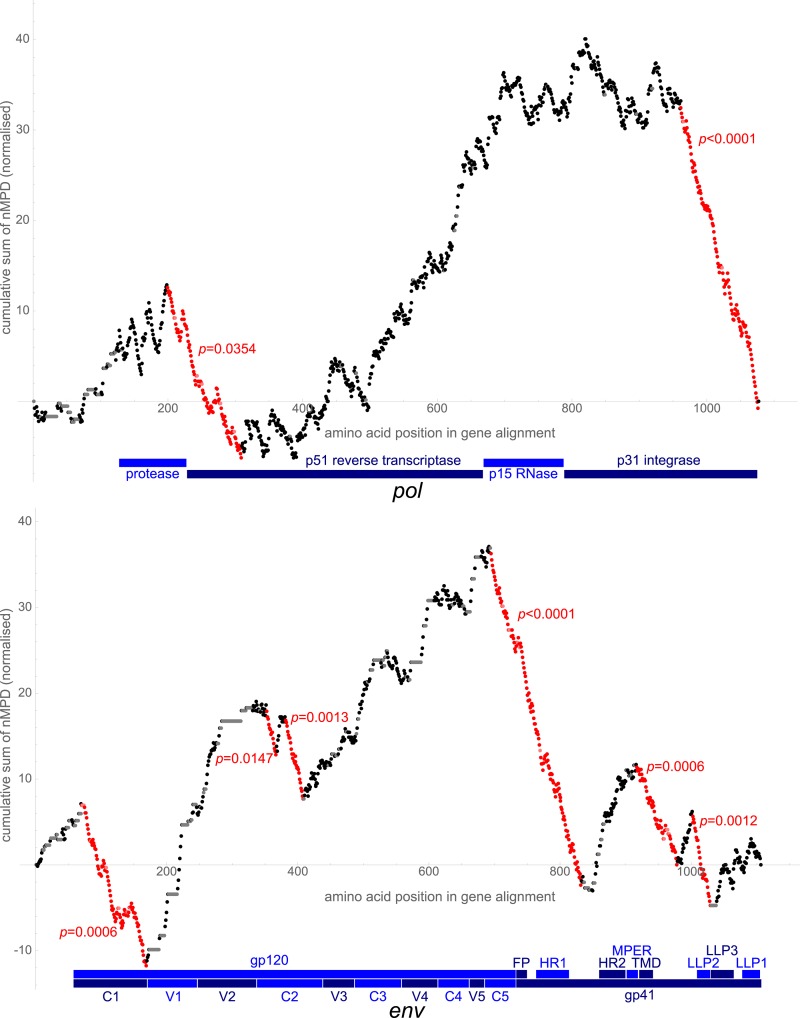
Cumulative sums of nMPDs. Sums are normalised to mean 0 and standard deviation 1. In each case the normalisation used is that of the first analysis, after uninformative points are discounted but before any significant region is discounted. Each locus in the gene is plotted, with the reference along the *x*-axis referring to the codon position in the custom alignment. Descents with a bootstrap *p*-value < 0.05 are highlighted in red, annotated with the *p*-value obtained (this represents the proportion of more extreme bootstraps of the sequence once the regions with lower *p*-values have been excised). Uninformative points are paler (grey or pink). The plots are annotated beneath with blue bars corresponding to the extent of the labelled regions within the genes [9]. Top: *pol* gene. HXB2 nucleotide references of significant regions are 2469–2792 and 4749–5093. Landmarks of the gene are marked in blue beneath the plot. Bottom: *env* gene. HXB2 nucleotide references of significant regions are 6324–6611, 6858–6902, 6945–7025, 7662–8051, 8265–8438, and 8508–8591. Landmarks of the gene are marked in blue beneath the plot: glycoproteins, constant (C) and variable (V) domains [9], fusion peptide (FP) [18], heptad repeats (HR) [19], membrane proximal external region (MPER), transmembrane domain (TMD), lentivirus lytic peptides (LLP) [20].

**Fig 3:**
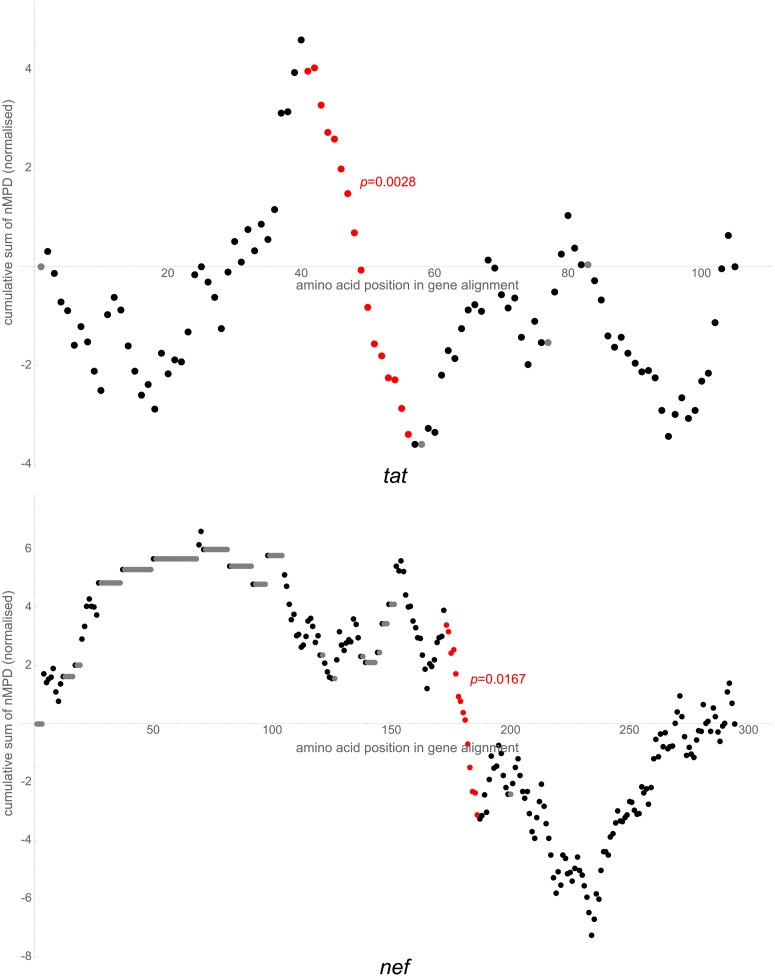
Cumulative sums of nMPDs. Sums are normalised to mean 0 and standard deviation 1, shown in similar fashion to that of Fig 2. Top: *tat* gene. HXB2 nucleotide reference of significant region is 5951–5998. Bottom: *nef* gene. HXB2 nucleotide reference of significant region is 9052–9093.

**Fig 4:**
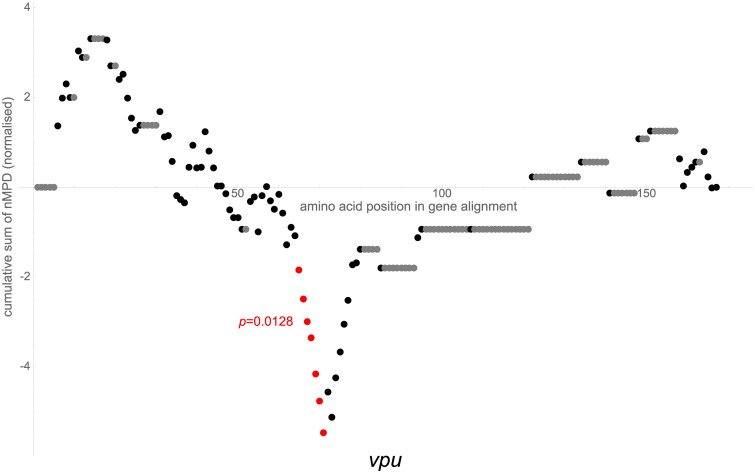
Cumulative sums of nMPDs. Sums are normalised to mean 0 and standard deviation 1, shown in similar fashion to that of Fig 2. *vpu* gene. HXB2 nucleotide reference of significant region is 6206–6226.

nMPD analysis was performed for each gene, using between 309 and 1236 sequences (in total 3651 distinct sequences were used). The output of nMPD analysis is shown in S1–S9 Figs. Five genes (*pol*, *tat*, *vpu*, *env*, *nef*) contained regions that were deemed significantly conserved (Fig 1 and Table 1 for summaries of regions and features of interest found; Figs 2–4 for plots of cumulative analysis for genes in which significantly conserved regions were found; plots of cumulative analysis for remaining genes are in S10–S13 Figs).

**Table 1:** Summary of regions of significant conservation found. See main text for further discussion.

Gene	Order found within gene	HXB2 location	NL4-3 location	*Z*	*p*	Comment
*pol*	2	2469–2792	2469–2792	3.49	0.0354	Interprotein linker region [2] with multiple helices
	1	4749–5093	4749–5093	5.00	< 0.0001	cPPT; splice sites; overlapping ORFs
*tat*	1	5951–5998	5950–5997	3.29	0.0028	Splice acceptors; exon splicing enhancer; overlapping ORFs
*vpu*	1	6206–6226	6202–6222	3.12	0.0133	Possible alternative splice bridge (see main text)
*env*	2	6324–6611	6320–6607	4.41	0.0006	See main text
	6	6858–6902	6848–6892	3.89	0.0147	Stable region between V2 and V3 loops
	4	6945–7025	6935–7015	4.28	0.0013	Stable region between V2 and V3 loops
	1	7662–8051	7652–8041	5.97	< 0.0001	Rev-response element
	5	8265–8438	8255–8428	4.57	0.0006	Splice sites; overlapping ORFs
	3	8508–8591	8498–8581	4.49	0.0012	Overlapping ORFs; see main text
*nef*	1	9052–9093	9042–9083	3.46	0.0168	3′PPT

*Z* values and bootstrap *p*-values are calculated as described in reference [13], with 10000 simulations to generate each bootstrap *p*-value

### The Rev-response element

**Fig 5:**
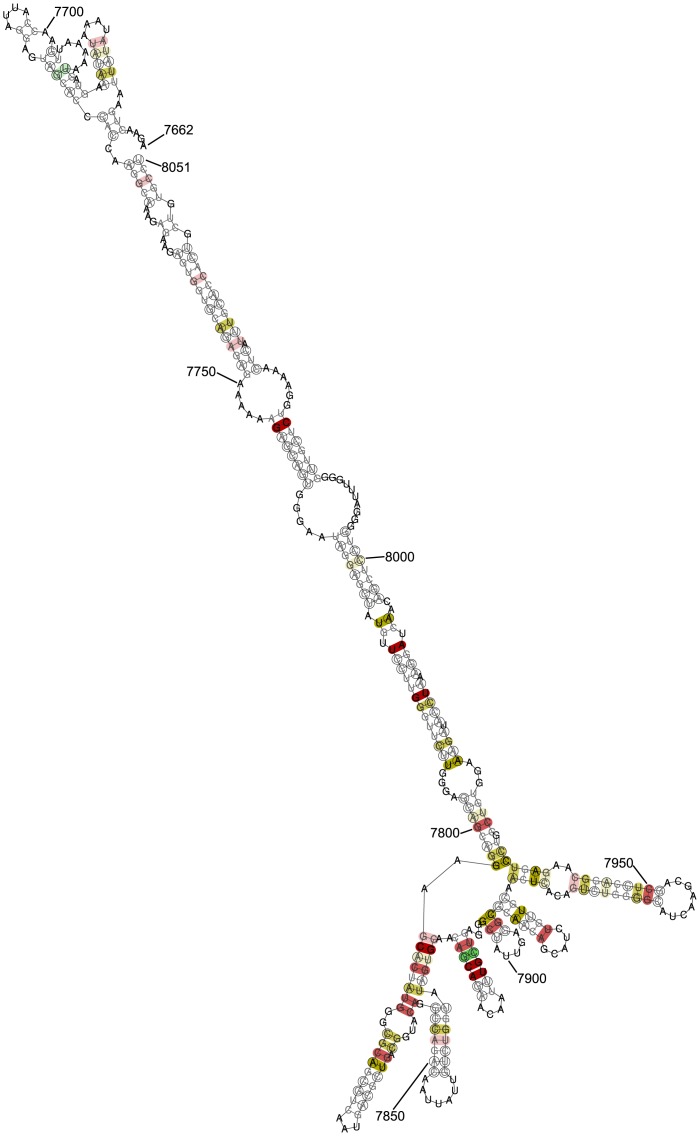
RNAalifold output for local folding of alignment region of interest corresponding to HXB2 reference 7662–8051. This region overlaps substantially with the Rev-response element. The algorithm represents a pair in black if all sequences can form it, and in grey where there are sequences that cannot form the pair. Bases that undergo consistent or compensatory mutation are encircled in black. The number of types of pairs is indicated by shading (red = 1 type, yellow = 2 types, green = 3 types), with the shading paler if there are one or two incompatible pairs, and absent if there are three or more incompatible pairs. Loci where the alignment consensus is a gap are not displayed. Annotations of HXB2 nucleotide references are added manually. The sequences with GenBank accession numbers JQ403098 and KJ948656 were not used to produce this fold: in the case of JQ403098, most of the sequence in the region is missing; in the case of KJ948656, the sequence aligns poorly with the remaining 559 sequences, and including it obscures the annotated pairing of the remaining sequences, although inclusion makes minimal difference to the fold itself.

The conserved region found first in *env*, and the fourth region identified reading from its 5′ end—HXB2 nucleotide reference 7662–8051 (NL4-3 7652–8041)—corresponds well with the well-known highly conserved Rev-response element (RRE) [21–23]. Our method clearly picks up how strongly conserved this region is and delineates its location (see Table 1 for a summary of the statistics, and Fig 2 for a visual representation). Successful detection of this region serves as a positive control for our method. A local fold of this region is shown in Fig 5. The RNAalifold representation is very similar, albeit not identical, to other predictions for folding of the RRE generated by other methods (e.g. reference [2]).

### The central polypurine tract

The conserved region found by the first iteration of our algorithm in *pol*, and the second region identified reading from its 5′ end (Fig 2)—HXB2 nucleotide reference 4749–5093 (NL4-3 4749–5093)—contains the central polypurine tract and associated G-rich sequences suggested to be important for RNA dimer stabilisation via G-quartet formation [24].

**Fig 6:**
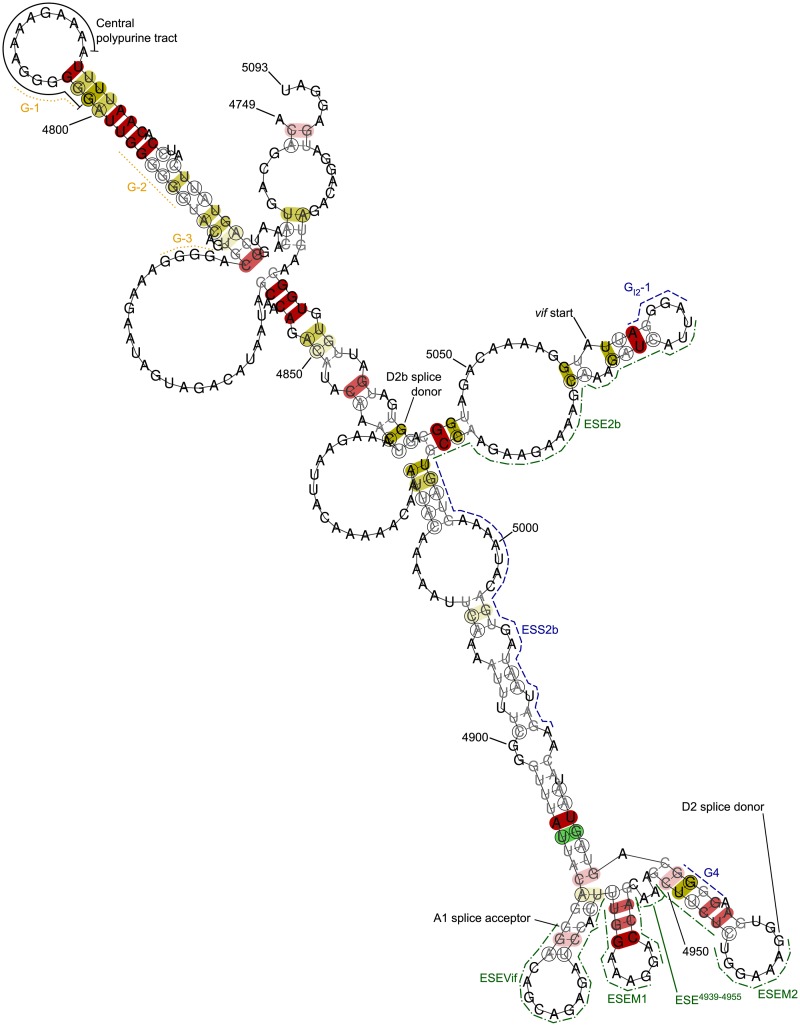
RNAalifold output for local folding of alignment region of interest corresponding to HXB2 nucleotide reference 4749–5093. Previously identified splicing regulators are identified with green dot-dash (for enhancers) and blue dash (for silencers) lines. G-rich sequences suggested to be important for RNA dimer stabilisation via G-quartet formation are highlighted with orange dot lines. A key to the remainder of the annotation is given in the caption for Fig 5.

The region also contains a set of structured stem-loops that contain the A1 splice acceptor site and D2 splice donor site, plus associated exon splicing enhancer elements: ESEVif [25], ESEM [26], and a novel exon splicing enhancer surrounded by the ESEM1 and ESEM2 domains, which was initially identified with the HEXplorer algorithm [27]. The region also contains a splice silencing element, G4 [25], which in our fold of the region is located in the stem of the structure containing the D2 splice site (Fig 6), and a postulated regulatory G run, G_I2_-1 [28], located before the *vif* start site. There is an enhancer motif present between the G4 element and the silencing regulatory G run, termed ESE2b, and directly upstream of this the recently described ESS2b domain; mutations in or changes in accessibility of these domains have been shown to change the proportions of spliced products and affect viral infectivity [29–32]. The region finishes just 3′ of the rarely used D2b splice donor site. In the region is a motif previously identified as evolutionarily conserved, termed stem-loop containing splice acceptor 1 (SLSA1) [33, 34], and although we note that the computational structural prediction by the method we have used does not reproduce the stem-loop of the original prediction, this does not detract from the presence of the conserved nucleic acid motif. The region also contains the start of *vif* (i.e. there are overlapping open reading frames, resulting in conservation of the wobble position nucleotides), and the region ends just 5′ of the *pol* stop codon. A local fold of the region is shown in Fig 6.

### A conserved region in *vpu* may variably promote translation of spliced transcripts, and may stabilise spliced mRNA so as to prevent dimerisation

One region of interest is highlighted in *vpu* (Fig 4), HXB2 nucleotide reference 6206–6226 (NL4-3 6202–6222), just 5′ to the start of *env*. The first region of interest highlighted in *env* (Fig 2) is nearby, at HXB2 nucleotide reference 6324–6611 (NL4-3 6320–6607). Not far 5′ of these regions is the region of interest highlighted in *tat* (Fig 3), HXB2 nucleotide reference 5951–5998 (NL4-3 5950–5997), containing the A4a, A4b, A5, A5a and A5b splice sites, in which possible conserved stem-loop structures have previously been proposed [35, 36], as well as the start of *rev* (and hence a section with overlapping open reading frames and resultant conserved wobble positions) and a GAR exon splicing enhancer [37, 38].

**Fig 7:**
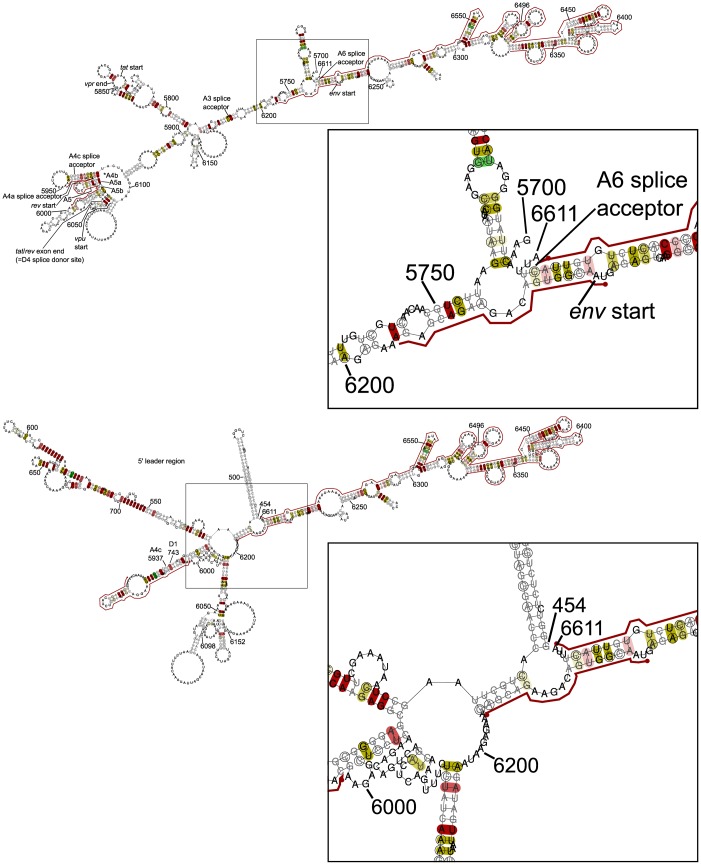
Detail of splice-dependent bridging of upstream elements by the region HXB2 6206–6226. At the top is RNAalifold output for local folding of the unspliced alignment region corresponding to HXB2 reference 5700–6611 (available at larger size in S16 Fig); at the bottom is output for local folding of the alignment region corresponding to a splicing of HXB2 reference 454–743 with 5937–6611 (i.e. D1 donor to A4c acceptor; available at a larger size in S17 Fig). The detail shows how the region HXB2 6206–6226 may variably bridge the unspliced region upstream of HXB2 6206 or the 5′ leader region, in a splicing-dependent manner: see the text for more details. In the spliced RNA, the 5′ leader region adopts the long distance interaction (LDI) conformation. A key to the annotation is given in the caption for Fig 5.

Given the proximity of the regions 6206–6226 and 6324–6611, we folded the entire region 6206–6611 to look for correspondences between elements within the larger region (S15 Fig). This fold gives a correspondence between the regions 6218–6229 and 6597–6608, with a helical structure in a long-range interaction. Stem-loop structures have been postulated 3′ to the *env* signal peptide [2, 39], and we considered whether there may be splicing-dependent folding with the potential to affect translation and/or function. To this end we have produced local folds of alignments corresponding to the three regions of interest with a region directly 5′ to them (S16 Fig), and of alignments corresponding to spliced RNA of the 5′ leader region with the donor site D1 spliced to each of the splice acceptor sites A4c, A4a, A4b, A5, A5a and A5b, and the remainder of the region HXB2 5951–6611 lying 3′ to the respective splice acceptor (S17–S22 Figs; see S22 Table for additional information on the sequences used to produce these figures). These folds indicate that the region HXB2 6206–6226 may have the ability to form stable plausibly functional alternative structures by base pairing intramolecularly in the unspliced RNA or in the chimeric individual spliced species that arise from use of the important A4/5 splice site cluster (Fig 7).

Our folds also suggest that the region HXB2 6324–6611 contains a number of conserved structural motifs.

### Additional regions of interest—Putative open reading frames

Our method highlights a number of further regions of interest, some corresponding to known features of the HIV-1 genome but others whose conservation we cannot fully explain. We detail these below in the sequence in which they appear in the genome, for ease of reference.

#### pol

Two regions of interest are highlighted by our method (Fig 2). The first region, HXB2 nucleotide reference 2469–2792 (NL4-3 2469–2792), is at the protease/reverse transcriptase junction. This region has been previously identified, computationally and by virtue of having low selective 2′-hydroxyl acylation analyzed by primer extension (SHAPE) reactivity, as being structured with high nucleotide conservation, and postulated to act as an interprotein linker region. Parallels between HIV and this region in other primate lentiviruses have been noted [2, 36, 40]. Our method does not pick up the other interprotein linker regions highlighted in the analysis by Watts *et al*. [2]. However, we (as previously) find a high degree of structural conservation for this region (S14 Fig) and it is possible that other regions identified by Watts *et al*. simply do not cross the threshold for identification by our method. The effects of attempts to disrupt the structure of this region have recently been investigated [41]. The structural prediction in this recent investigation differs from ours and is a less extensive stretch of structured nucleotides than we show. Disruption of the major helix predicted in this recent investigation did not lead to a competitive disadvantage in viral replication in competition assays *in vitro*, although this does not necessarily reflect *in vivo* function.

**Fig 8:**
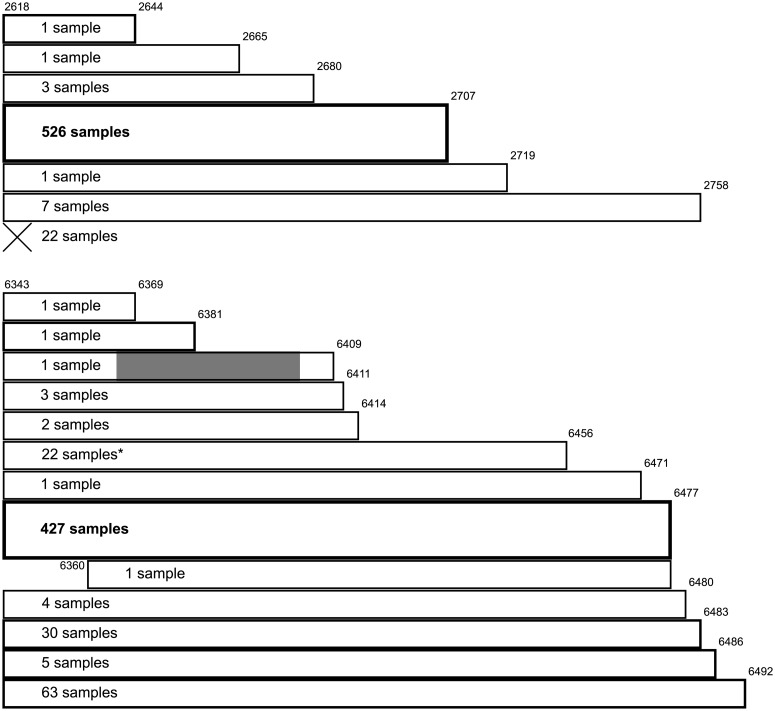
Schematic representations of coding potential of the 561 whole genome sequences in our analysis at putative open reading frames. Widths of the bars correspond to the length of the possible open frame for each set of samples (the line containing ‘X’ corresponds to the set of samples where there is no possible open reading frame), each bar is marked with the number of samples to which the length applies, and HXB2 nucleotide references are marked. The bar corresponding to the putative most common expression of each possible open reading frame is double height with its text in bold. (Top) Representation for the putative open reading frame at HXB2 nucleotide reference 2618–2707. (Bottom) Representation for the putative open reading frame at HXB2 nucleotide reference 6343–6477. The grey region represents a deleted region causing a frameshift. *Indicates that one sequence uses the alternative initiation codon CUG.

**Fig 9:**
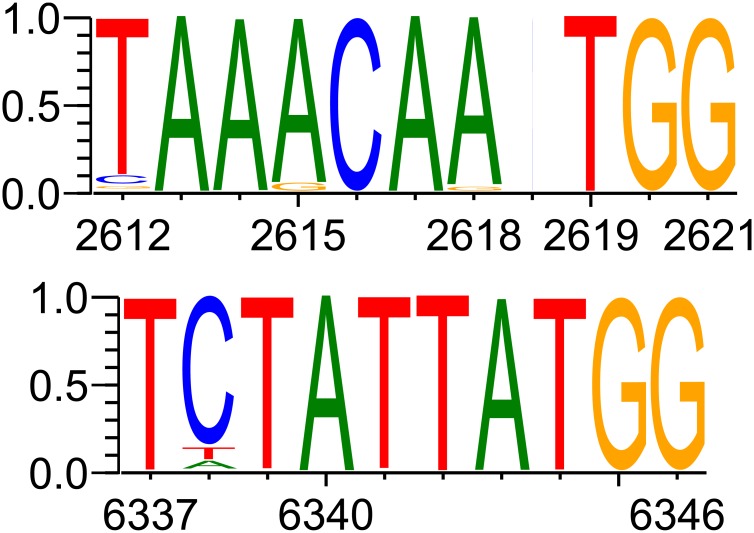
Relative frequencies of nucleotides in the Kozak sequences of the 561 whole genome sequences in our analysis at putative open reading frames. Nucleotide references correspond to HXB2 positions. The almost blank column without a corresponding HXB2 reference represents one whole genome sequence containing an insertion. In each case there is good conservation of a purine in position −3 and guanine in position + 4 (relative to position 1 of the AUG codon). Plots were generated using WebLogo 3 [48, 49].

Sequence analysis reveals a possible, previously unidentified, open reading frame (ORF) at HXB2 nucleotide reference 2618–2707; if such a frame were translated this would explain the constraint on the nucleic acids in the wobble position in the *pol* reading frame. We are not aware of prior empirical evidence for this ORF; we undertook searches for known similar peptides with the translated HXB2 sequence as input using BLASTP 2.8.1+ [42] on the non-redundant proteins database (with HIV and SIV proteins excluded) and using InterProScan 5 with InterPro 72.0 [43, 44]; the first BLASTP hit had an expect value of 5.5 and there were no InterProScan hits. Of the 561 whole genome sequences used in our analysis, 526 (94%) contain initiation and stop codons consistent with this ORF. Of the remaining 35, 22 lack an AUG codon (21 of which instead have GUG, corresponding to a synonymous CAA→CAG mutation in the *pol* open reading frame, and one of which has a CUG codon and a downstream frameshift resulting in a much longer open reading frame than other sequences), 5 have the conserved AUG initiation codon but a premature stop codon in comparison with the consensus, and 8 have the AUG but a later stop codon in comparison with the consensus. Fig 8 gives a schematic representation of the coding potential of the sequences in our analysis relating to this possible ORF. Within the 561 sequences analysed there is consistent conservation of appropriate Kozak consensus nucleotides for the possible open reading frame, with high purine conservation at the −3 position and guanine conservation at the + 4 position relative to position 1 of the AUG codon (Fig 9) consistent with favourable expression of a putative ORF [45–47]. An alternative possibility is the presence of the upstream conserved structure leading to expression of a putative ORF via an internal ribosome entry site (IRES).

The second region, HXB2 nucleotide reference 4749–5093 (NL4-3 4749–5093), is the region containing the central polypurine tract already described above (Fig 6).

#### *tat* and *vpu*

One region of interest is highlighted in each of *tat* and *vpu*; we have considered these already above.

#### env

Six regions of interest are highlighted by our method (Fig 2). The first region, HXB2 nucleotide reference 6324–6611 (NL4-3 6320–6607), is considered above. However, within this sequence there is a further possible alternative ORF at HXB2 nucleotide reference 6343–6477, which could explain the constraint on the nucleic acids in the wobble position in the *env* reading frame, although, again, we are not aware of any pre-existing evidence of this. Again, we undertook searches for known similar peptides with the translated HXB2 sequence as input using BLASTP 2.8.1+ [42] on the non-redundant proteins database (with HIV and SIV proteins excluded) and using InterProScan 5 with InterPro 72.0 [43, 44]; the first BLASTP hit was against a hypothetical *Klebsiella pneumoniae* protein with an expect value of 1 × 10^−18^ and 72% identity (other hits had much higher expect values or lower identity) and there were no InterProScan hits. For this putative ORF, 552 of 561 sequences that we studied (98%) contain initiation and stop codons consistent with an open reading frame at HXB2 nucleotide reference 6343–6474 or with a stop codon within seven codons of this reference. (Of the remaining nine sequences, one has a late initiation codon, and eight have premature stop codons, of which one has a large nucleotide deletion that causes a frameshift.) Fig 8 gives a schematic representation of the coding potential with regard to this possible open reading frame and Fig 9 shows the relative frequencies of nucleotides in the Kozak sequences for this ORF. Similar to the 2612–2621 (HXB2) sequence, there is purine conservation at the −3 position and guanine conservation at the + 4 position, consistent with a favourable Kozak consensus sequence. Here too an upstream conserved structure might represent a possible IRES.

The second region, HXB2 nucleotide reference 6858–6902 (NL4-3 6848–6892), and the third region, HXB2 nucleotide reference 6945–7025 (NL4-3 6935–7015), are located between the V2 and V3 variable loops. Within the second region, Sükösd *et al*. [50] postulate a number of long-range interactions (though our method does not identify the postulated complementary regions). The third region when folded locally gives a possible clover-leaf structure (S23 Fig). The proximity of the two regions, however, motivates a local fold of the entire region 6858–7025 (S24 Fig), which yields a possible stable helix in the region between the V2 and V3 loops.

The fourth region corresponds well with the Rev-response element, and is discussed above.

The fifth region, HXB2 nucleotide reference 8265–8438 (NL4-3 8255–8428), contains the A7a, A7b, A7c, A7d, A7 and A7e splice acceptor and D6 splice donor sites [51, 52], as well as the majority of an exon splicing enhancer, ESE3 [53] (within which is a GAA repeat with independent enhancer activity [54]), and an intron splicing silencer, ISS [55]. The splice acceptor sites mark the beginning of the second *tat* and *rev* exons, so that for part of the region there is an overlap of reading frames (and so conserved wobble positions). The region lies within a region identified as containing a *cis*-enhancing sequence [56]. A local fold of the region, showing a number of stem-loop motifs, is shown in S25 Fig. Regions overlappping this region have previously been identified as showing conservation and local structures proposed [36, 57]; whilst our proposed structure is not identical to either of these, there are multiple similarities.

The sixth region, HXB2 nucleotide reference 8508–8591 (NL4-3 8498–8581), can be seen by eye to contain a clear run of conserved nucleotides (Fig 2) and is clearly identified by our algorithm as possessing unexpected nucleotide conservation (with a bootstrap *p*-value of 0.0006). There is overlap between the *env* and *rev* open reading frames throughout this region (so that wobble positions are expected to be conserved by amino acid constraints); however, nearby regions with similar overlap are not identified as possessing unexpected nucleotide conservation. The local folding of our alignment (S26 Fig) shows a stem-loop structure with a high number of consistent and compensatory mutations. We have not identified in the literature any further explanations for conservation within this region, although it has been postulated previously to form part of a larger structured area [36, 50].

#### nef

One region of interest, HXB2 nucleotide reference 9052–9093 (NL4-3 9042–9083), is highlighted (Fig 3) and contains the 3′ polypurine tract, which is critical for initiation of plus-sense viral DNA replication [58]. The local folding of our alignment (S27 Fig) shows a stem-loop structure with a high number of consistent mutations. There are striking similarities between this and the central polypurine tract structure we show in Fig 6. We note that alternative larger models in which the polypurine tract remains unpaired have been postulated [2, 36, 50]. Mutations in this region have been shown to confer resistance *in vitro* to integrase inhibitors [59]; the structure we propose could undergo a minor conformational change with the previously studied mutations, which could be sufficient to disrupt the integrase target via one of a number of postulated mechanisms [60, 61].

### Validation

The output of nMPD analysis of A-subtype sequences is shown in S28 and S29 Figs. S21 Table summarizes regions of interest found. The validation analysis reproduces all of the regions found in the main analysis with the exception of one region of *pol*, which can be seen in the plot of cumulative analysis in S29 Fig to be conserved, but is not deemed significant by the algorithm. Conversely, the validation analysis finds two regions in *gag* that it deems to be significantly conserved, which can be seen to be conserved in the main B-subtype analysis (S10 Fig), but are not deemed significant in the main analysis.

We also briefly analysed the coding potential of the possible alternative ORFs we have described in *pol* and *env*. For the possible alternative ORF in *pol*, 95 of the 107 sequences that we studied (89%) contained appropriate initiation codons; for the possible ORF in *env*, 106 of the 107 sequences that we studied (99%) contained appropriate initiation codons; the spread of stop codons was similar to that in the B-subtype sequences. Again, there is consistent conservation of the appropriate Kozak consensus nucleotides for the possible open reading frame, with high purine conservation at the −3 positions and guanine conservation at the + 4 positions relative to position 1 of the AUG codons (S30 Fig).

## Discussion

We have applied to a large collection of collated, previously experimentally acquired HIV-1 genomic data a novel version of an informatic pipeline for detecting conserved genetic regions. This combines a method for detecting nucleotide conservation independently of amino acid constraints with a method for detecting runs of conservation *without* requiring prior knowledge of the scale of any feature of interest.

Our pipeline reassuringly detects features where it is already known that nucleotide conservation is required—for example, the Rev-response element is readily identified, and there is a striking correlation between regions our pipeline identifies and known clusters of splice sites—but it goes on to detect novel features whose role has not been described previously. By combining this pipeline with structural prediction tools capable of accounting for sequence variation in the prediction made, we propose a series of conserved structures, some with similarity to structures already published, but some that are novel and plausible, including a highly stable structure surrounding the central polypurine tract, which parallels a structure surrounding the 3′ polypurine tract. The structure we show (Fig 6) would permit exposure of the central polypurine tract for priming of second strand synthesis [62, 63] when it forms a DNA/RNA hybrid: previous work proposes that an oligo-A structure such as this in the context of an inverted repeat would promote reverse transcriptase pausing or even dissociation from the template [64, 65]. Pausing would allow time for second strand synthesis to start using the central polypurine tract RNA as a template before it is hydrolysed; dissociation may leave the central polypurine tract RNA *in situ* for use as a template. Formation of G tetrads could provide a switch mechanism to an alternative conformation that would disrupt the structure we propose when the RNA is in the monomeric or dimeric form, and might have roles in obscuring double-stranded RNA runs to evade innate immunity or to foster intergenome dimer formation for encapsidation.

In one case (the *tat*/*vpu*/*env* region), the structures we propose, taken in conjunction with the identified regions of nucleotide conservation, immediately lend themselves to plausible functions in alternative splicing. Variable bridging of the HXB2 6206–6226 sequence between the 5′ leader region or the unspliced region upstream of HXB2 6206 might either promote translation of the partially spliced *env* transcripts from the immediately downstream *env* translational initiation codon, or stabilise part of the structure located 5′ of this region that contains the splice donor site D4, which is used in conjunction with downstream splice acceptor sites to generate completely spliced mRNA transcripts. Additionally, the positioning of the conserved regions of interest we have identified within the spliced folds, together with the folds themselves, raises the possibility that splicing from the D1 donor site to the acceptor sites in the A4c to A5b region may result in a conformational stabilisation of the 5′ cap, possibly contributing to the extensive utilisation of the A4/5 splice acceptor cluster for over 90% of *env*, *vpu*, *rev* and *nef* transcripts [66]. The leader region can adopt two alternating structures [67], and stabilisation of the leader region in the long distance interaction (LDI) conformation, rather than the branched multiple hairpins (BMH) conformation [68], may imply a function for this elongated helix loop in the spliced RNA. The LDI conformation is proposed to reduce inter-molecular genome dimerisation and this would be a valuable property to preserve in spliced mRNA to prevent dimerisation using the high-affinity SL1 dimer linkage site, restricting this property to unspliced viral RNA. Although a previous *in vivo* analysis of RNA structure in this region did not identify the LDI conformation [69], detection of this in spliced RNA may have been compromised by use of a primer downstream of the *gag* start site.

The key to generating these structures—and the novel step we have undertaken—is priming the folding algorithm with just the conserved region, identified by our scale-free analysis of nMPDs, that may form a conserved structure, to increase the algorithm’s ability to select and predict the locally conserved secondary structure. To prime in this way cannot be achieved without knowledge of the extent of the conserved region, which is a novel feature of the detection pipeline we have used.

Whilst we have strong mathematical evidence for the existence of conserved regions we rely on prior publications to support the structural predictions we have made, where such evidence exists. The largest and most striking region of conservation we identify is the Rev-response element and this is powerful validation of the predictive accuracy of our modelling. Other regions we show are based on what are highly accurate structural prediction programs but some need functional and structural validation and must be accepted as putative at present.

We have mentioned two regions where one explanation of genetic conservation would be hithertofore undescribed open reading frames. We caution that we are not aware of any empirical evidence of translation of these putative frames. The lack of strong database search hits against peptides of known function indicates that putative products of these frames are not directly analagous to already-characterised peptides. Some regions of the genome where ovelapping reading frames are known to exist are not identified as having a high degree of conservation by our method, indicating that the degree of variability of some regions of HIV-1 without wobble positions is comparable with the background degree of variability.

Our method relies upon what is in effect a signal processing algorithm that most easily picks up relatively long runs of conserved genetic elements in a background where there is less conservation. The algorithm is applied gene-by-gene. This explains why, for example, the algorithm does well at detecting conserved elements within the *env* gene, where the overall high degree of variability makes it easier to identify conserved elements (in signal processing terms, the signal-to-noise ratio is high). It also explains why, for example, the algorithm fails to detect the well-characterised frameshift structure towards the end of *gag*, which has only a relatively short run of highly conserved nucleotides. It is important to understand that this algorithm is optimised for identifying longer contiguous conserved genetic elements such as those where RNA structure needs to be conserved and it may miss short or non-contiguous motifs, especially in genes with relatively lower nucleic acid variability overall. Whilst there is no minimum run length the algorithm can detect (it can pick up any sufficiently strong signal of length at least 2), the degree of conservation of a region versus background variability needs to be much higher for detection of shorter conserved regions. This is illustrated in Fig 5, left panel of our paper describing the algorithm [13], and to aid understanding of the degree of conservation required to detect much shorter regions using this algorithm, we have extended our benchmarking simulation to illustrate results for signal lengths down to 2 (S31 Fig). Our validation step using A-subtype sequences shows consistency of the method’s performance between related datasets, whilst illustrating a feature of our algorithm that minor differences between datasets may lead to minor differences between the start and end positions of regions deemed significantly conserved. The nMPD algorithm is not applied to non-coding regions at all and will not pick up conserved elements in non-coding regions. Thus overall our method cannot detect known microRNAs or long non-coding RNAs [70].

This novel approach to detection of nucleotide conservation in HIV-1 has revealed unexpected insights into the viral genome and can be applied similarly to other RNA viral sequences. The identification of unsuspected regions of conservation is of importance as it directs experimental approaches for investigation into regions of conserved structure or function. In conjunction with further experimental validation, it can suggest loci that are likely to represent stable drug targets.

## Supporting information

S1 Fig Normalised mean pairwise distance (nMPD) against amino acid position in the custom gene alignment for *gag* gene. Points corresponding to codons deemed to fall within regions of significant conservation are in red (pink for uninformative points as defined in main text). (There is no such point for this gene.) Points corresponding to codons outside regions of significant conservation are in black (grey for uninformative points). Uninformative points are plotted at nMPD of 1.
(PDF)

S2 Fig Normalised mean pairwise distance (nMPD) against amino acid position in gene alignment for *pol* gene. See the caption for S1 Fig for a description of the point colours.
(PDF)

S3 Fig Normalised mean pairwise distance (nMPD) against amino acid position in gene alignment for *vif* gene. See the caption for S1 Fig for a description of the point colours.
(PDF)

S4 Fig Normalised mean pairwise distance (nMPD) against amino acid position in gene alignment for *vpr* gene. See the caption for S1 Fig for a description of the point colours.
(PDF)

S5 Fig Normalised mean pairwise distance (nMPD) against amino acid position in gene alignment for *tat* gene. See the caption for S1 Fig for a description of the point colours.
(PDF)

S6 Fig Normalised mean pairwise distance (nMPD) against amino acid position in gene alignment for *rev* gene. See the caption for S1 Fig for a description of the point colours.
(PDF)

S7 Fig Normalised mean pairwise distance (nMPD) against amino acid position in gene alignment for *vpu* gene. See the caption for S1 Fig for a description of the point colours.
(PDF)

S8 Fig Normalised mean pairwise distance (nMPD) against amino acid position in gene alignment for *env* gene. See the caption for S1 Fig for a description of the point colours.
(PDF)

S9 Fig Normalised mean pairwise distance (nMPD) against amino acid position in gene alignment for *nef* gene. See the caption for S1 Fig for a description of the point colours.
(PDF)

S10 Fig Cumulative sums of nMPDs normalised to mean 0 and standard deviation 1 for *gag* gene. Shown in similar fashion to that of Fig 2.
(PDF)

S11 Fig Cumulative sums of nMPDs normalised to mean 0 and standard deviation 1 for *vif* gene. Shown in similar fashion to that of Fig 2.
(PDF)

S12 Fig Cumulative sums of nMPDs normalised to mean 0 and standard deviation 1 for *vpr* gene. Shown in similar fashion to that of Fig 2.
(PDF)

S13 Fig Cumulative sums of nMPDs normalised to mean 0 and standard deviation 1 for *rev* gene. Shown in similar fashion to that of Fig 2.
(PDF)

S14 Fig RNAalifold output for local folding of alignment region of interest corresponding to HXB2 nucleotide reference 2469–2792. A key to the annotation is given in the caption for Fig 5.
(PDF)

S15 Fig RNAalifold output for local folding of alignment region corresponding to HXB2 reference 6206–6611, within which are the regions of interest 6206–6226 and 6324–6611. A key to the annotation is given in the caption for Fig 5.
(PDF)

S16 Fig RNAalifold output for local folding of alignment region corresponding to HXB2 reference 5700–6611, within which are the regions of interest 5951–5998, 6206–6226 and 6324–6611. Subregions of interest are represented by red lines adjacent to the regions’ respective nucleotides. A key to the annotation is given in the caption for Fig 5. A GAR exon splicing enhancer overlapping a region of interest is present in the region 5977–6002, but is not included in the labels for clarity.
(PDF)

S17 Fig RNAalifold output for local folding of alignment region corresponding to a splicing of HXB2 reference 454–743 with 5937–6611 (i.e. D1 donor to A4c acceptor). A key to the annotation is given in the caption for Fig 5. Additional features are labelled in S16 Fig, but are omitted here for clarity.
(PDF)

S18 Fig RNAalifold output for local folding of alignment region corresponding to a splicing of HXB2 reference 454–743 with 5955–6611 (i.e. D1 donor to A4a acceptor). A key to the annotation is given in the caption for Fig 5. Additional features are labelled in S16 Fig, but are omitted here for clarity.
(PDF)

S19 Fig RNAalifold output for local folding of alignment region corresponding to a splicing of HXB2 reference 454–743 with 5961–6611 (i.e. D1 donor to A4b acceptor). A key to the annotation is given in the caption for Fig 5. Additional features are labelled in, but are omitted here for clarity.
(PDF)

S20 Fig RNAalifold output for local folding of alignment region corresponding to a splicing of HXB2 reference 454–743 with 5977–6611 (i.e. D1 donor to A5 acceptor). A key to the annotation is given in the caption for Fig 5. Additional features are labelled in S16 Fig, but are omitted here for clarity.
(PDF)

S21 Fig RNAalifold output for local folding of alignment region corresponding to a splicing of HXB2 reference 454-743 with 5981-6611 (i.e. D1 donor to A5a acceptor). A key to the annotation is given in the caption for Fig 5. Additional features are labelled in S16 Fig, but are omitted here for clarity.
(PDF)

S22 Fig RNAalifold output for local folding of alignment region corresponding to a splicing of HXB2 reference 454–743 with 5984–6611 (i.e. D1 donor to A5b acceptor). A key to the annotation is given in the caption for Fig 5. Additional features are labelled in S16 Fig, but are omitted here for clarity.
(PDF)

S23 Fig RNAalifold output for local folding of alignment regions corresponding to HXB2 references 6858–6902 and 6945–7025. Top: region corresponding to HXB2 reference 6858–6902. Bottom: region corresponding to HXB2 reference 6945–7025. The modified free energies [17, 71] for these predicted structures are −12.99kcal/mol and −33.06kcal/mol, respectively. A key to the annotation is given in the caption for Fig 5.
(PDF)

S24 Fig RNAalifold output for local folding of alignment region corresponding to HXB2 reference 6858–7025, within which are the regions of interest 6858–6902 and 6945–7025. The modified free energy [17, 71] for this larger predicted structure is −75.42kcal/mol, substantially lower than the sum of the modified free energies of the smaller predicted structures (−46.05kcal/mol; this does not take into account the region 6903–6944, which is small in comparison with the overall region size). A key to the annotation is given in the caption for Fig 5.
(PDF)

S25 Fig RNAalifold output for local folding of alignment region of interest corresponding to HXB2 reference 8265–8438. A key to the annotation is given in the captions for Figs 5 and 6. The sequence with GenBank accession number JQ403098 was not used to produce this fold as its sequenced region and the region of interest do not overlap.
(PDF)

S26 Fig RNAalifold output for local folding of alignment region of interest corresponding to HXB2 reference 8508–8591. A key to the annotation is given in the caption for Fig 5. The sequence with GenBank accession number JQ403098 was not used to produce this fold as its sequenced region and the region of interest do not overlap.
(PDF)

S27 Fig RNAalifold output for local folding of alignment region of interest corresponding to HXB2 reference 9052–9093. A key to the annotation is given in the caption for Fig 5. Some sequences were not used when producing this fold: see S23 Table for more information.
(PDF)

S28 Fig Normalised mean pairwise distance (nMPD) against amino acid position in gene alignment for genes in the A-subtype validation analysis. See the caption for S1 Fig for a description of the point colours.
(PDF)

S29 Fig Cumulative sums of nMPDs normalised to mean 0 and standard deviation 1 for genes in the A-subtype validation analysis. Shown in similar fashion to that of Fig 2.
(PDF)

S30 Fig Relative frequencies of nucleotides in the Kozak sequences of the 107 whole genome sequences in the A-subtype validation analysis at putative open reading frames. Shown in similar fashion to that of Fig 9.
(PDF)

S31 Fig Benchmarking simulation output illustrating increased signal strength required for detection of small conserved regions. Replicating Fig 5 of the benchmarking work in [13], but here extending to include shorter signals. This shows that in the range of short signals (less than width 50), the shorter the signal, the stronger the strength of the signal must be for successful detection. Simulated data used for signal widths from 50 to 450 are identical to those used in the plot in [13].
(PDF)

S1 Table GenBank accession numbers of the 899 B-subtype sequences used in the nMPD analysis of the *gag* gene. (PDF)

S2 Table GenBank accession numbers of the 329 B-subtype sequences used in the nMPD analysis of the *pol* gene. (PDF)

S3 Table GenBank accession numbers of the 1194 B-subtype sequences used in the nMPD analysis of the *vif* gene. (PDF)

S4 Table GenBank accession numbers of the 1067 B-subtype sequences used in the nMPD analysis of the *vpr* gene. (PDF)

S5 Table GenBank accession numbers of the 309 B-subtype sequences used in the nMPD analysis of the *tat* gene. (PDF)

S6 Table GenBank accession numbers of the 748 B-subtype sequences used in the nMPD analysis of the *rev* gene. (PDF)

S7 Table GenBank accession numbers of the 1236 B-subtype sequences used in the nMPD analysis of the *vpu* gene. (PDF)

S8 Table GenBank accession numbers of the 551 B-subtype sequences used in the nMPD analysis of the *env* gene. (PDF)

S9 Table GenBank accession numbers of the 951 B-subtype sequences used in the nMPD analysis of the *nef* gene. (PDF)

S10 Table GenBank accession numbers of the 561 B-subtype sequences used where an entire genome alignment was used as the basis of analysis. (PDF)

S11 Table GenBank accession numbers of the 137 A-subtype sequences used in the validation analysis of the *gag* gene. (PDF)

S12 Table GenBank accession numbers of the 71 A-subtype sequences used in the validation analysis of the *pol* gene. (PDF)

S13 Table GenBank accession numbers of the 182 A-subtype sequences used in the validation analysis of the *vif* gene. (PDF)

S14 Table GenBank accession numbers of the 145 A-subtype sequences used in the validation analysis of the *vpr* gene. (PDF)

S15 Table GenBank accession numbers of the 71 A-subtype sequences used in the validation analysis of the *tat* gene. (PDF)

S16 Table GenBank accession numbers of the 158 A-subtype sequences used in the validation analysis of the *rev* gene. (PDF)

S17 Table GenBank accession numbers of the 151 A-subtype sequences used in the validation analysis of the *vpu* gene. (PDF)

S18 Table GenBank accession numbers of the 78 A-subtype sequences used in the validation analysis of the *env* gene. (PDF)

S19 Table GenBank accession numbers of the 185 A-subtype sequences used in the validation analysis of the *nef* gene. (PDF)

S20 Table GenBank accession numbers of the 107 A-subtype sequences used where an entire genome alignment was used for validation. (PDF)

S21 Table Summary of regions of significant conservation found in A-subtype validation exercise. See main text for further discussion.
(PDF)

S22 Table GenBank accession numbers of the sequences used where an entire genome alignment with sequenced 5′ header was used as the basis of analysis. Not all of the sequences whose accession numbers are listed in S10 Table contain a fully sequenced 5′ header; only those listed here are therefore used in the production of S16–S22 Figs.
(PDF)

S23 Table Sequences omitted from the 3′ polypurine tract plot. A number of the “complete genome” sequences in fact have a sequenced region that terminates upstream of the end of the region given by HXB2 nucleotide reference 9052–9093. These sequences have therefore not been used in the production of S27 Fig. The GenBank accession numbers of these sequences are given above. One of these sequences (accession number AF042104) does have sequence data downstream of the end of the region of interest, but there is a large piece of sequence data missing within the region itself, and so the entire sequence is omitted.
(PDF)
