# Supplementary figures and images for: A scale-free analysis of the HIV-1 genome demonstrates multiple conserved regions of structural and functional importance
Source: PLoS Comput Biol. 2019 Sep 23;15(9):e1007345. doi: 10.1371/journal.pcbi.1007345 (PMC6791557; doi:10.1371/journal.pcbi.1007345)

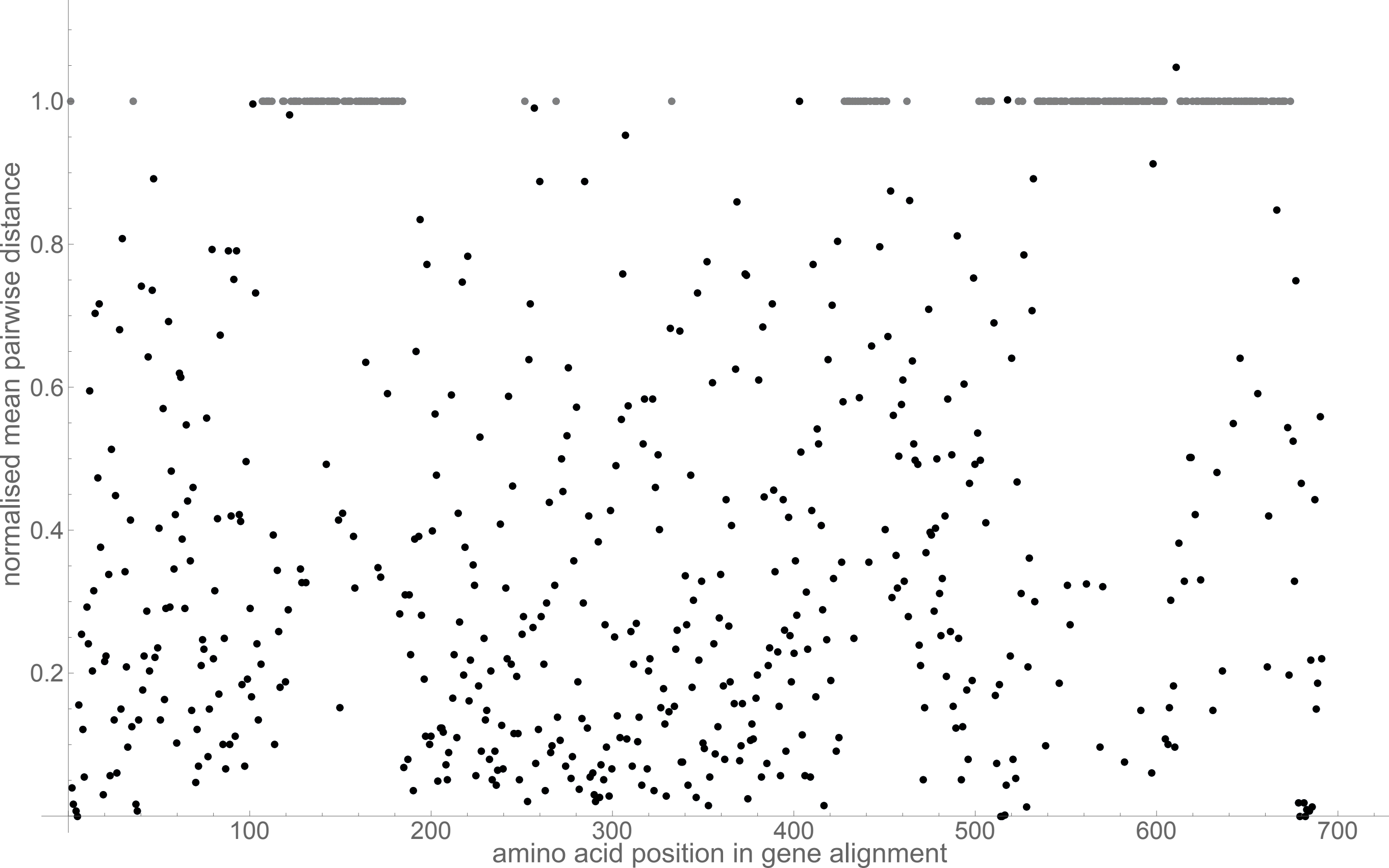

Supplement: S1 Fig — Points corresponding to codons deemed to fall within regions of significant conservation are in red (pink for uninformative points as defined in main text). (There is no such point for this gene.) Points corresponding to codons outside regions of significant conservation are in black (grey for uninformative points). Uninformative points are plotted at nMPD of 1. (PDF) [file pcbi.1007345.s001.pdf]

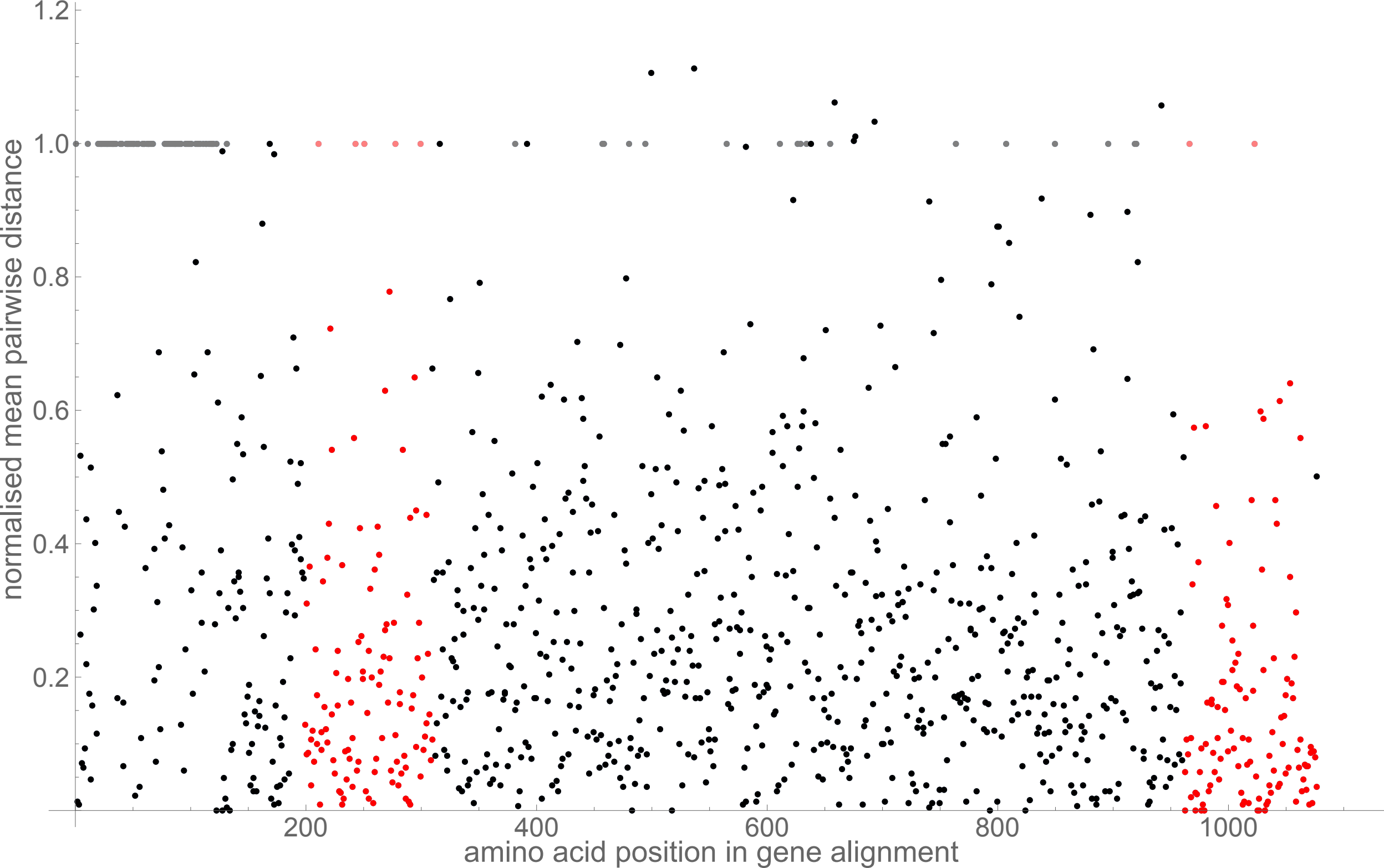

Supplement: S2 Fig — See the caption for S1 Fig for a description of the point colours. (PDF) [file pcbi.1007345.s002.pdf]

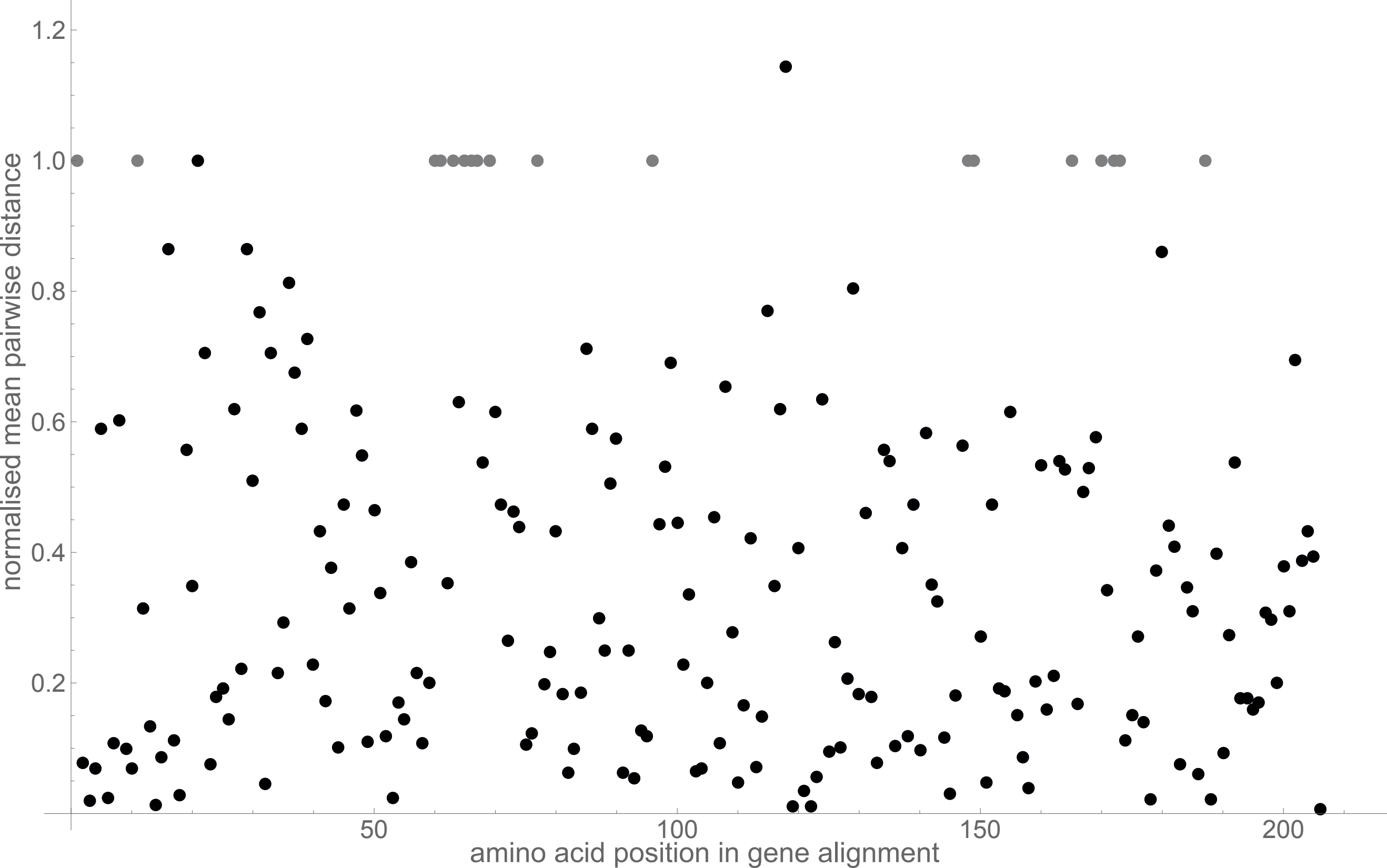

Supplement: S3 Fig — See the caption for S1 Fig for a description of the point colours. (PDF) [file pcbi.1007345.s003.pdf]

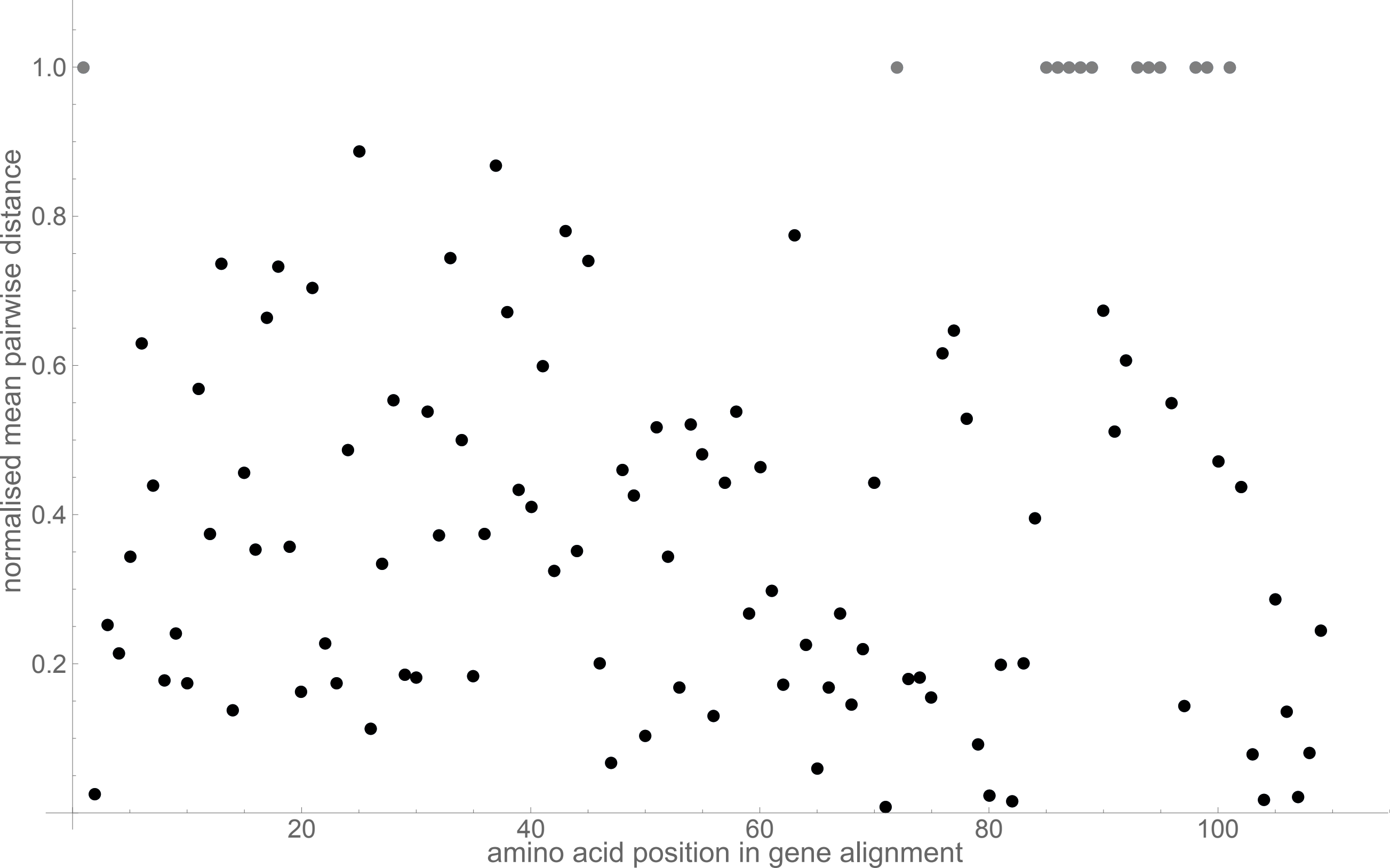

Supplement: S4 Fig — See the caption for S1 Fig for a description of the point colours. (PDF) [file pcbi.1007345.s004.pdf]

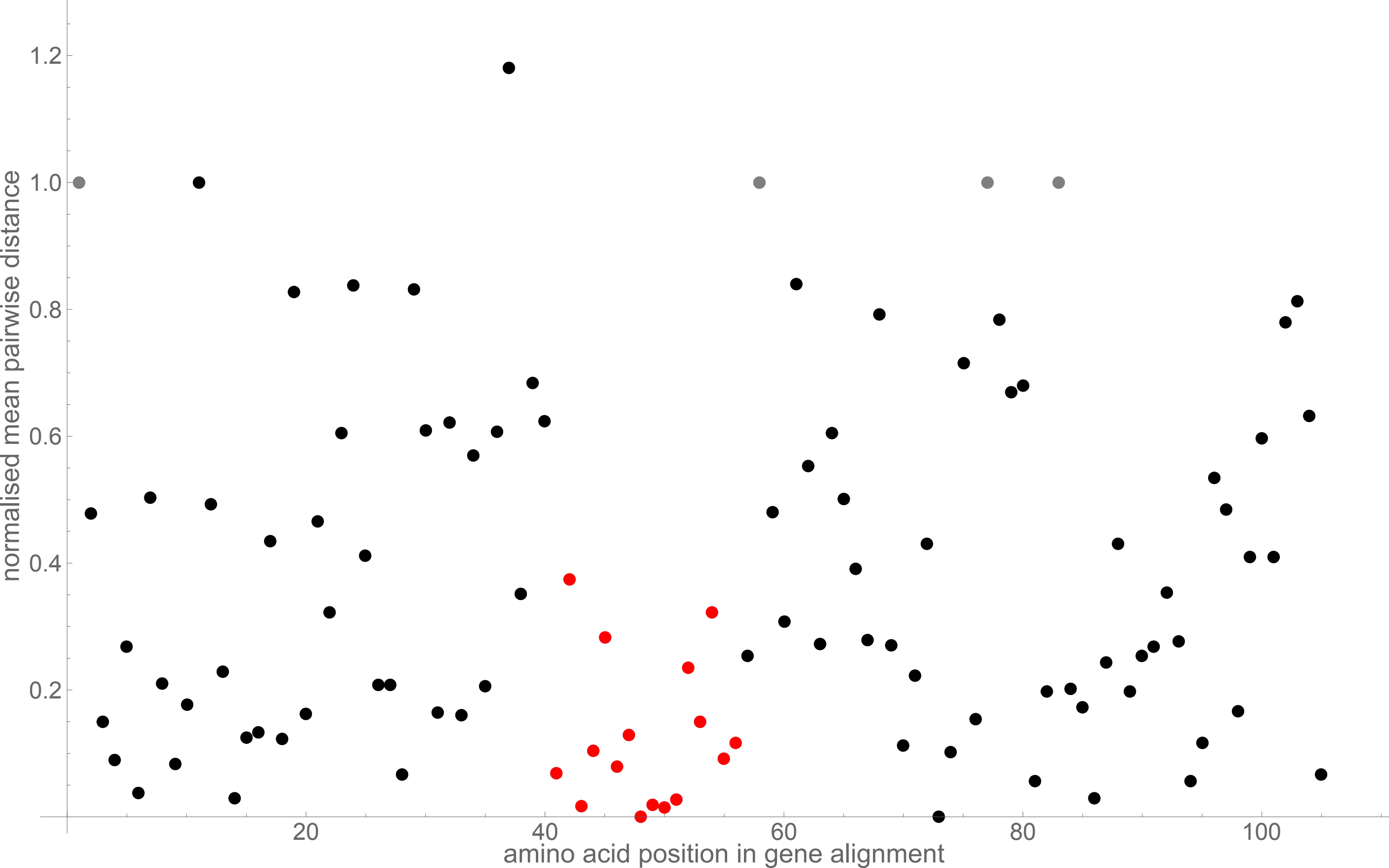

Supplement: S5 Fig — See the caption for S1 Fig for a description of the point colours. (PDF) [file pcbi.1007345.s005.pdf]

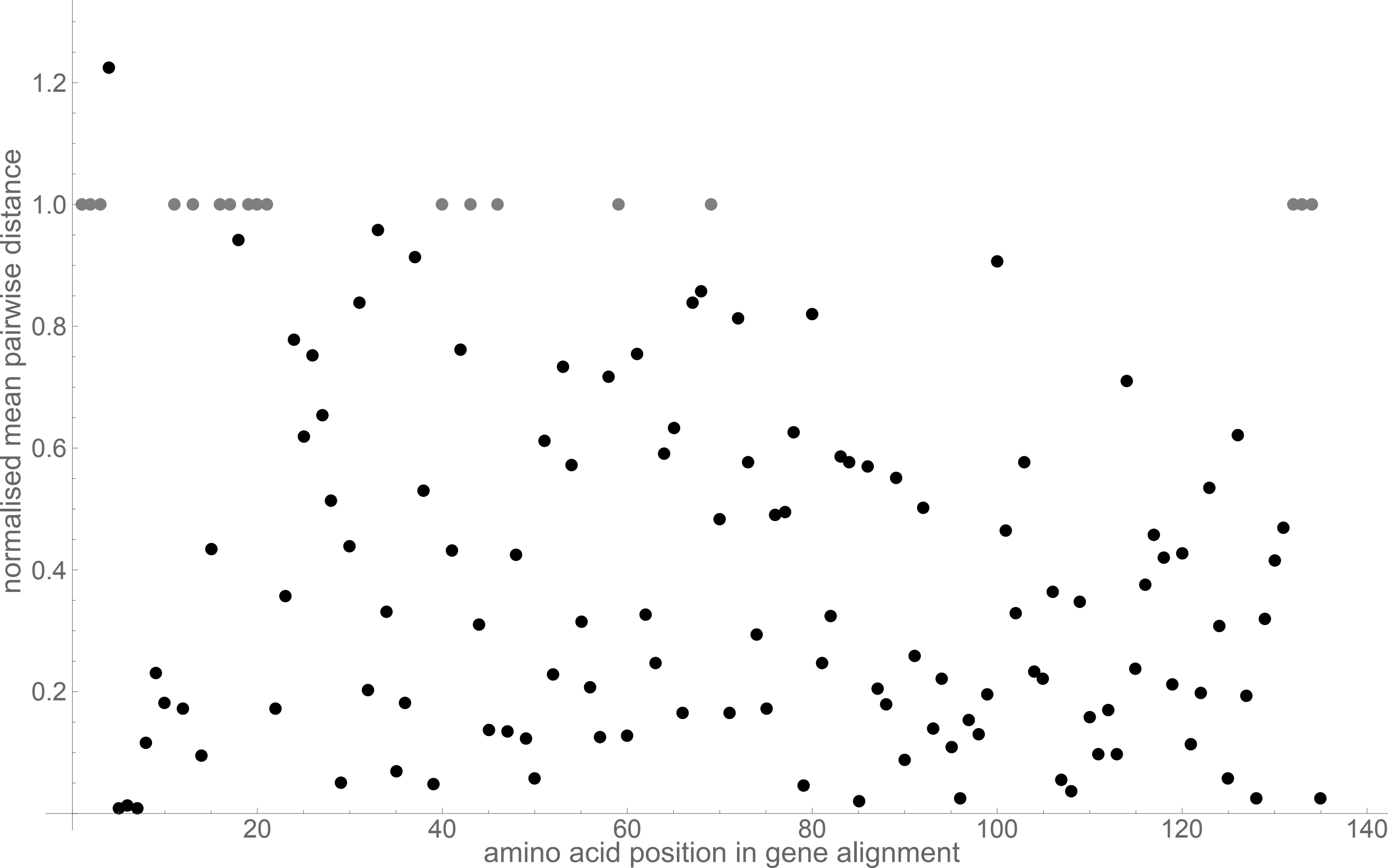

Supplement: S6 Fig — See the caption for S1 Fig for a description of the point colours. (PDF) [file pcbi.1007345.s006.pdf]

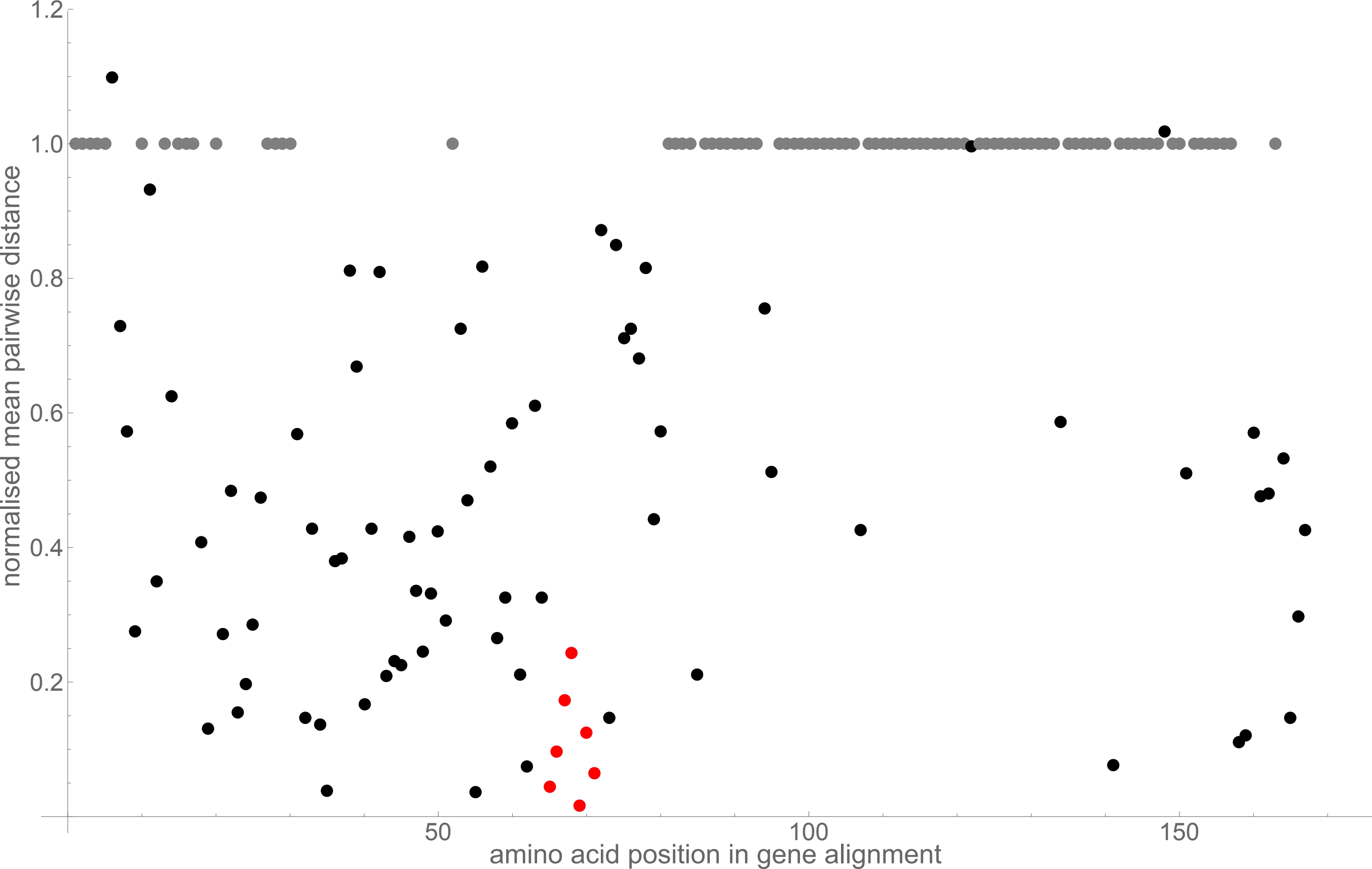

Supplement: S7 Fig — See the caption for S1 Fig for a description of the point colours. (PDF) [file pcbi.1007345.s007.pdf]

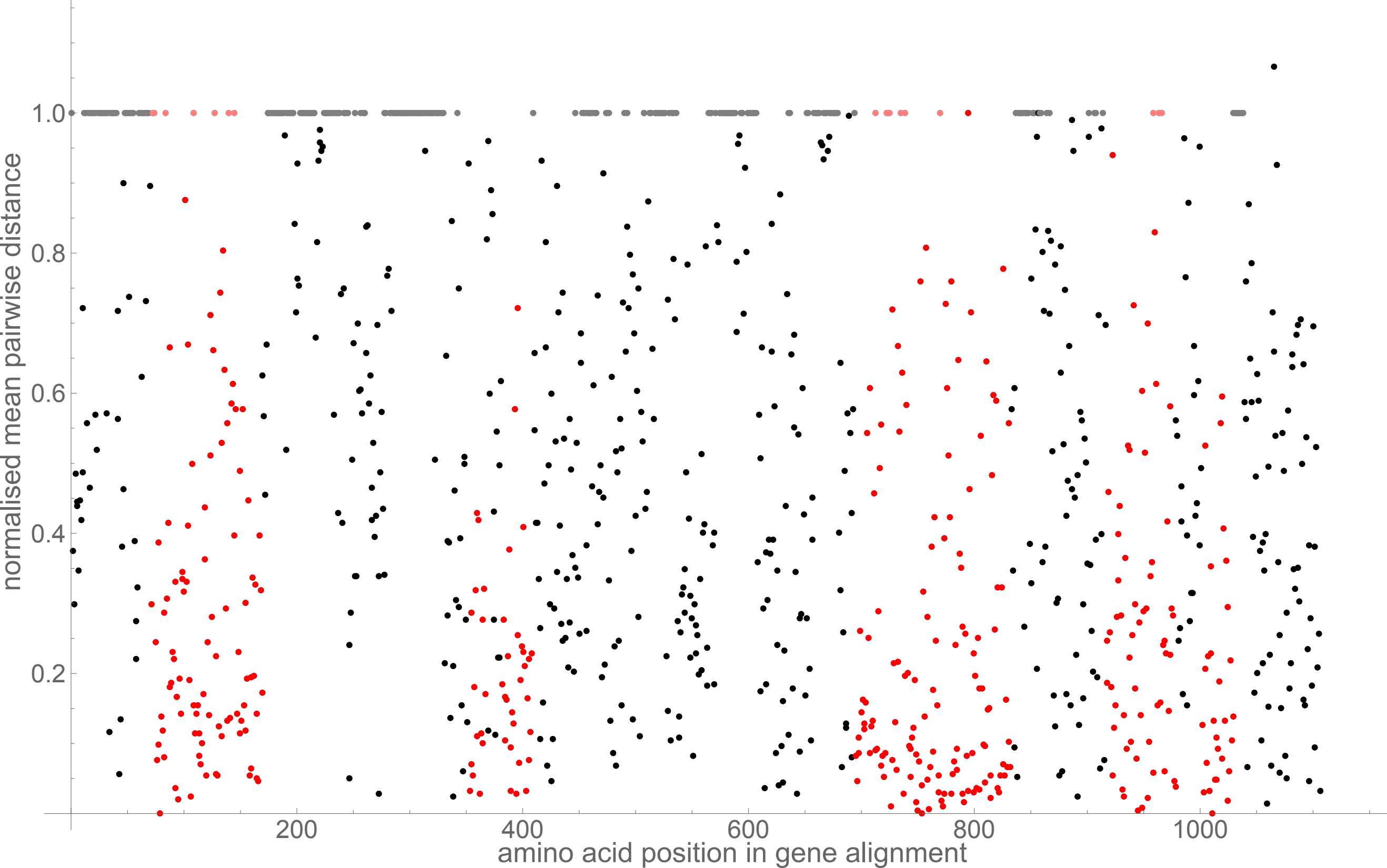

Supplement: S8 Fig — See the caption for S1 Fig for a description of the point colours. (PDF) [file pcbi.1007345.s008.pdf]

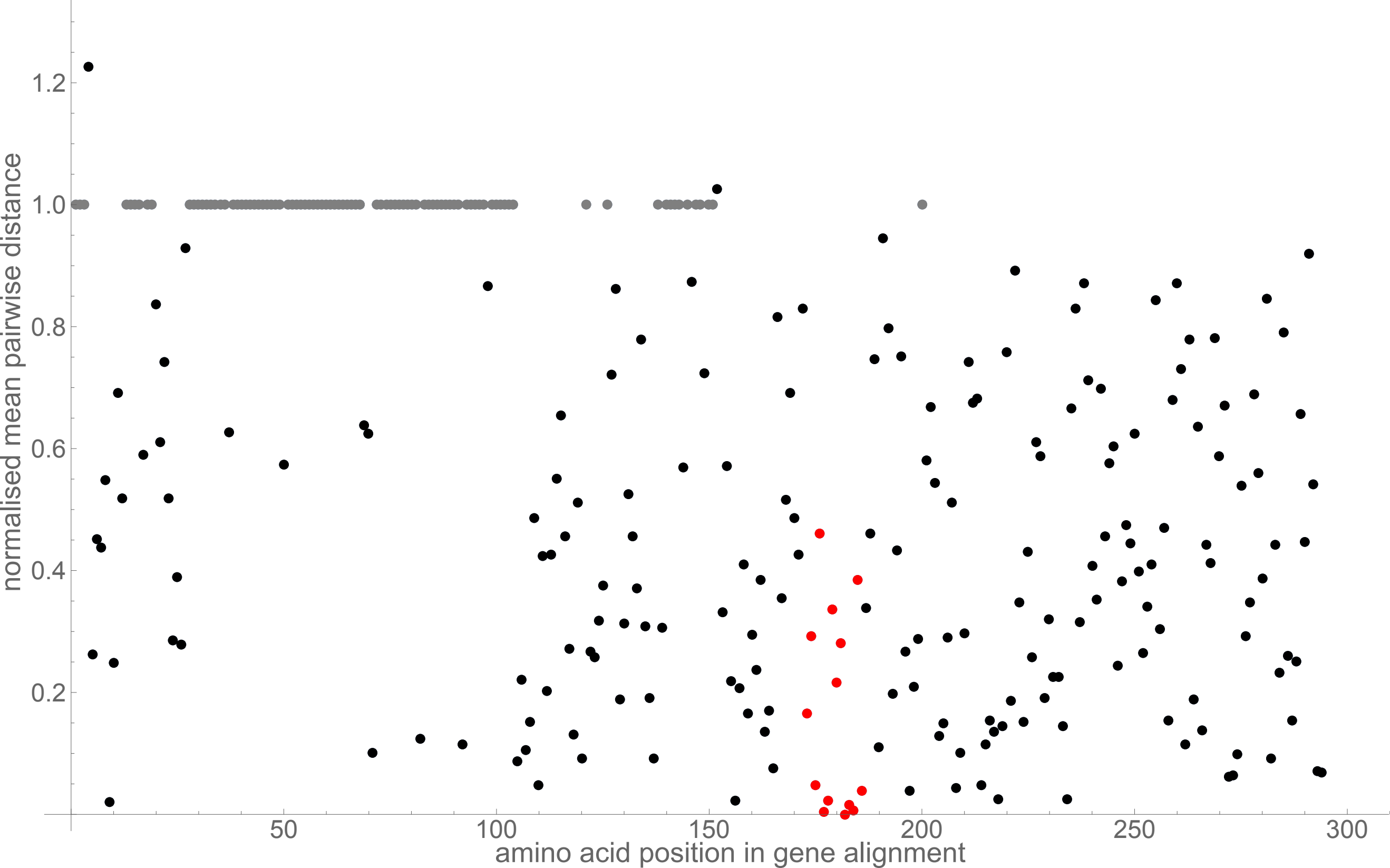

Supplement: S9 Fig — See the caption for S1 Fig for a description of the point colours. (PDF) [file pcbi.1007345.s009.pdf]

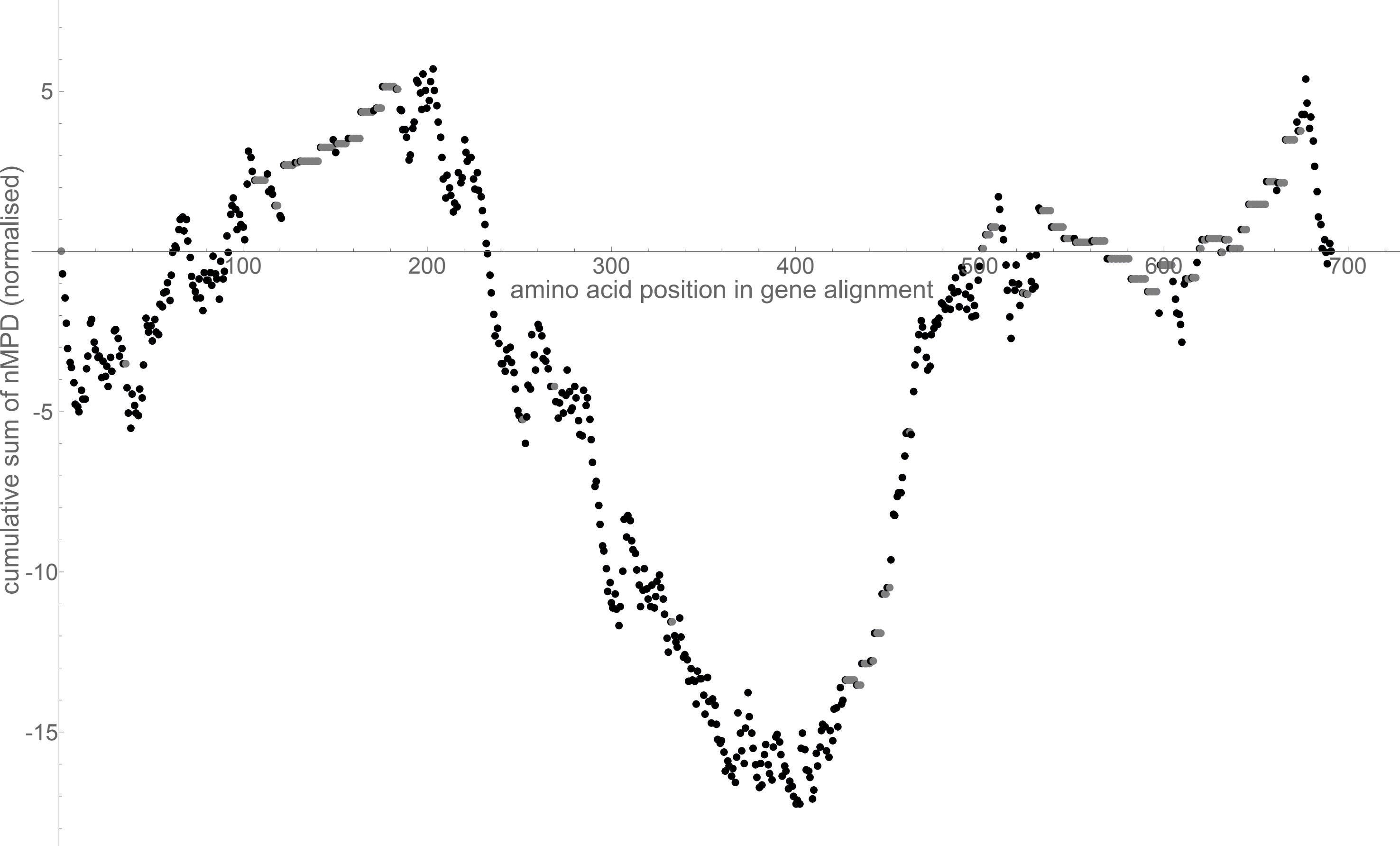

Supplement: S10 Fig — Shown in similar fashion to that of Fig 2. (PDF) [file pcbi.1007345.s010.pdf]

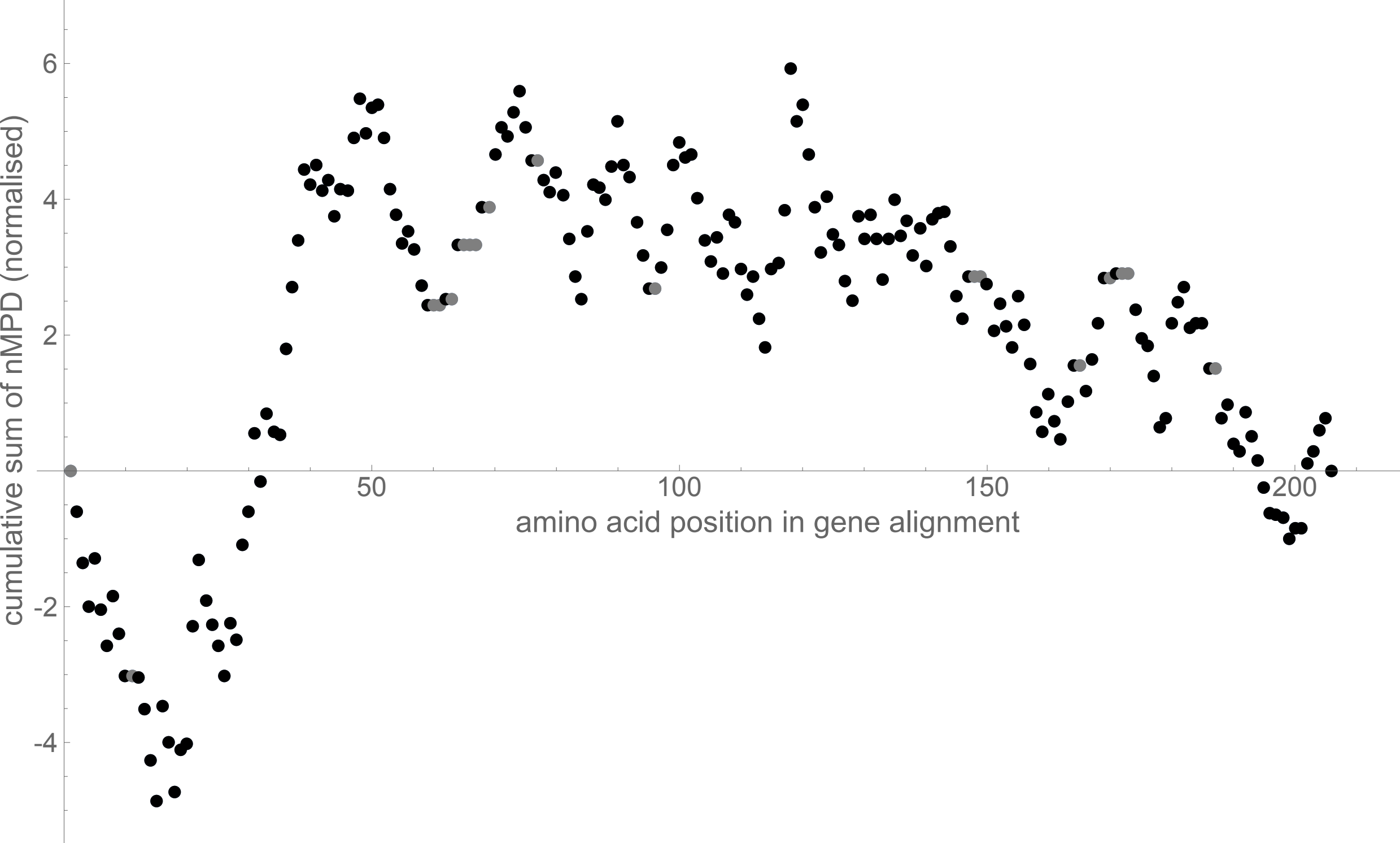

Supplement: S11 Fig — Shown in similar fashion to that of Fig 2. (PDF) [file pcbi.1007345.s011.pdf]

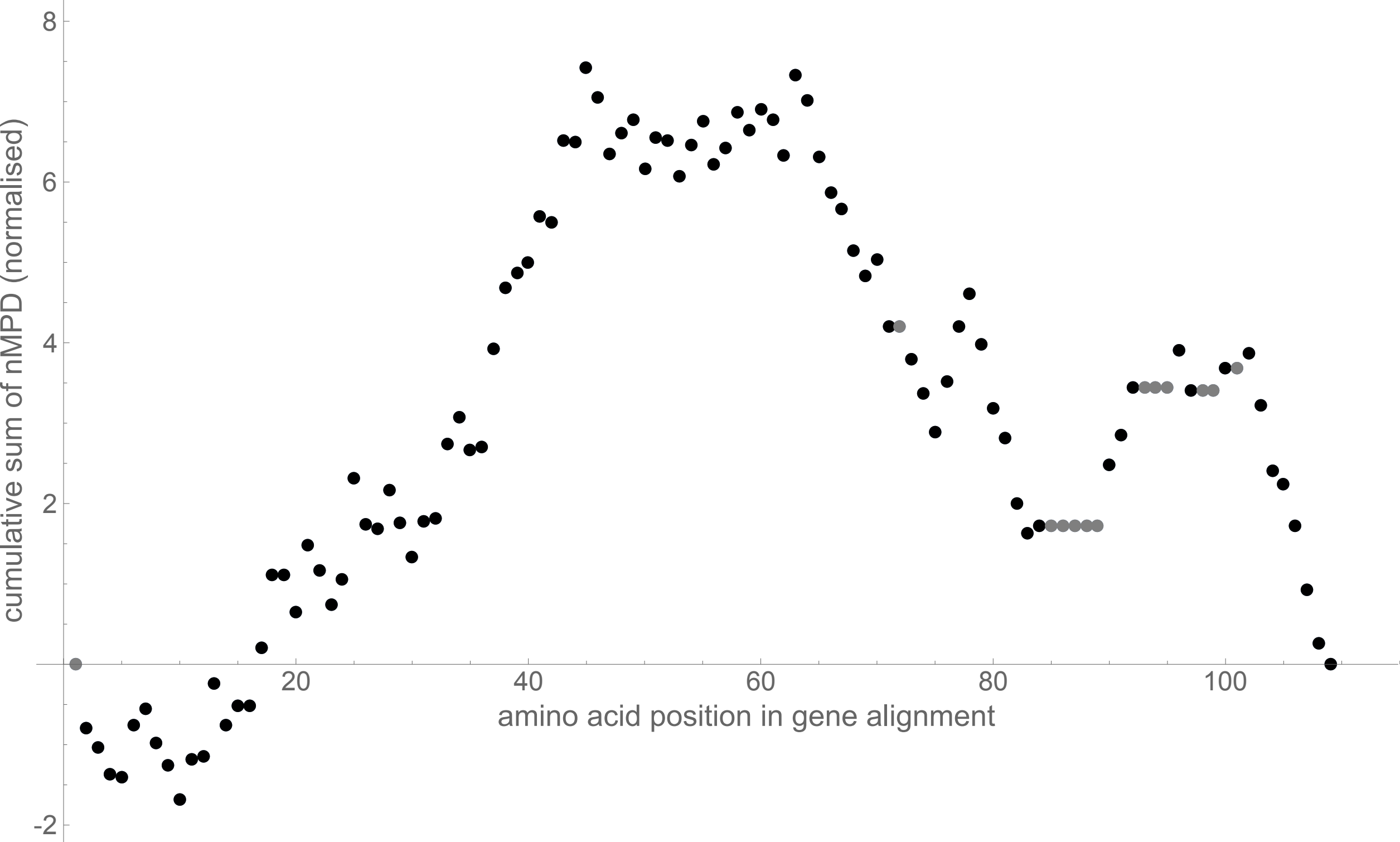

Supplement: S12 Fig — Shown in similar fashion to that of Fig 2. (PDF) [file pcbi.1007345.s012.pdf]

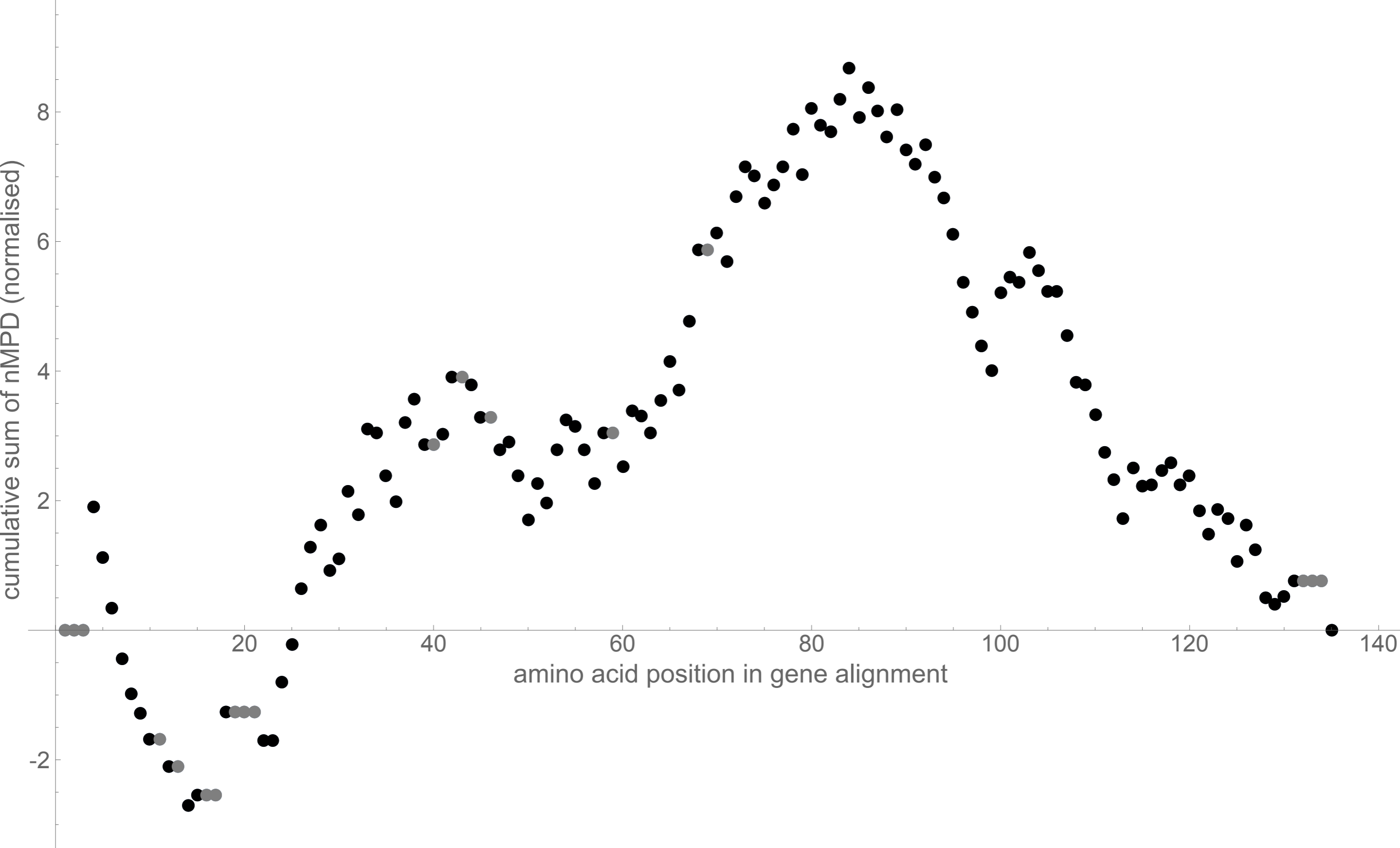

Supplement: S13 Fig — Shown in similar fashion to that of Fig 2. (PDF) [file pcbi.1007345.s013.pdf]

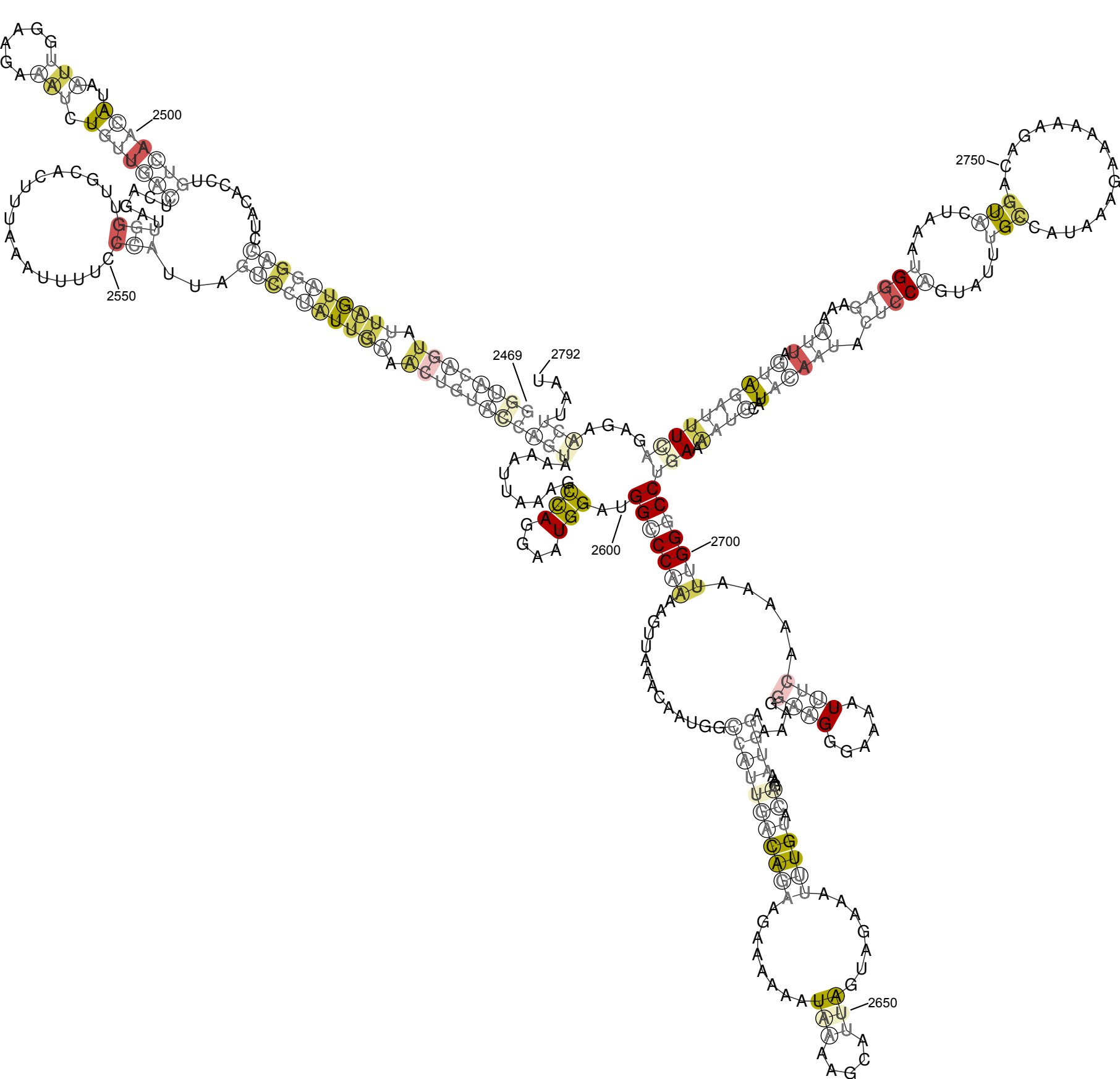

Supplement: S14 Fig — A key to the annotation is given in the caption for Fig 5. (PDF) [file pcbi.1007345.s014.pdf]

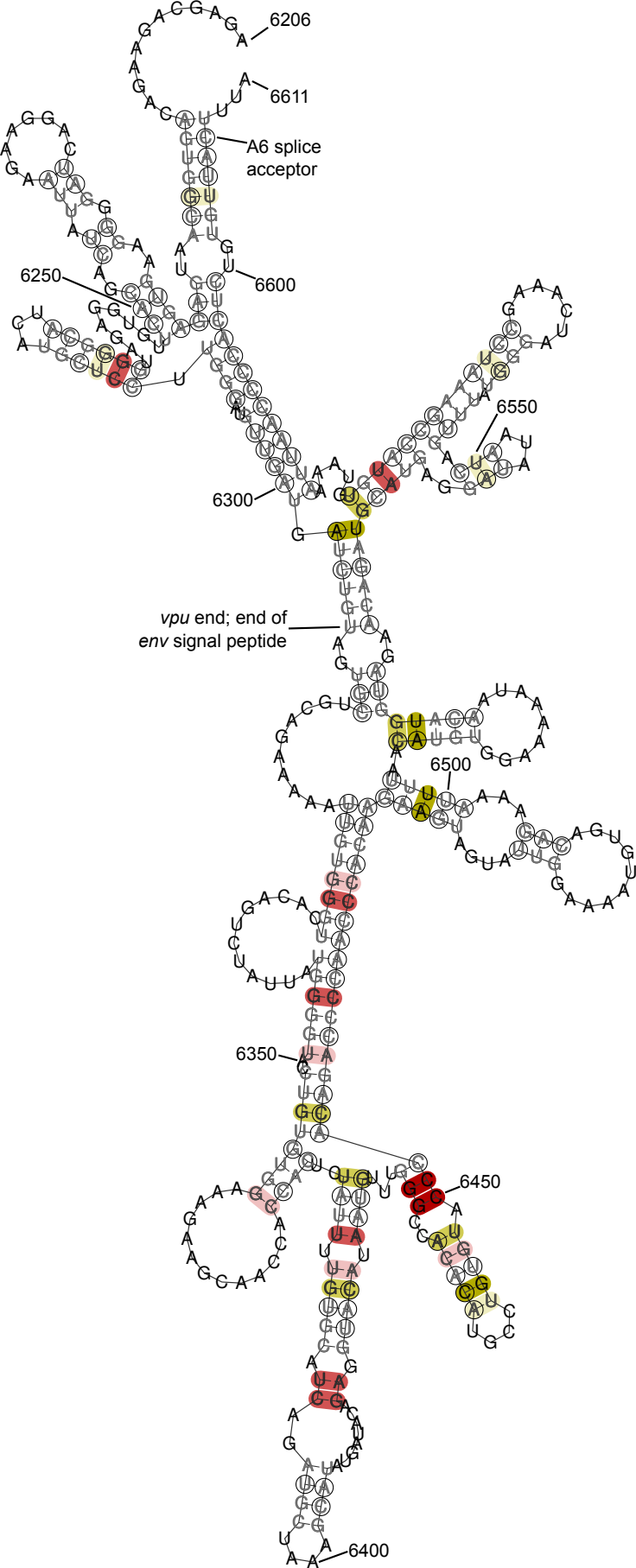

Supplement: S15 Fig — A key to the annotation is given in the caption for Fig 5. (PDF) [file pcbi.1007345.s015.pdf]

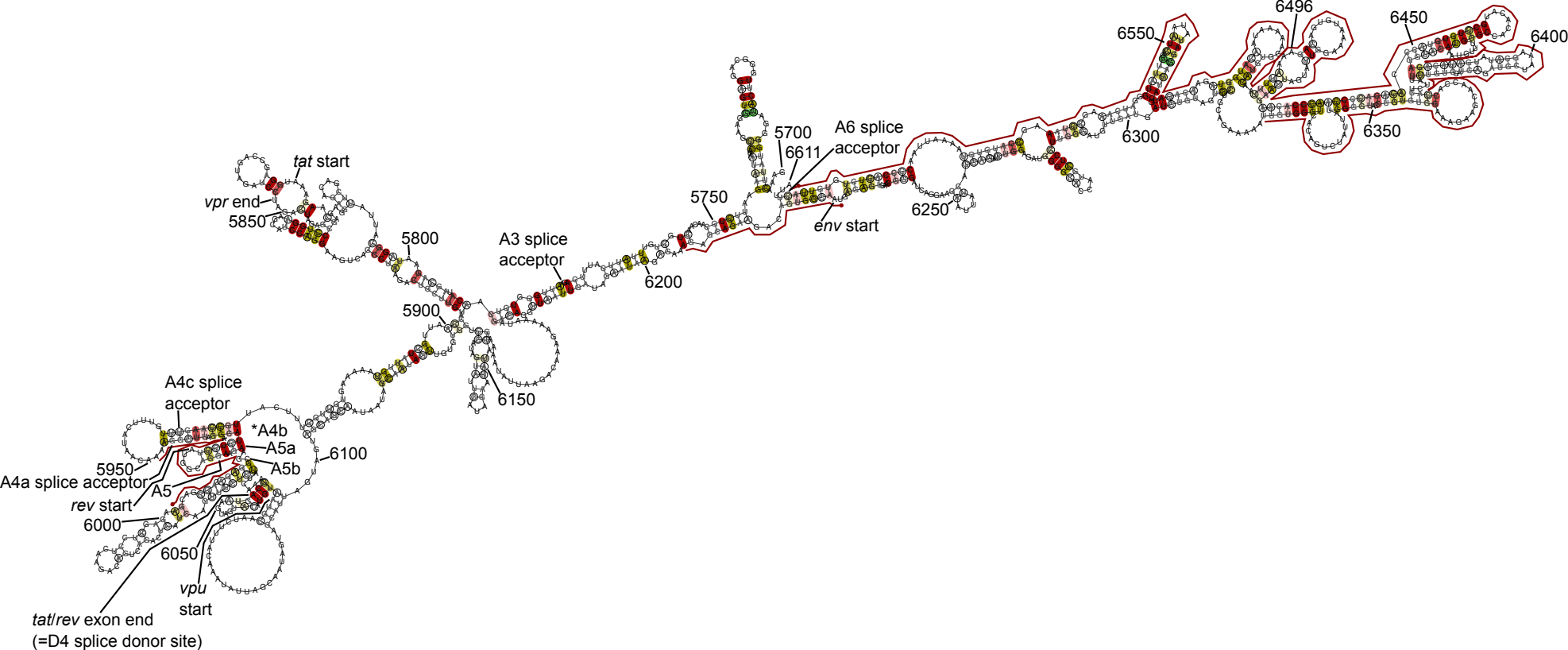

Supplement: S16 Fig — Subregions of interest are represented by red lines adjacent to the regions’ respective nucleotides. A key to the annotation is given in the caption for Fig 5. A GAR exon splicing enhancer overlapping a region of interest is present in the region 5977–6002, but is not included in the labels for clarity. (PDF) [file pcbi.1007345.s016.pdf]

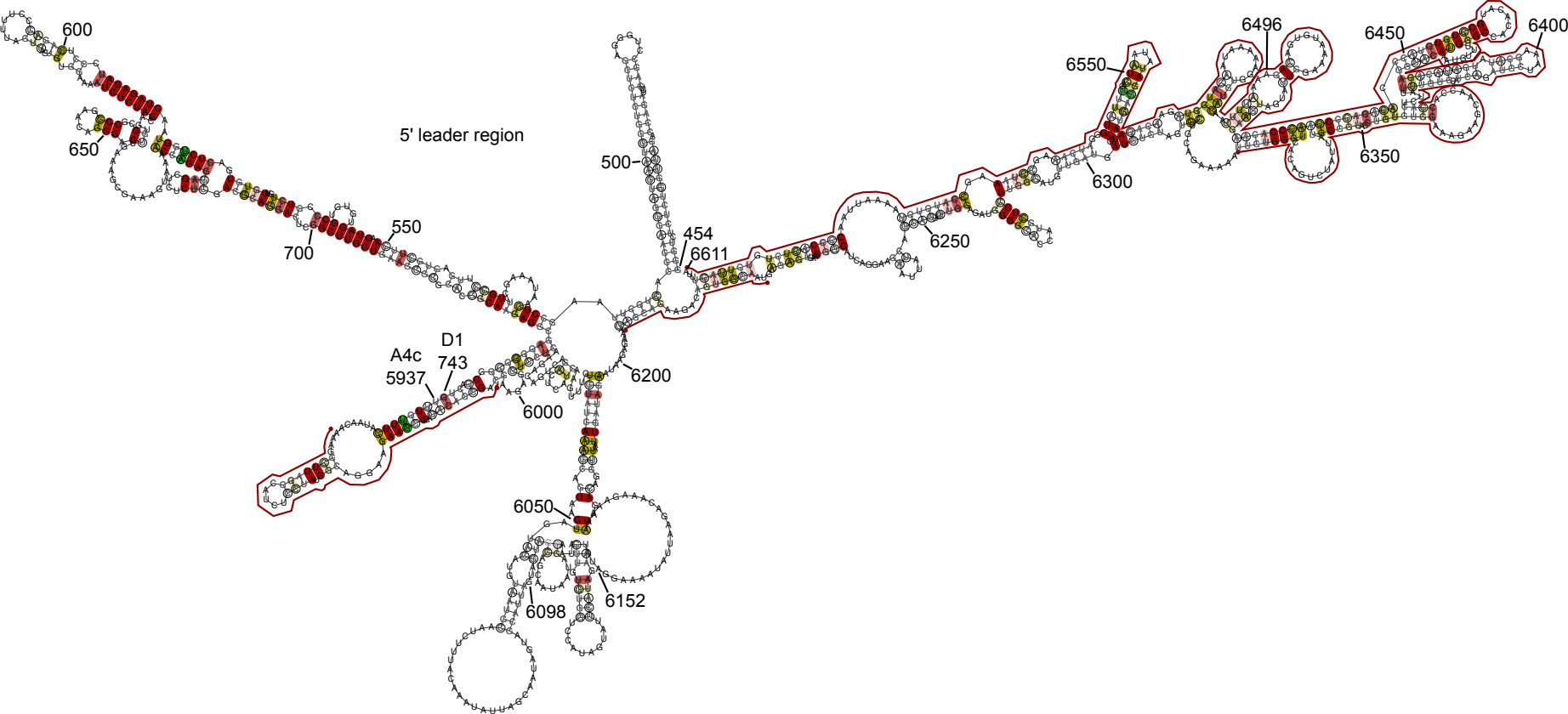

Supplement: S17 Fig — A key to the annotation is given in the caption for Fig 5. Additional features are labelled in S16 Fig, but are omitted here for clarity. (PDF) [file pcbi.1007345.s017.pdf]

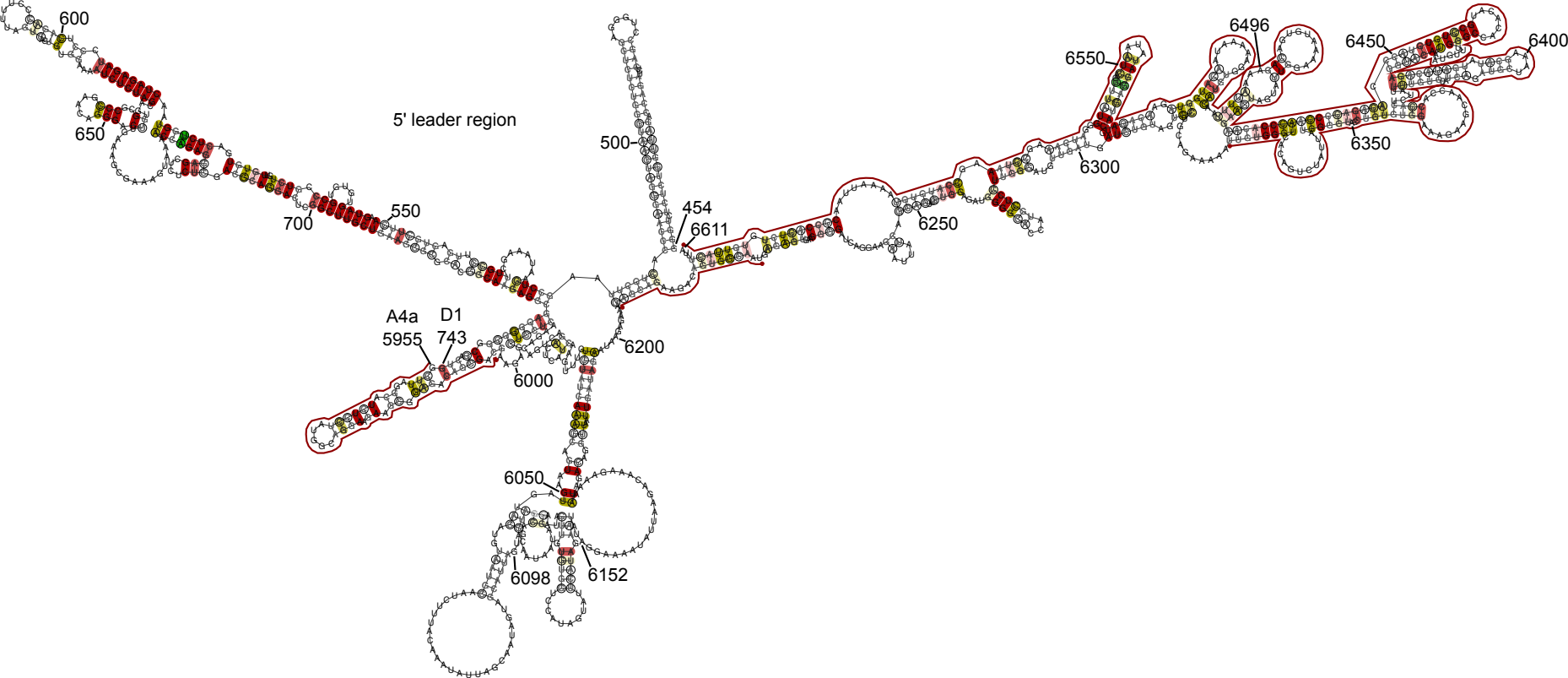

Supplement: S18 Fig — A key to the annotation is given in the caption for Fig 5. Additional features are labelled in S16 Fig, but are omitted here for clarity. (PDF) [file pcbi.1007345.s018.pdf]

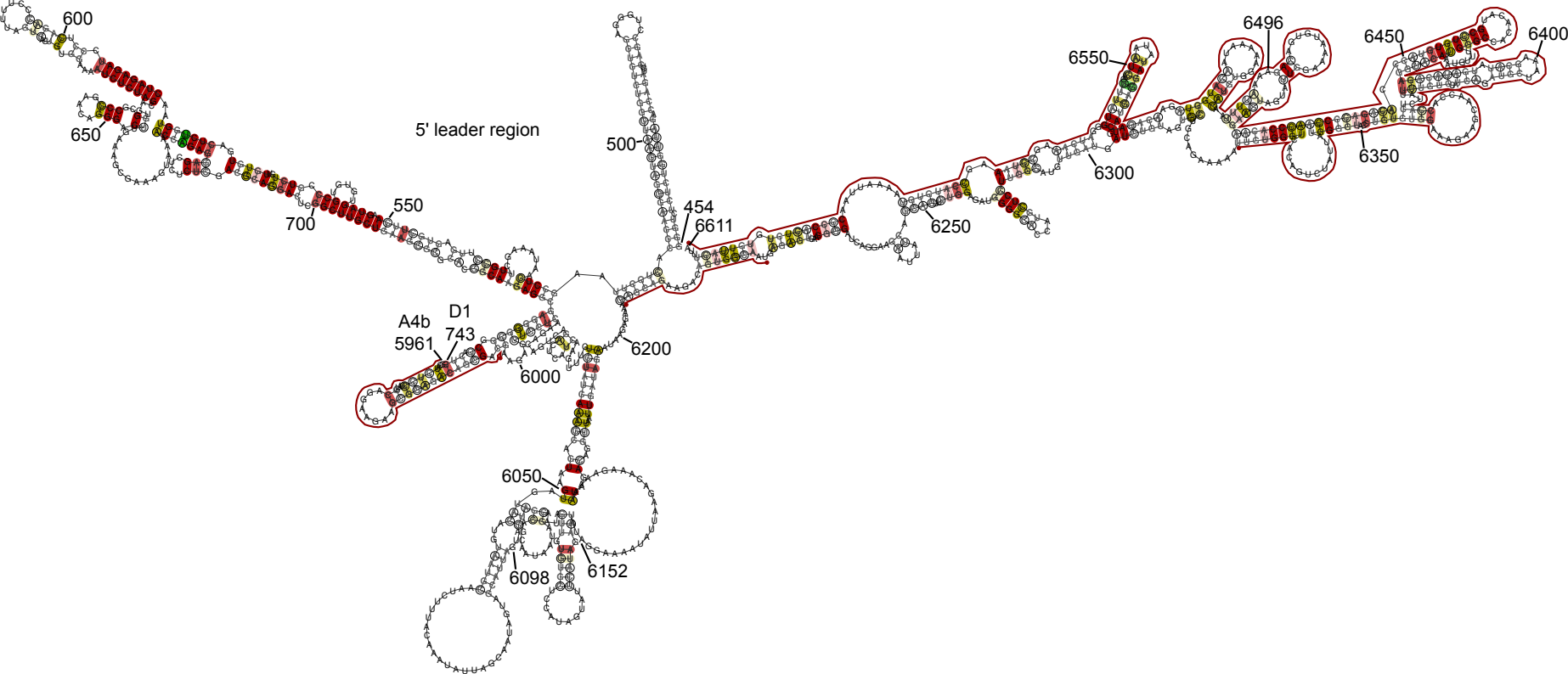

Supplement: S19 Fig — A key to the annotation is given in the caption for Fig 5. Additional features are labelled in, but are omitted here for clarity. (PDF) [file pcbi.1007345.s019.pdf]

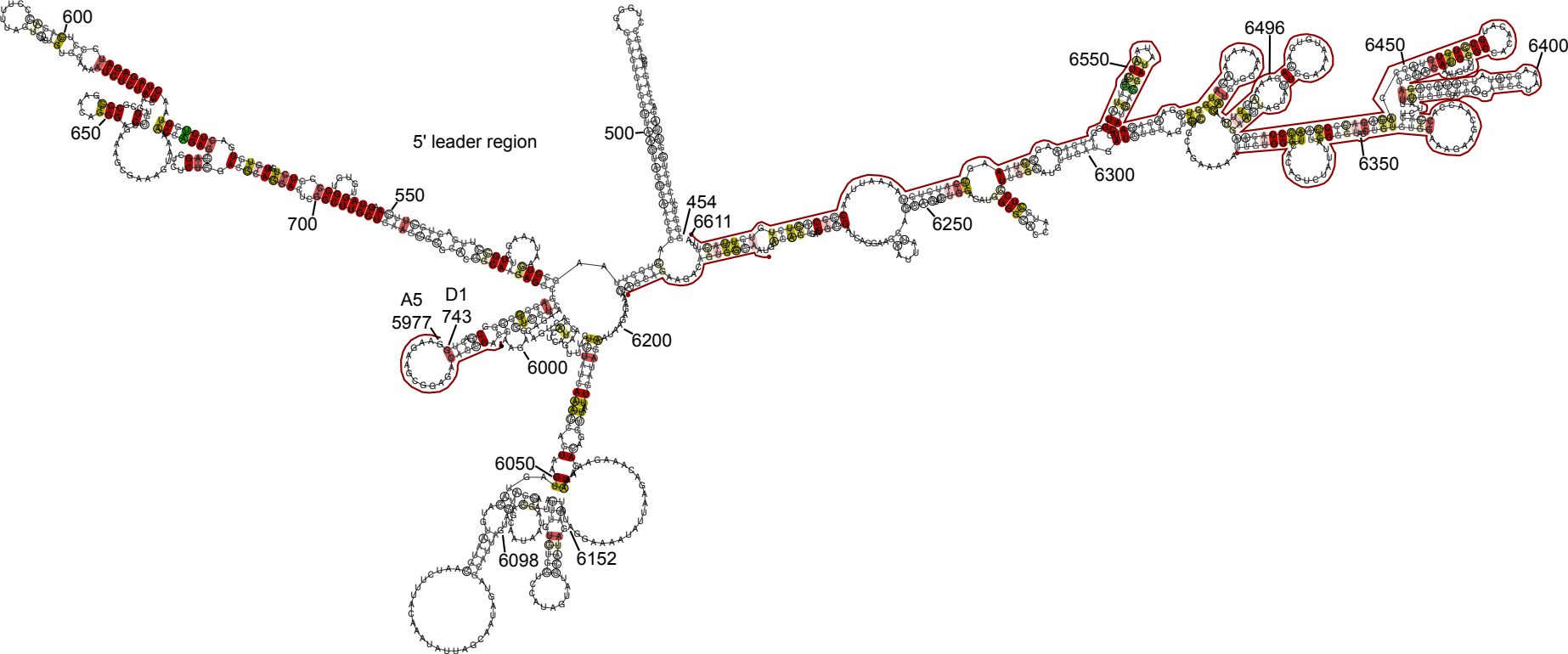

Supplement: S20 Fig — A key to the annotation is given in the caption for Fig 5. Additional features are labelled in S16 Fig, but are omitted here for clarity. (PDF) [file pcbi.1007345.s020.pdf]

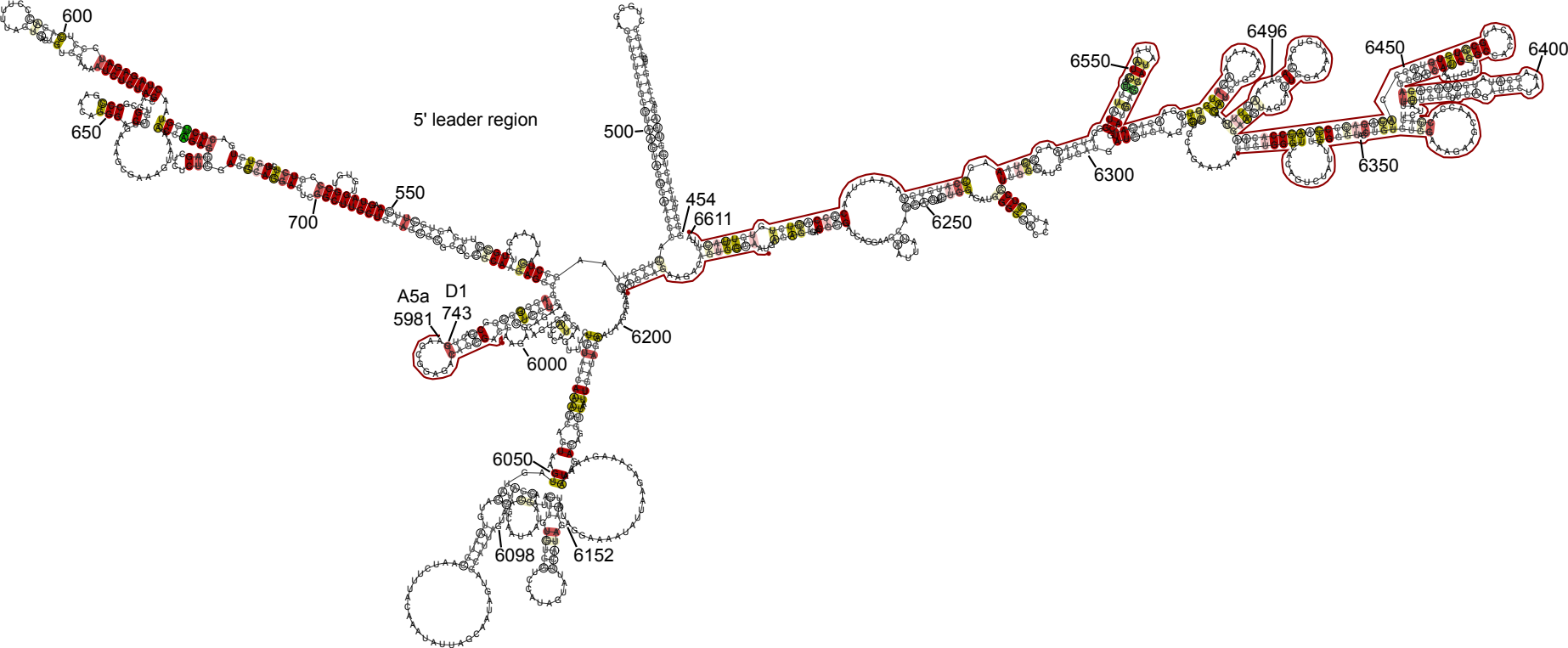

Supplement: S21 Fig — A key to the annotation is given in the caption for Fig 5. Additional features are labelled in S16 Fig, but are omitted here for clarity. (PDF) [file pcbi.1007345.s021.pdf]

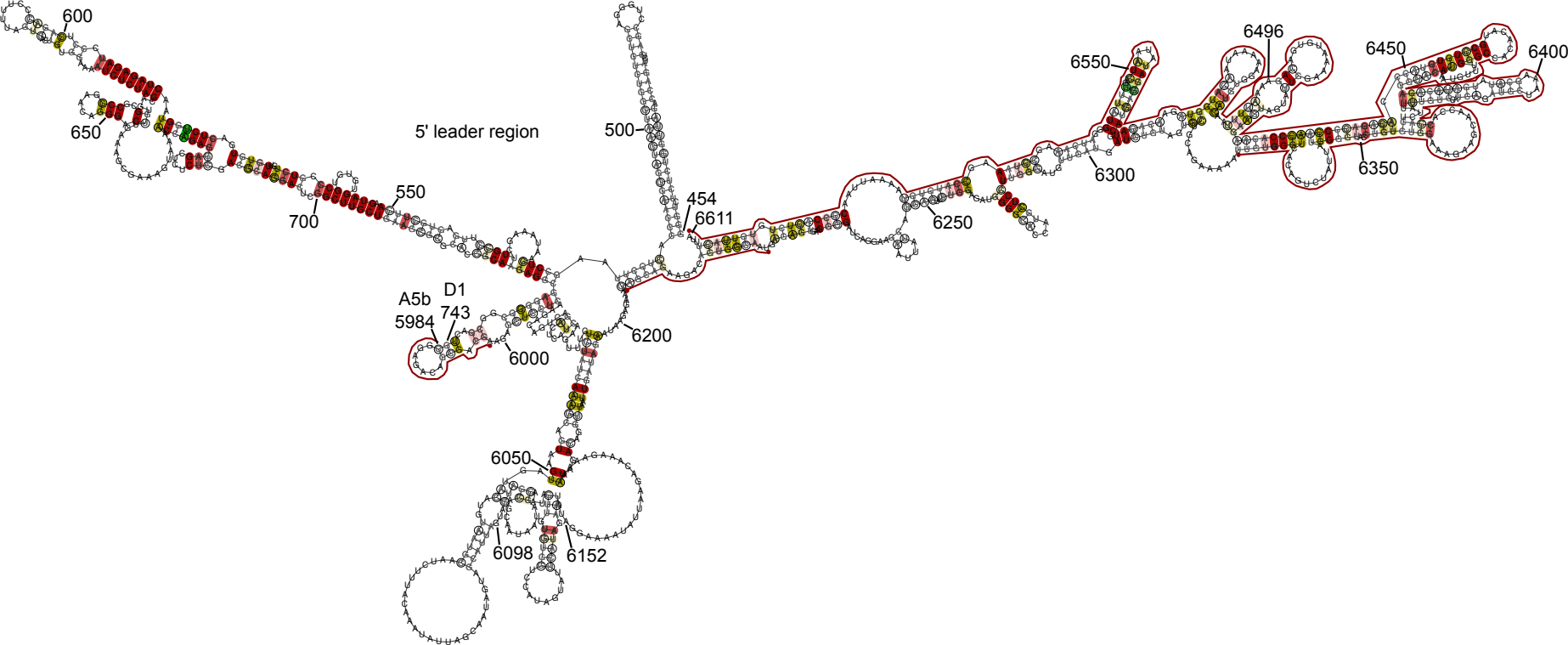

Supplement: S22 Fig — A key to the annotation is given in the caption for Fig 5. Additional features are labelled in S16 Fig, but are omitted here for clarity. (PDF) [file pcbi.1007345.s022.pdf]

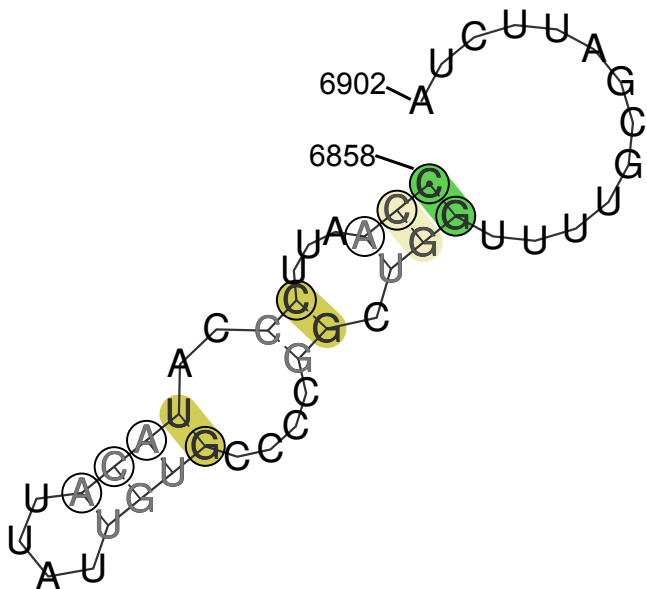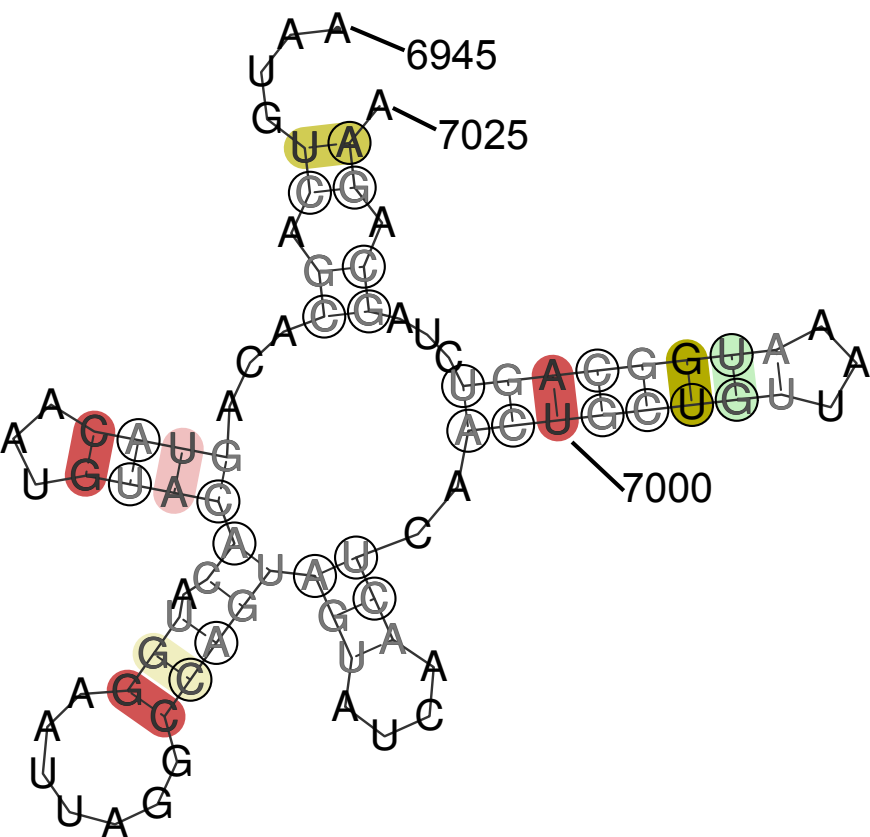

Supplement: S23 Fig — Top: region corresponding to HXB2 reference 6858–6902. Bottom: region corresponding to HXB2 reference 6945–7025. The modified free energies [17, 71] for these predicted structures are −12.99kcal/mol and −33.06kcal/mol, respectively. A key to the annotation is given in the caption for Fig 5. (PDF) [file pcbi.1007345.s023.pdf]

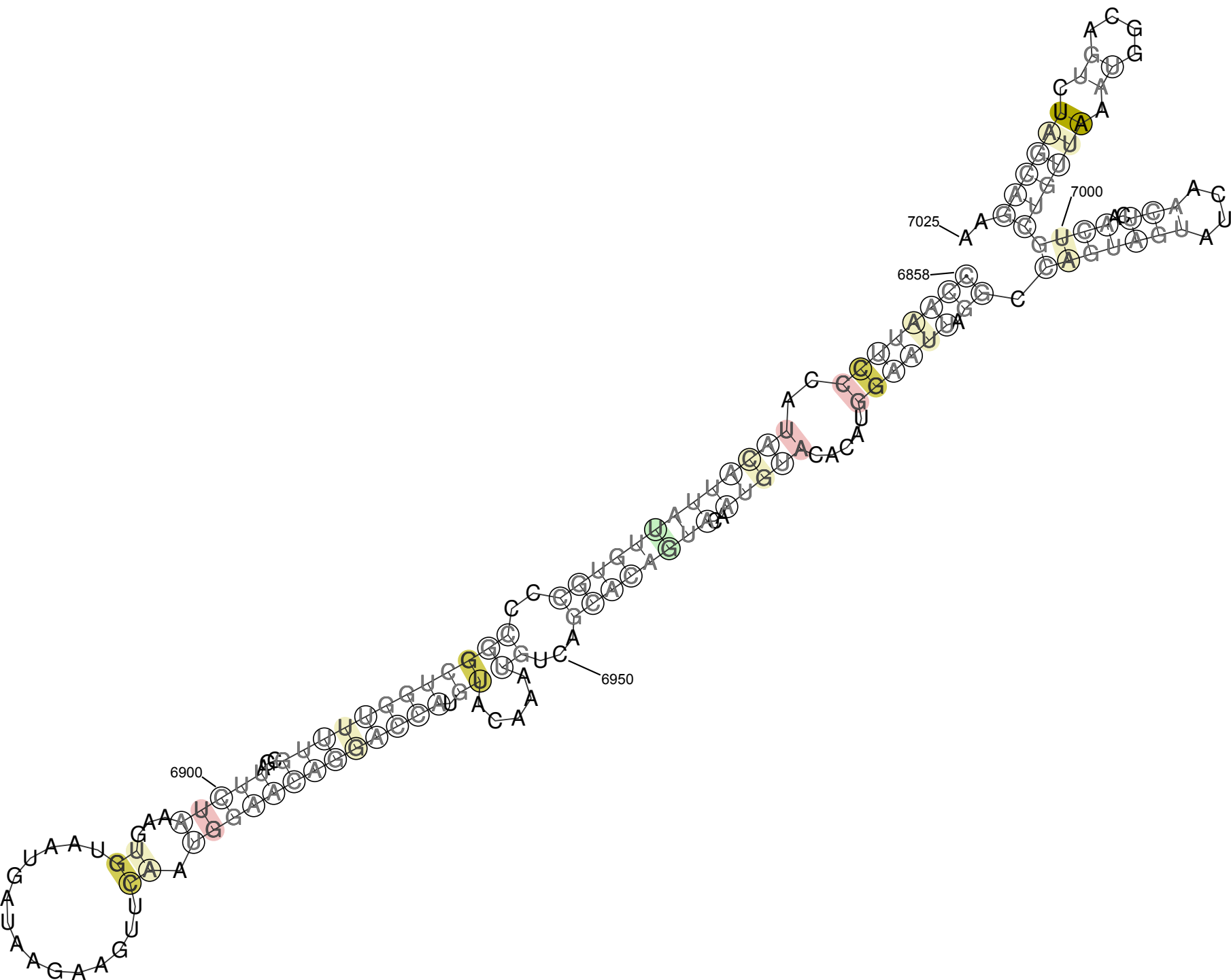

Supplement: S24 Fig — The modified free energy [17, 71] for this larger predicted structure is −75.42kcal/mol, substantially lower than the sum of the modified free energies of the smaller predicted structures (−46.05kcal/mol; this does not take into account the region 6903–6944, which is small in comparison with the overall region size). A key to the annotation is given in the caption for Fig 5. (PDF) [file pcbi.1007345.s024.pdf]

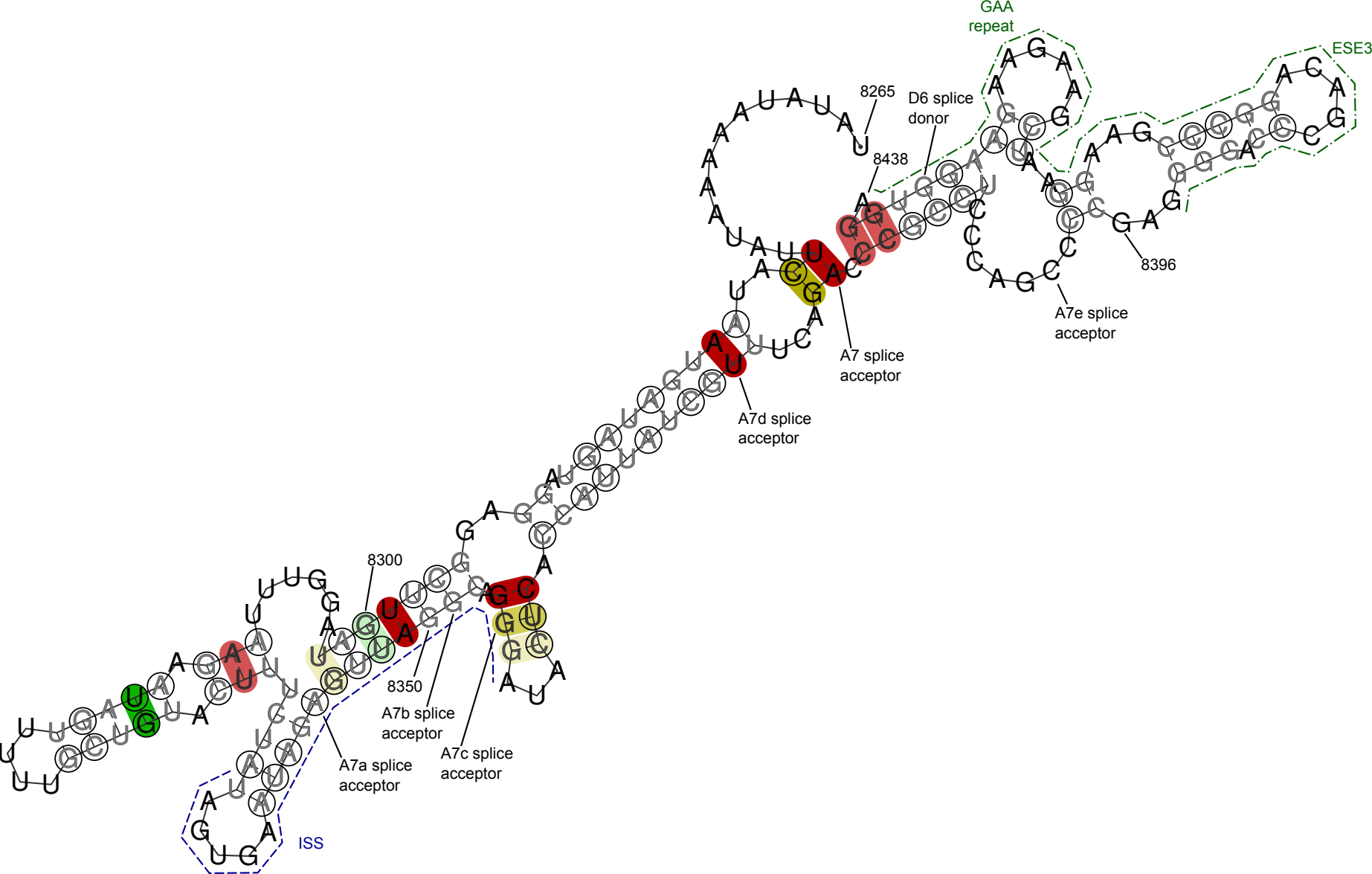

Supplement: S25 Fig — A key to the annotation is given in the captions for Figs 5 and 6. The sequence with GenBank accession number JQ403098 was not used to produce this fold as its sequenced region and the region of interest do not overlap. (PDF) [file pcbi.1007345.s025.pdf]

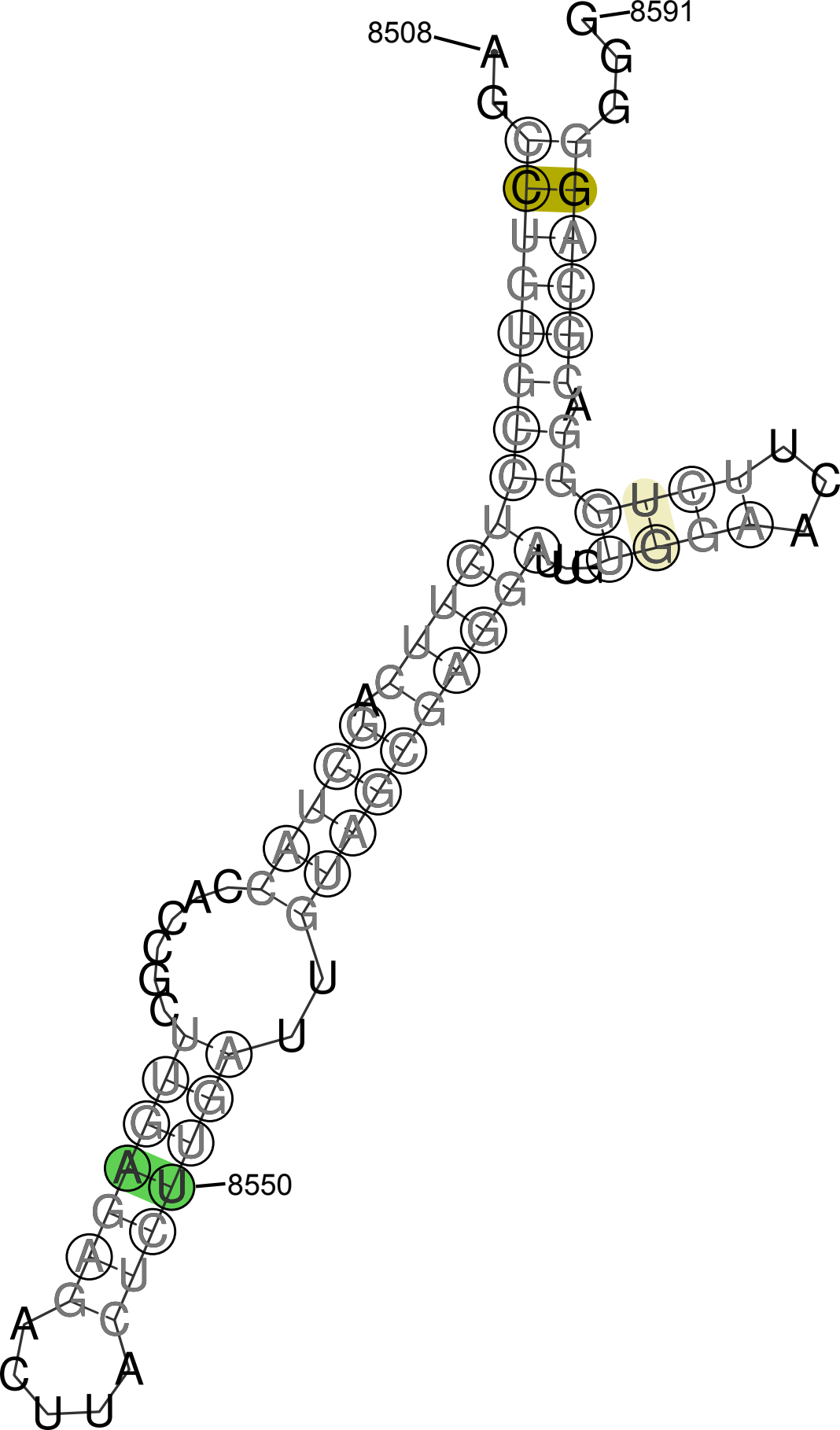

Supplement: S26 Fig — A key to the annotation is given in the caption for Fig 5. The sequence with GenBank accession number JQ403098 was not used to produce this fold as its sequenced region and the region of interest do not overlap. (PDF) [file pcbi.1007345.s026.pdf]

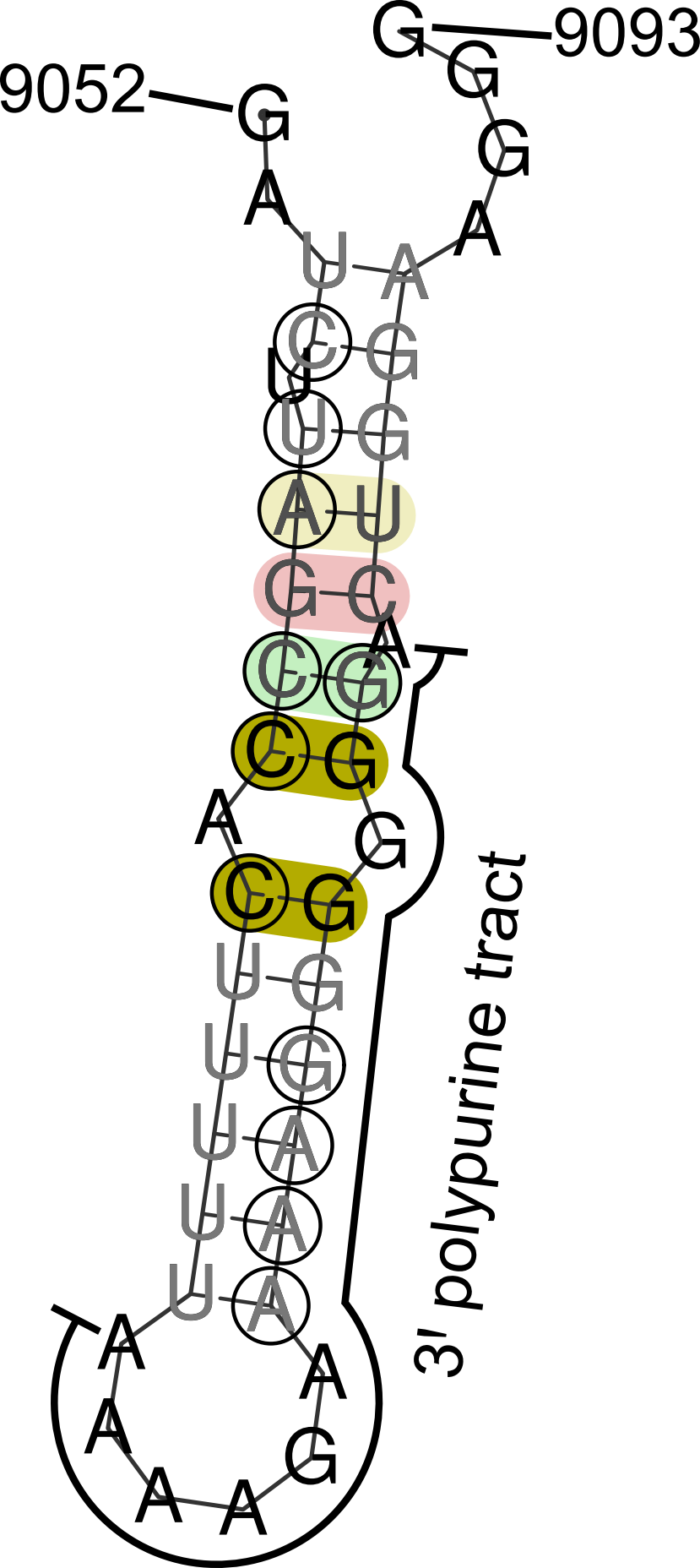

Supplement: S27 Fig — A key to the annotation is given in the caption for Fig 5. Some sequences were not used when producing this fold: see S23 Table for more information. (PDF) [file pcbi.1007345.s027.pdf]

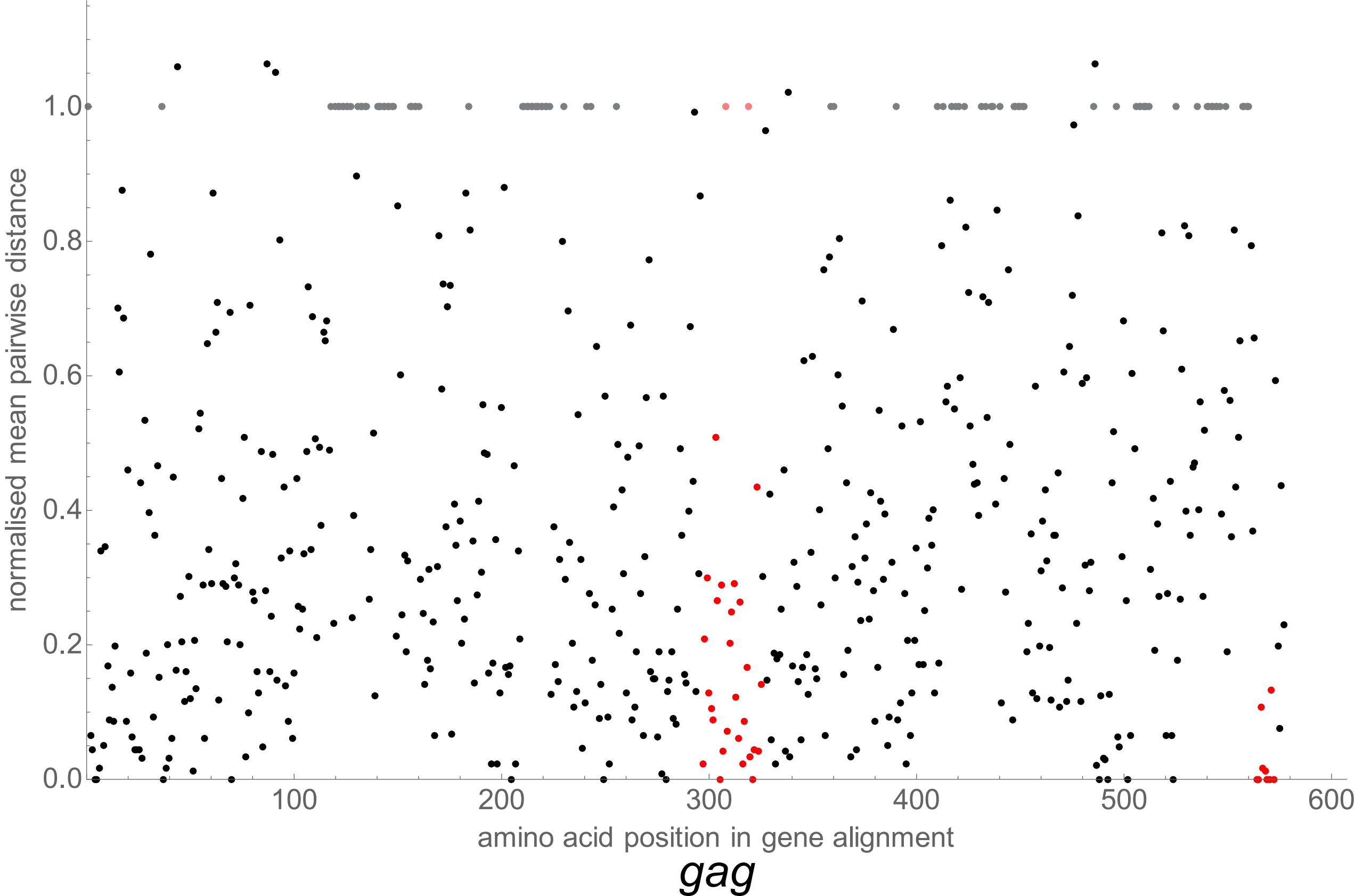

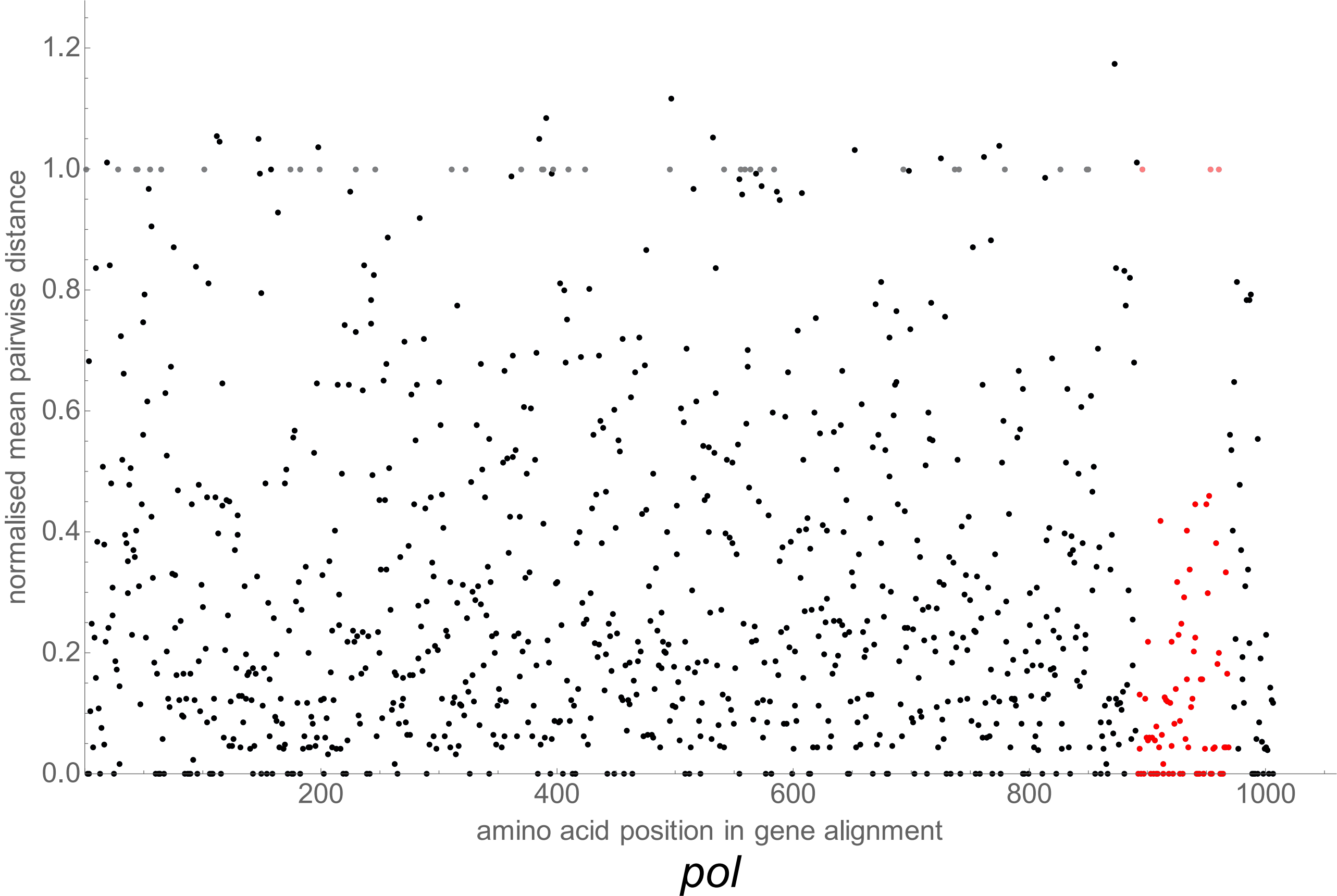

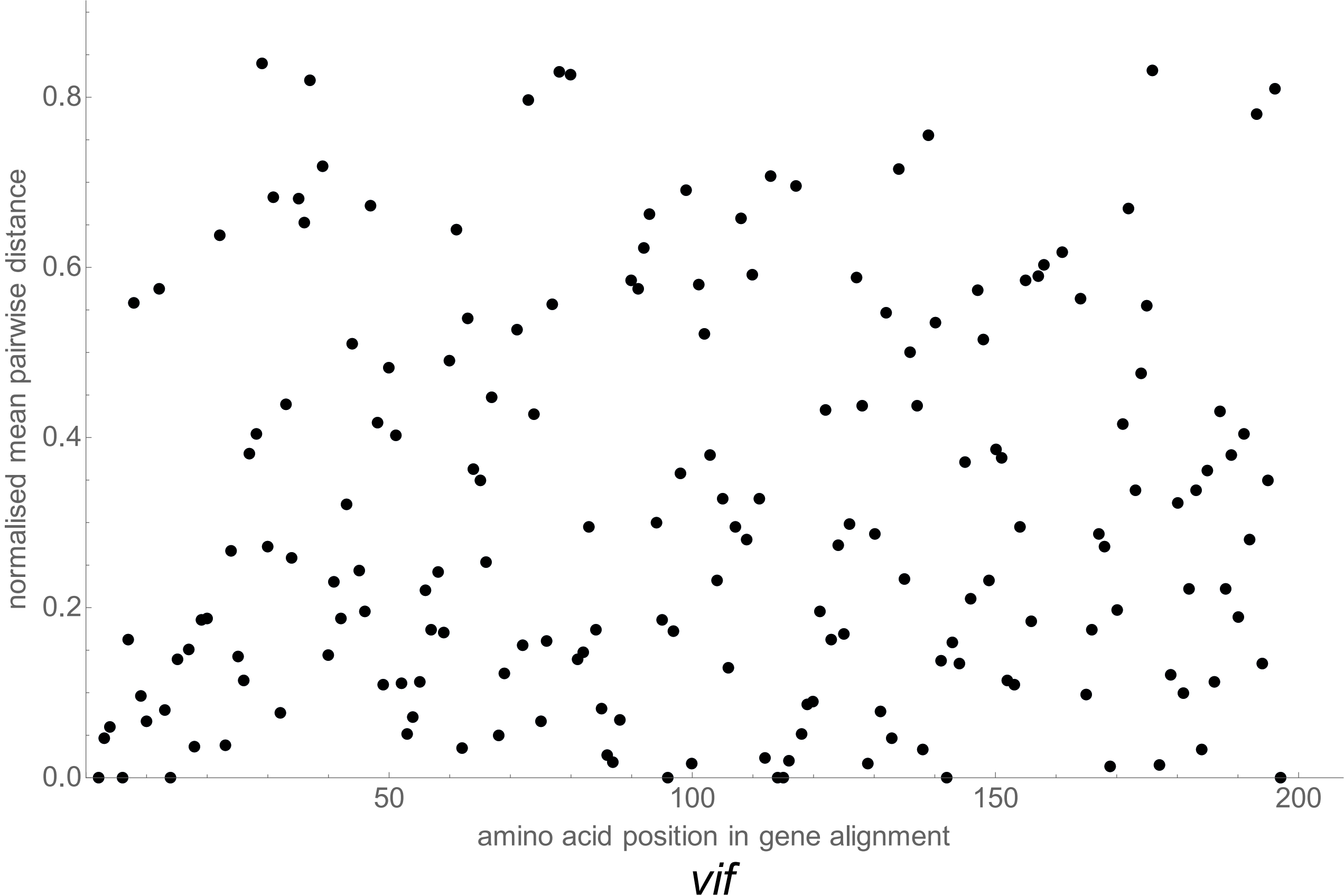

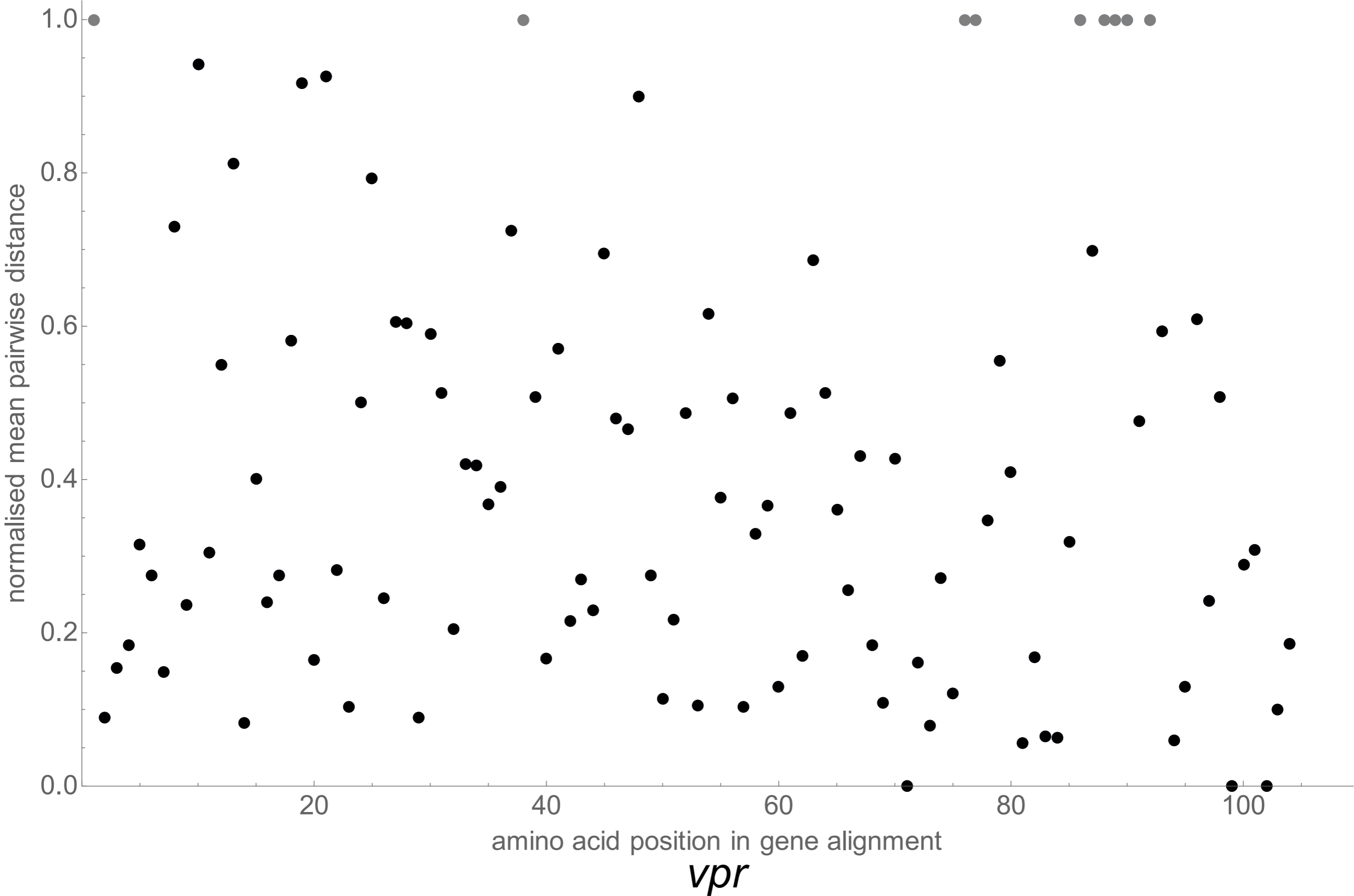

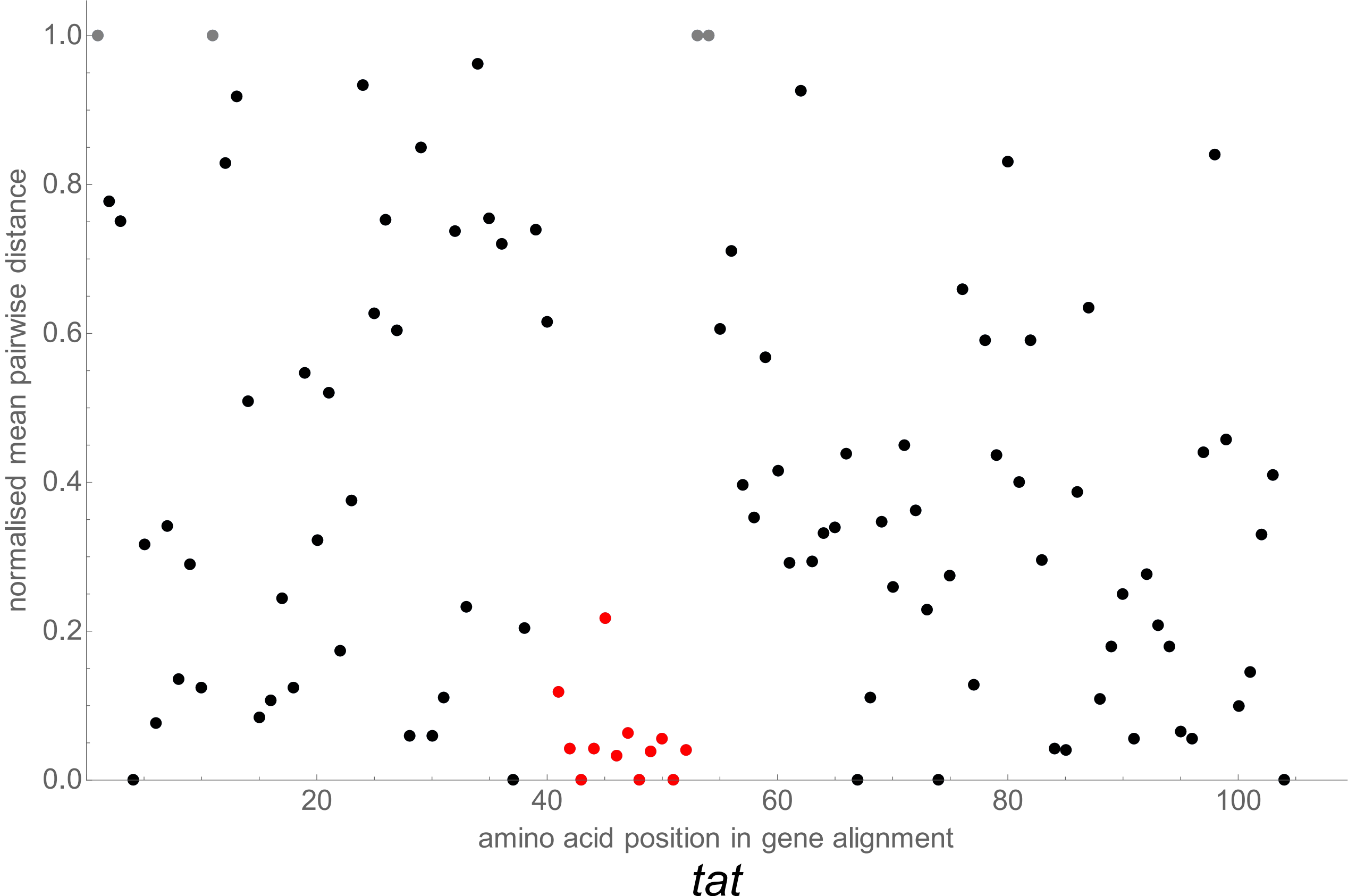

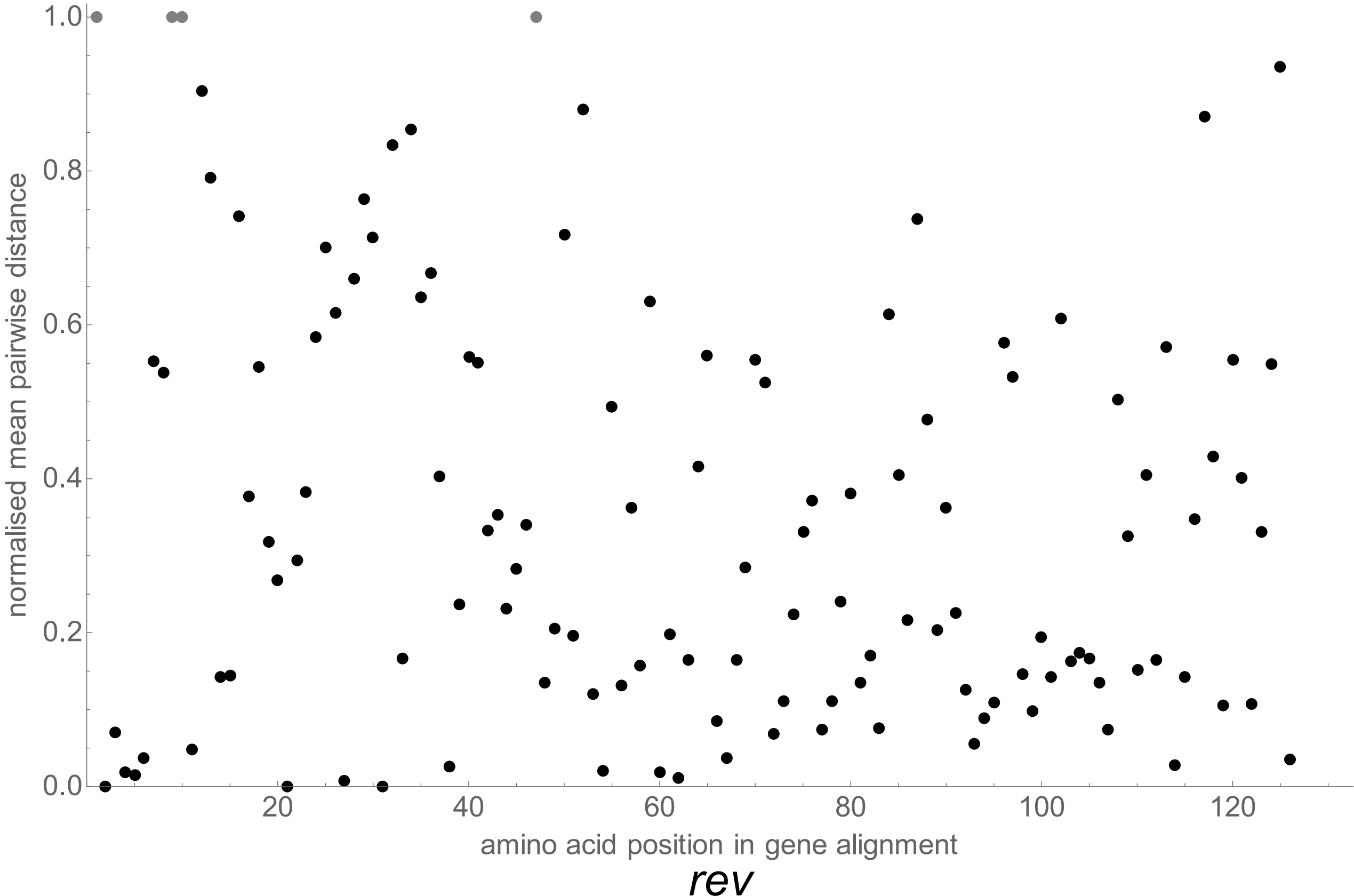

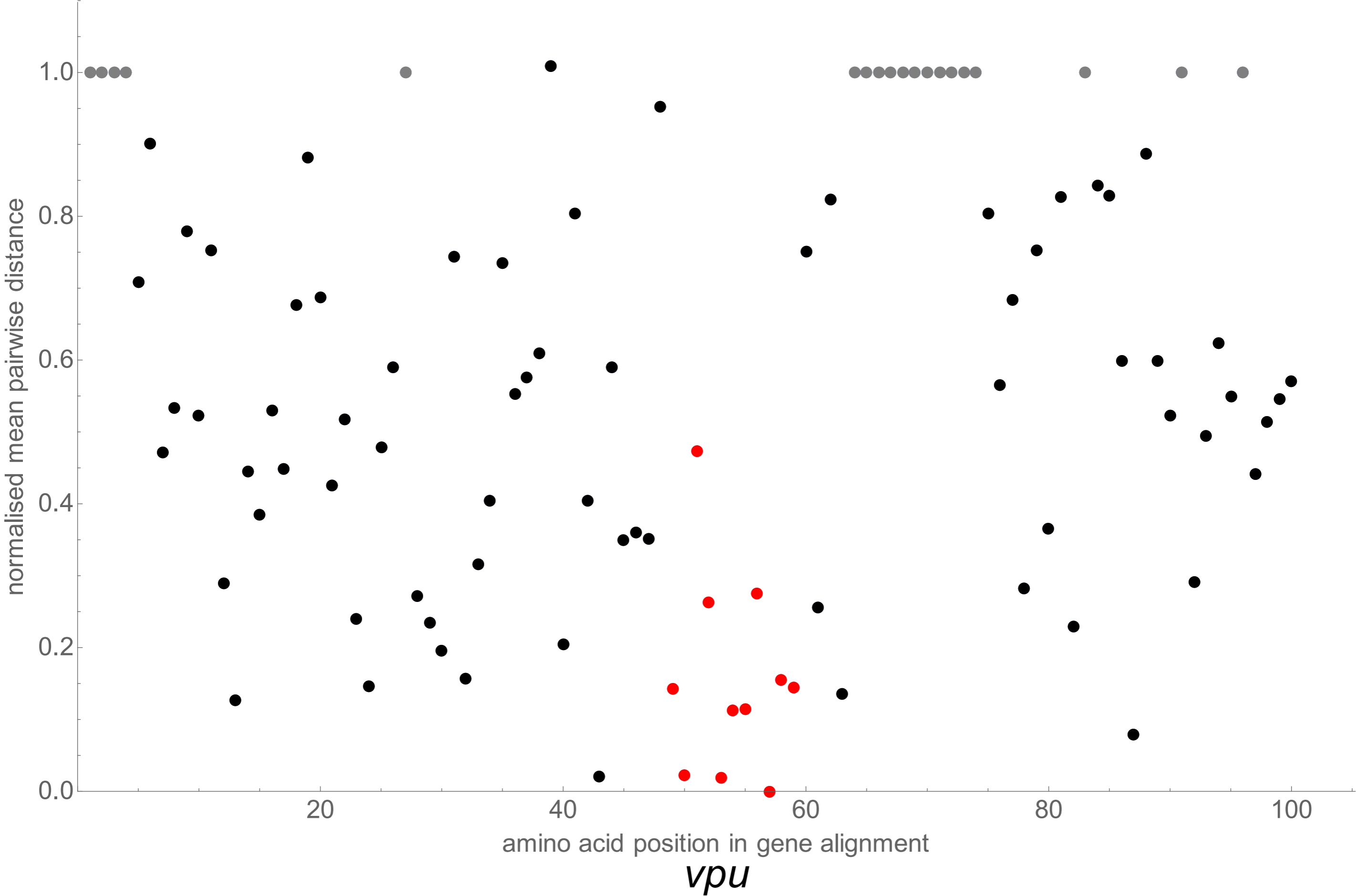

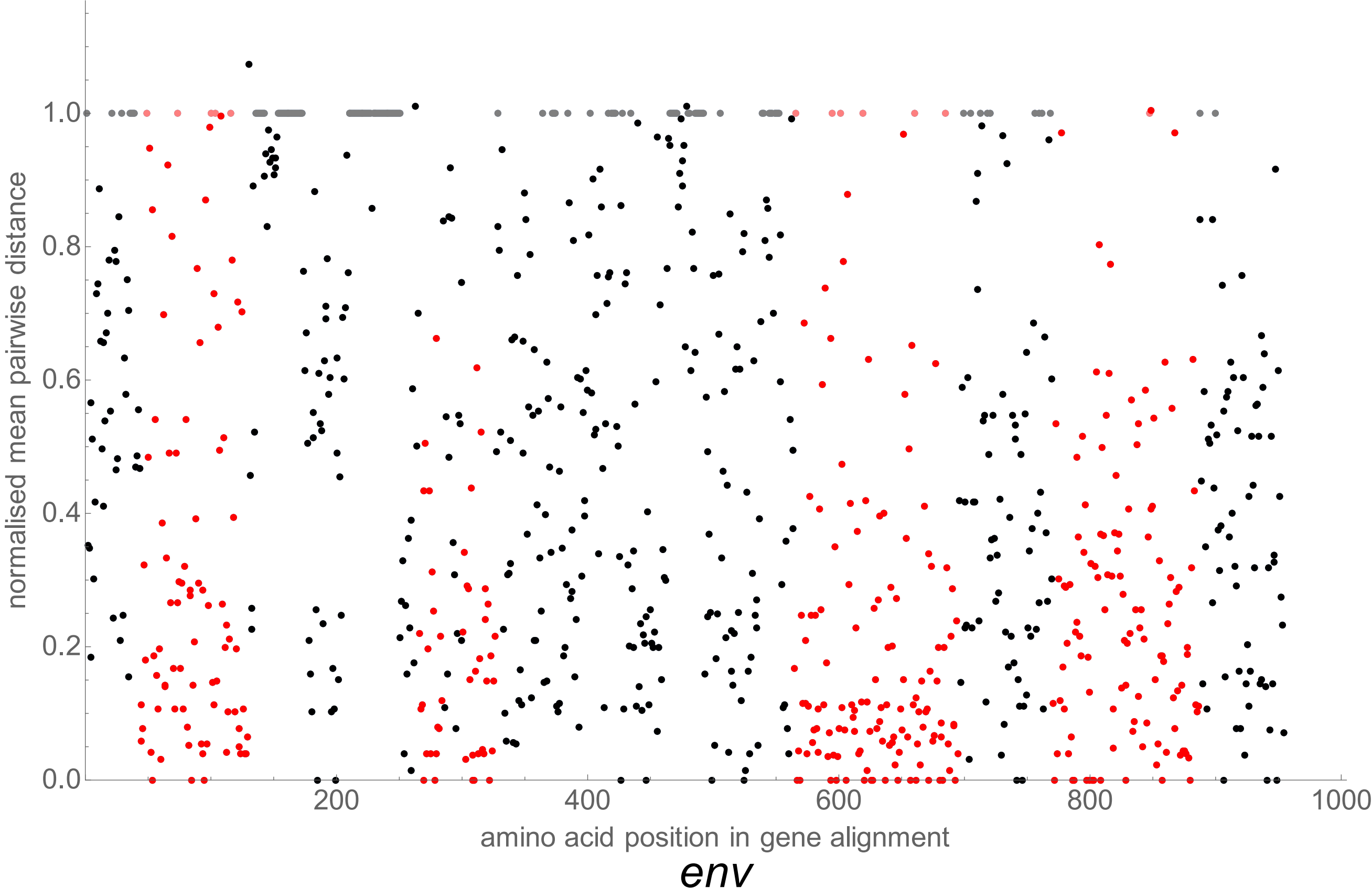

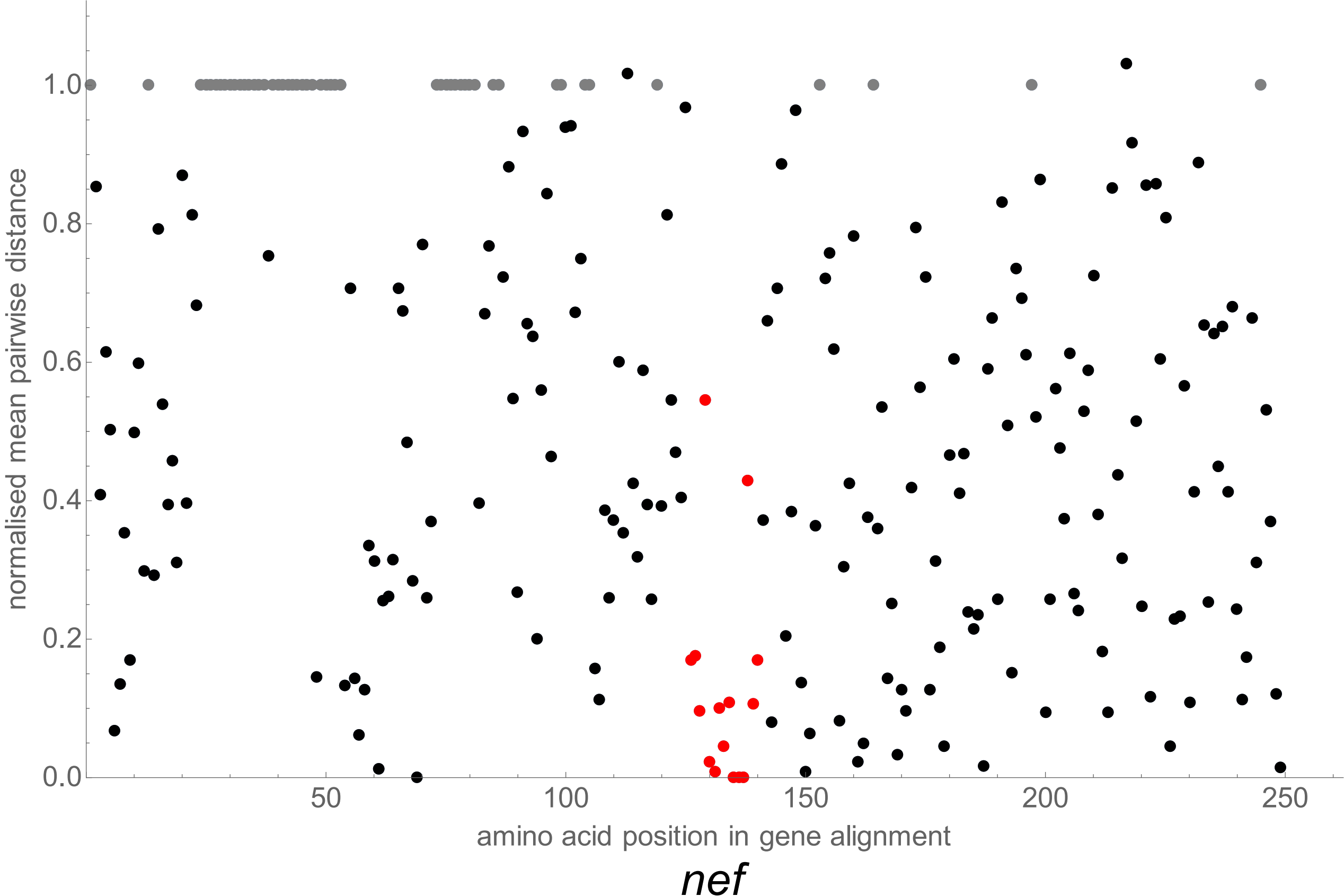

Supplement: S28 Fig — See the caption for S1 Fig for a description of the point colours. (PDF) [file pcbi.1007345.s028.pdf]

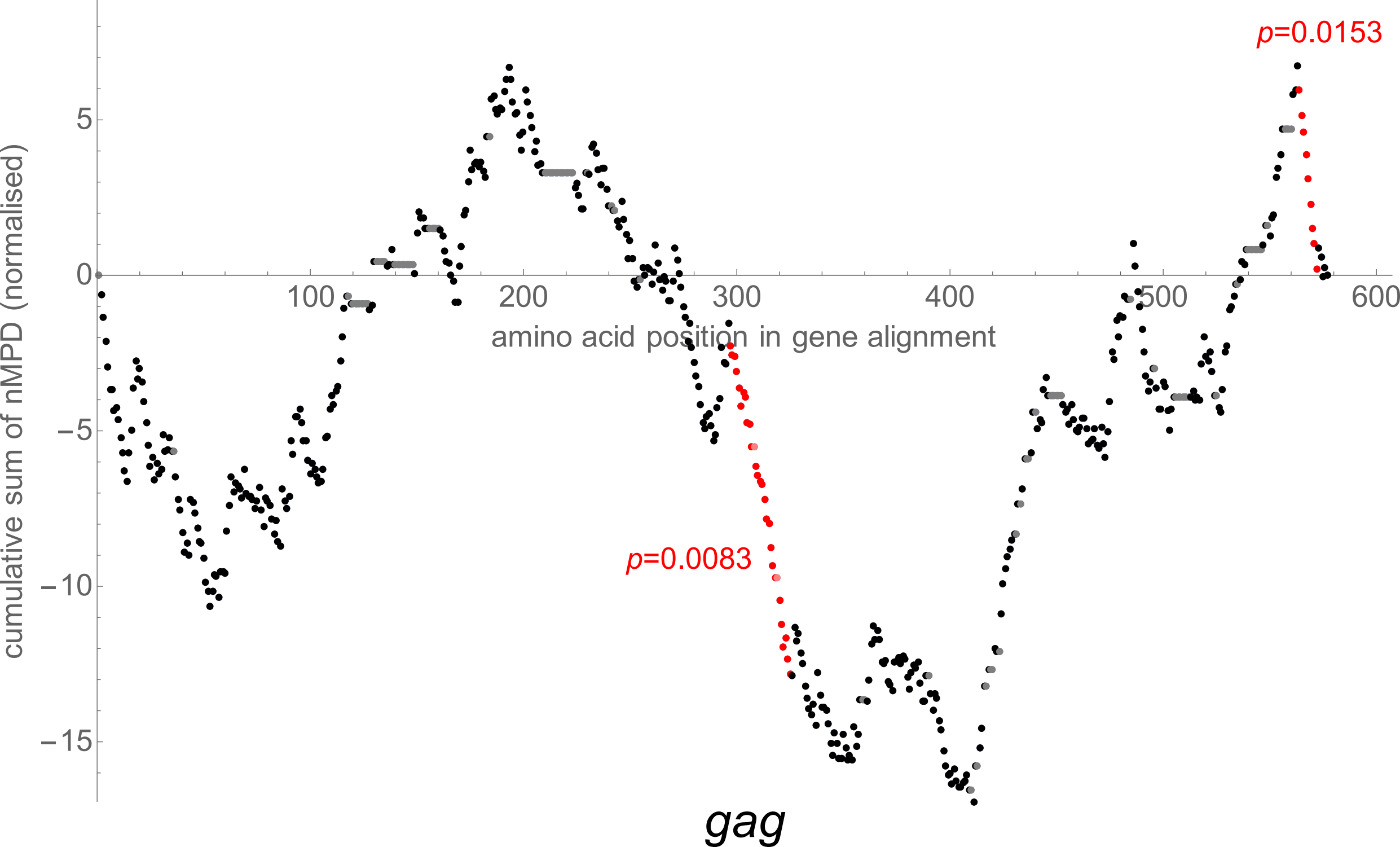

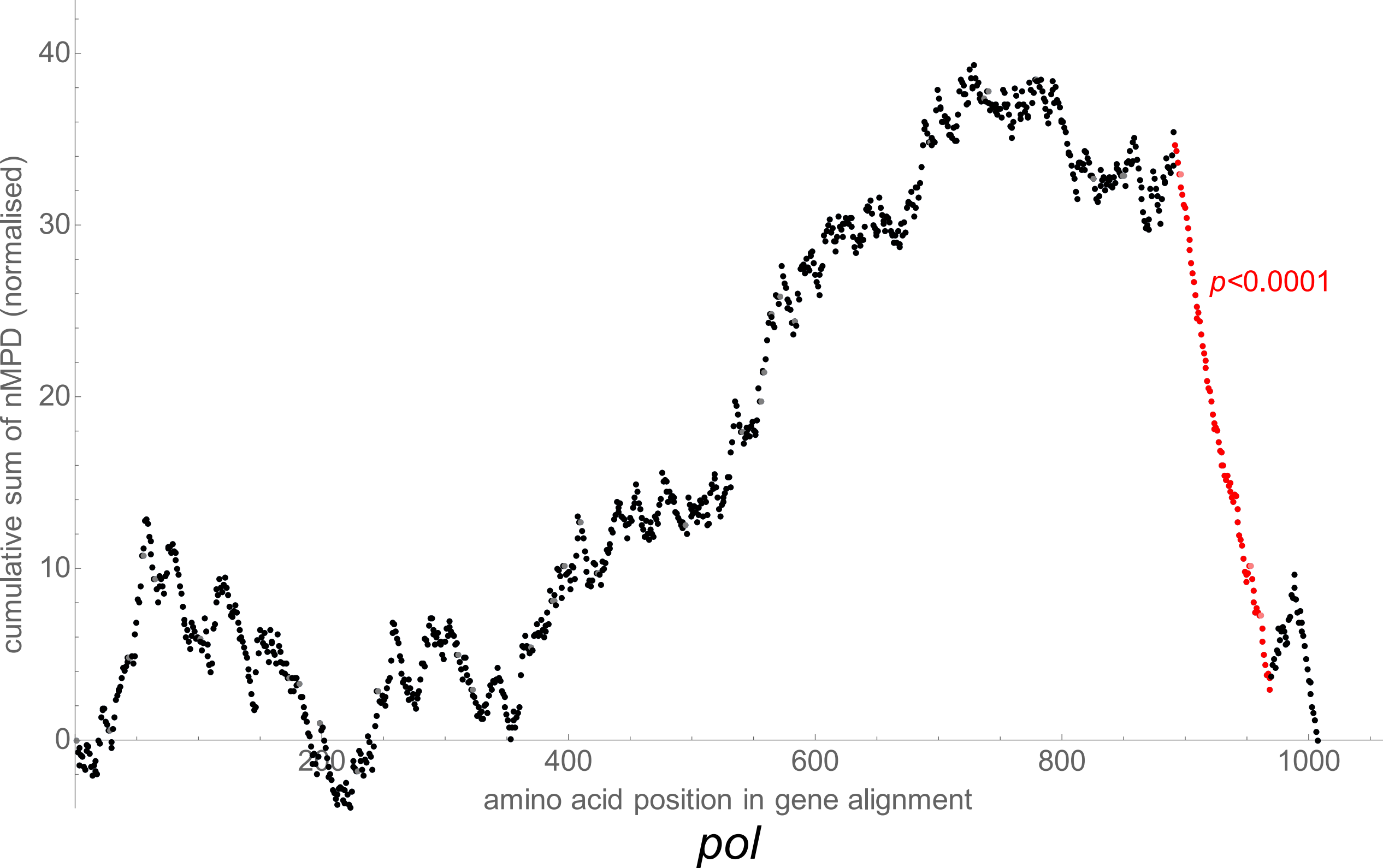

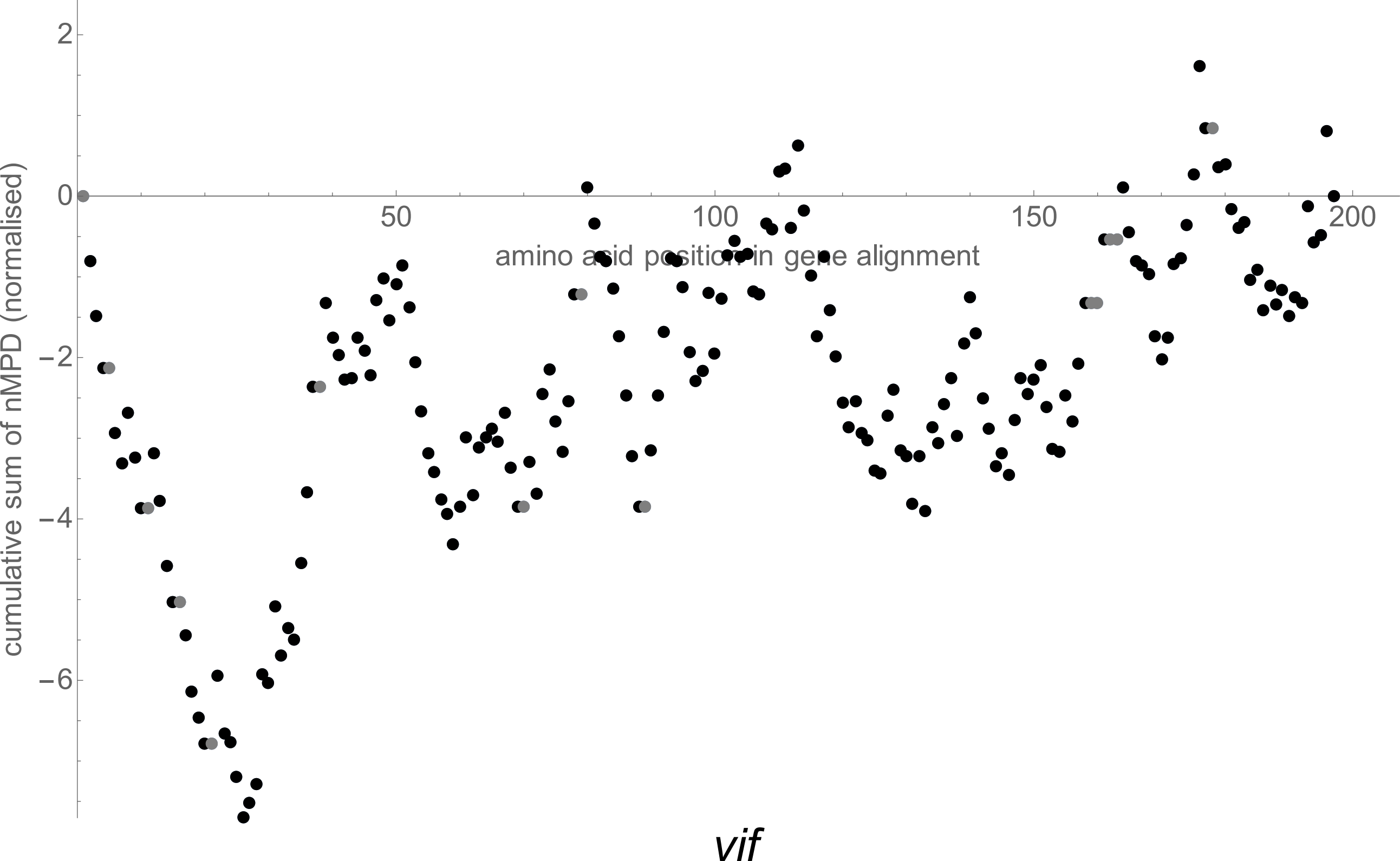

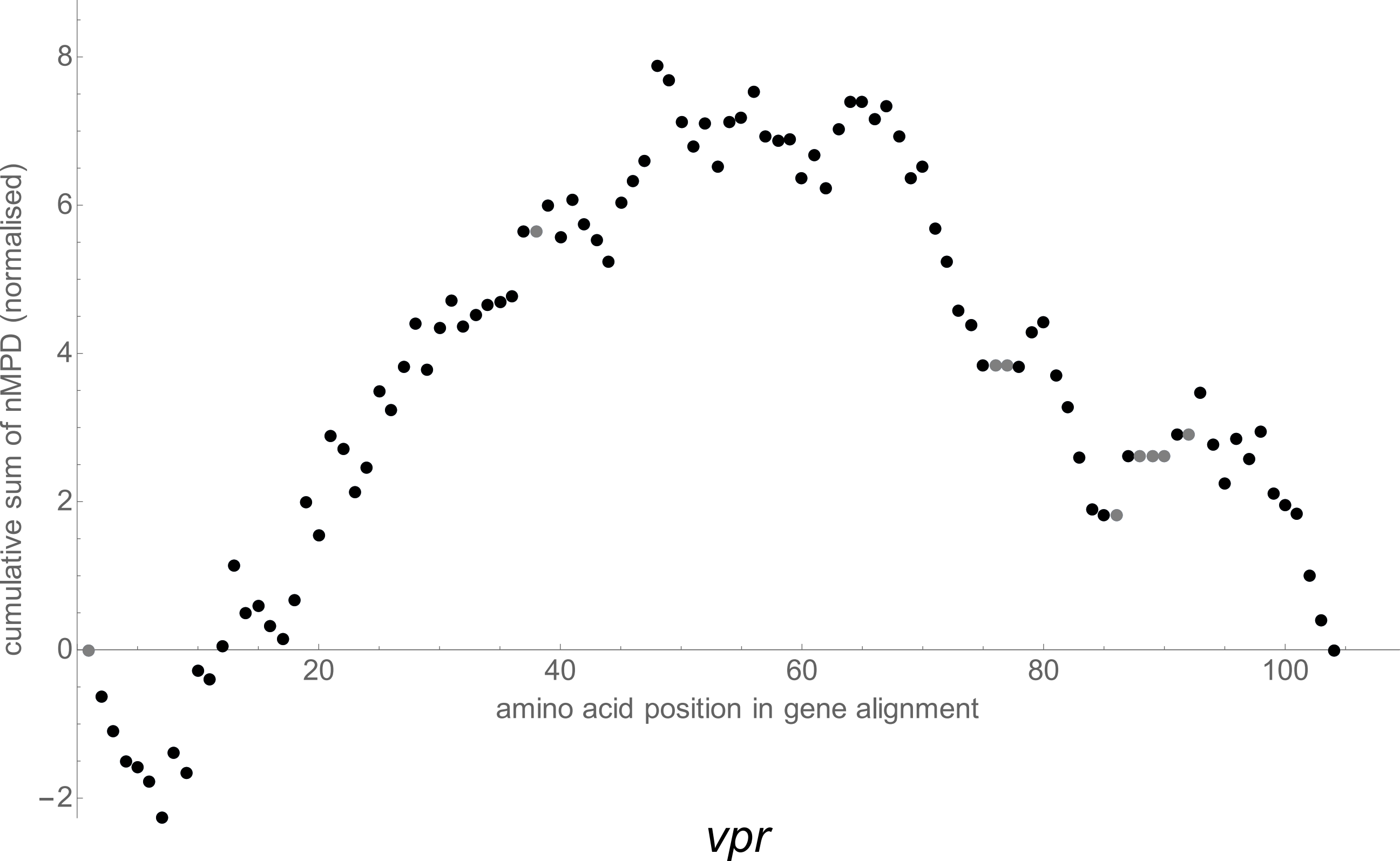

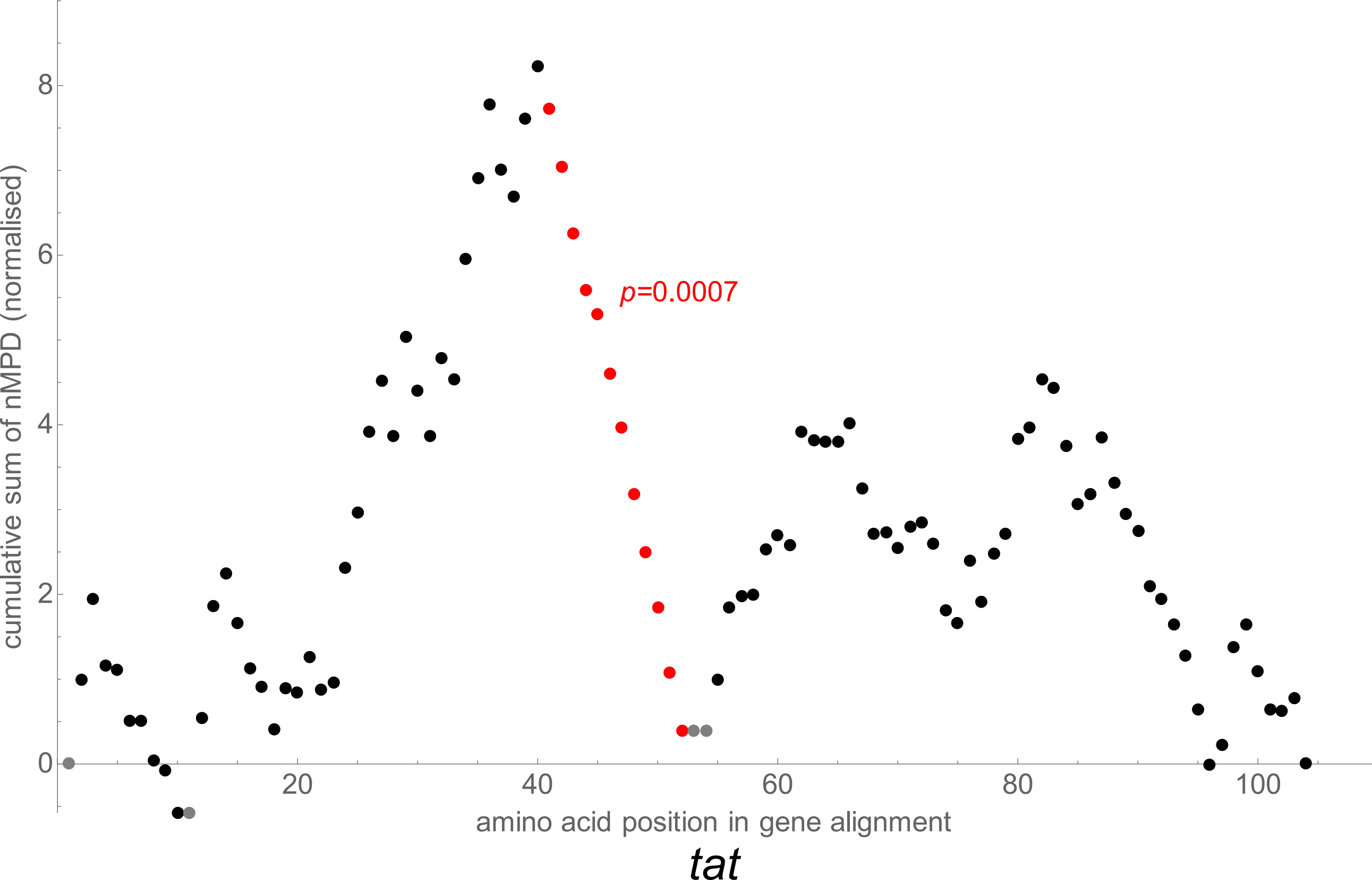

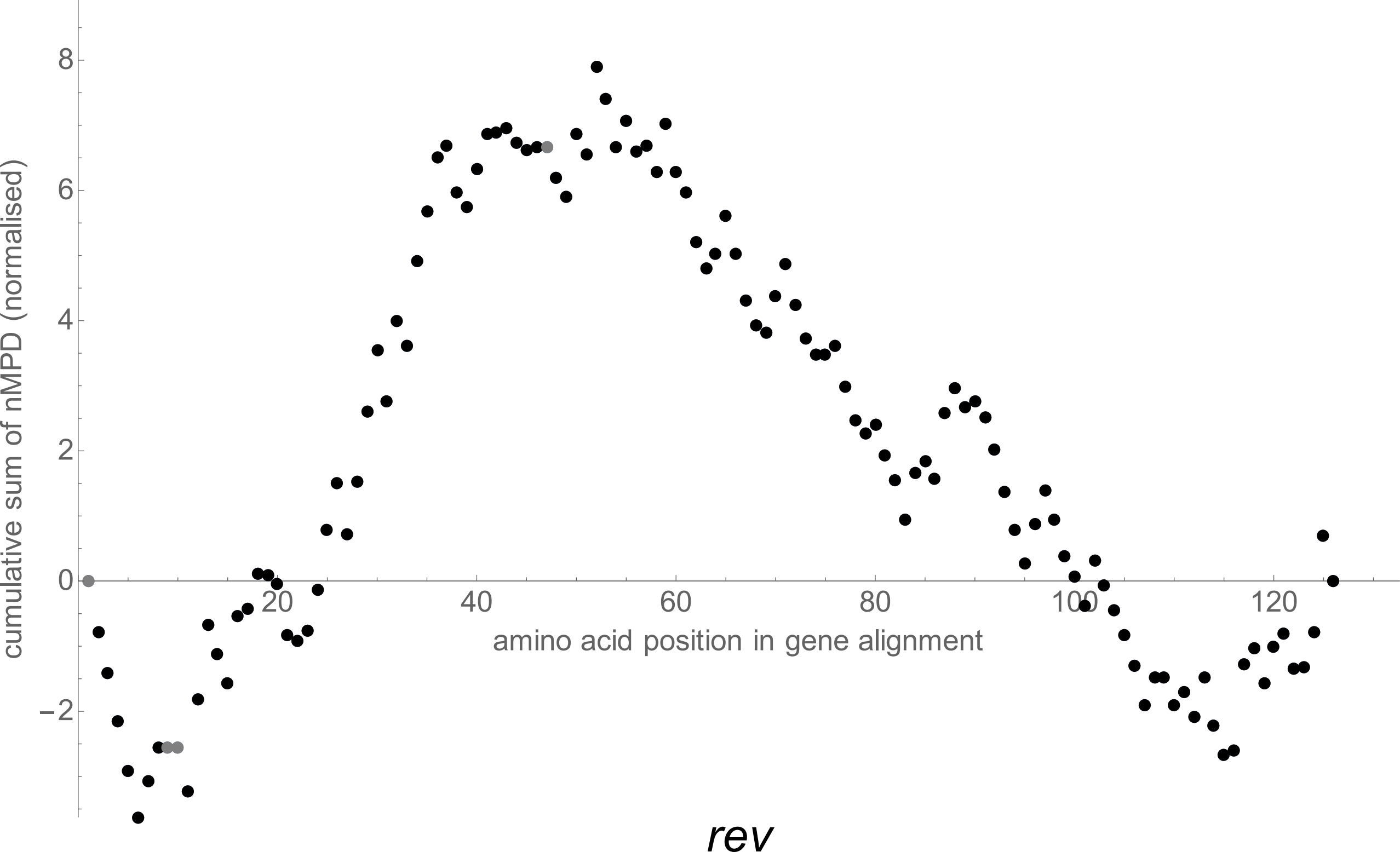

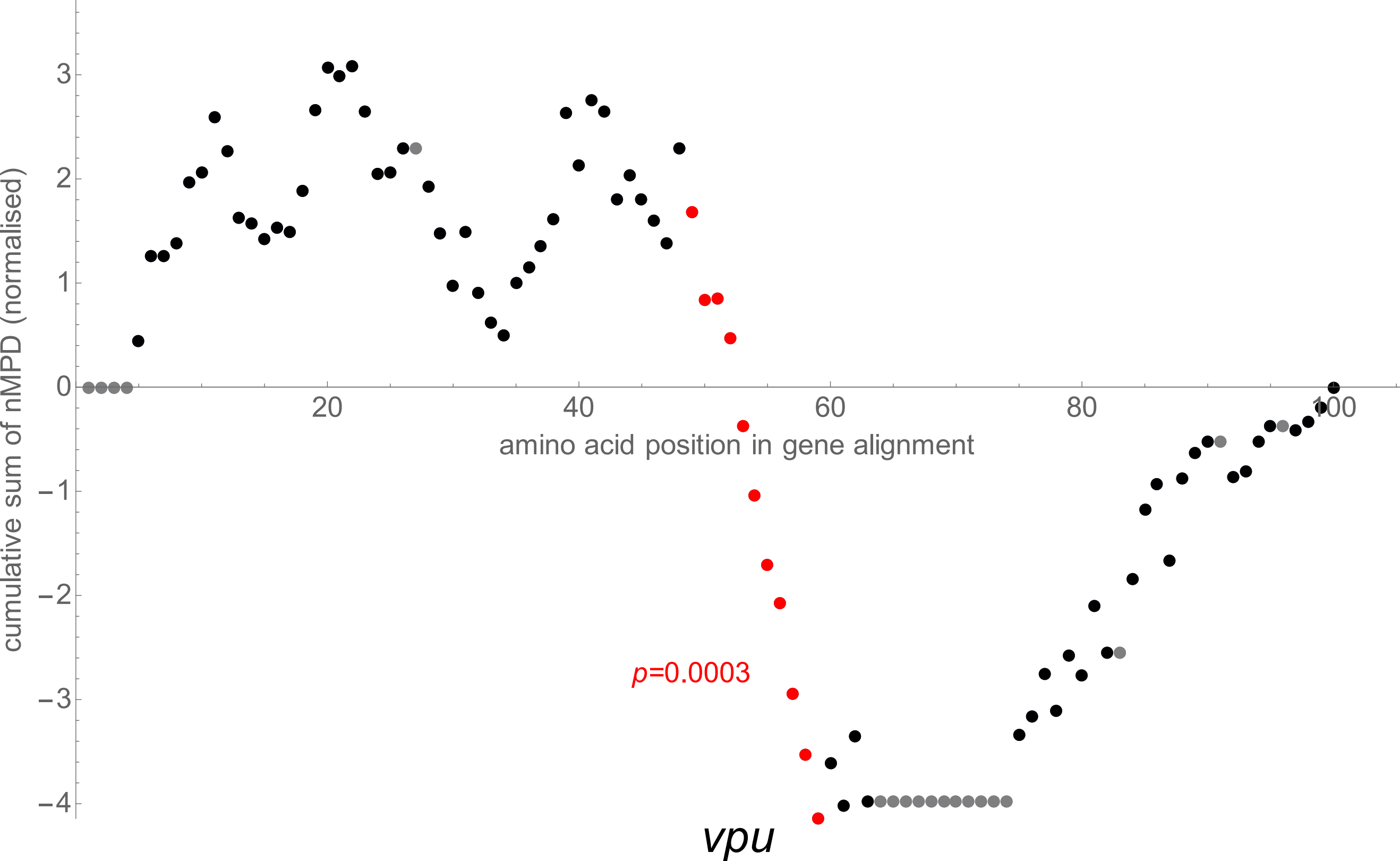

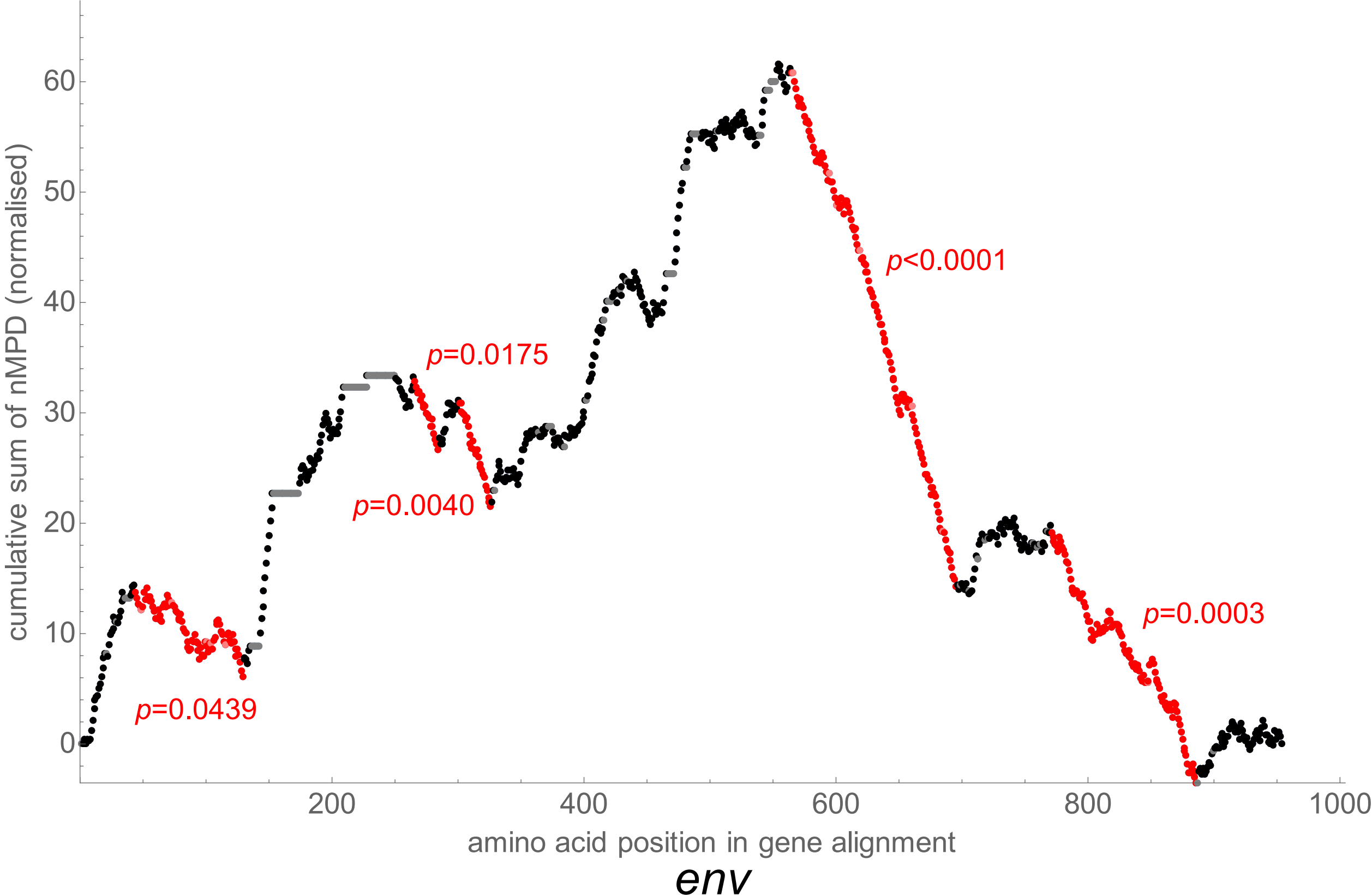

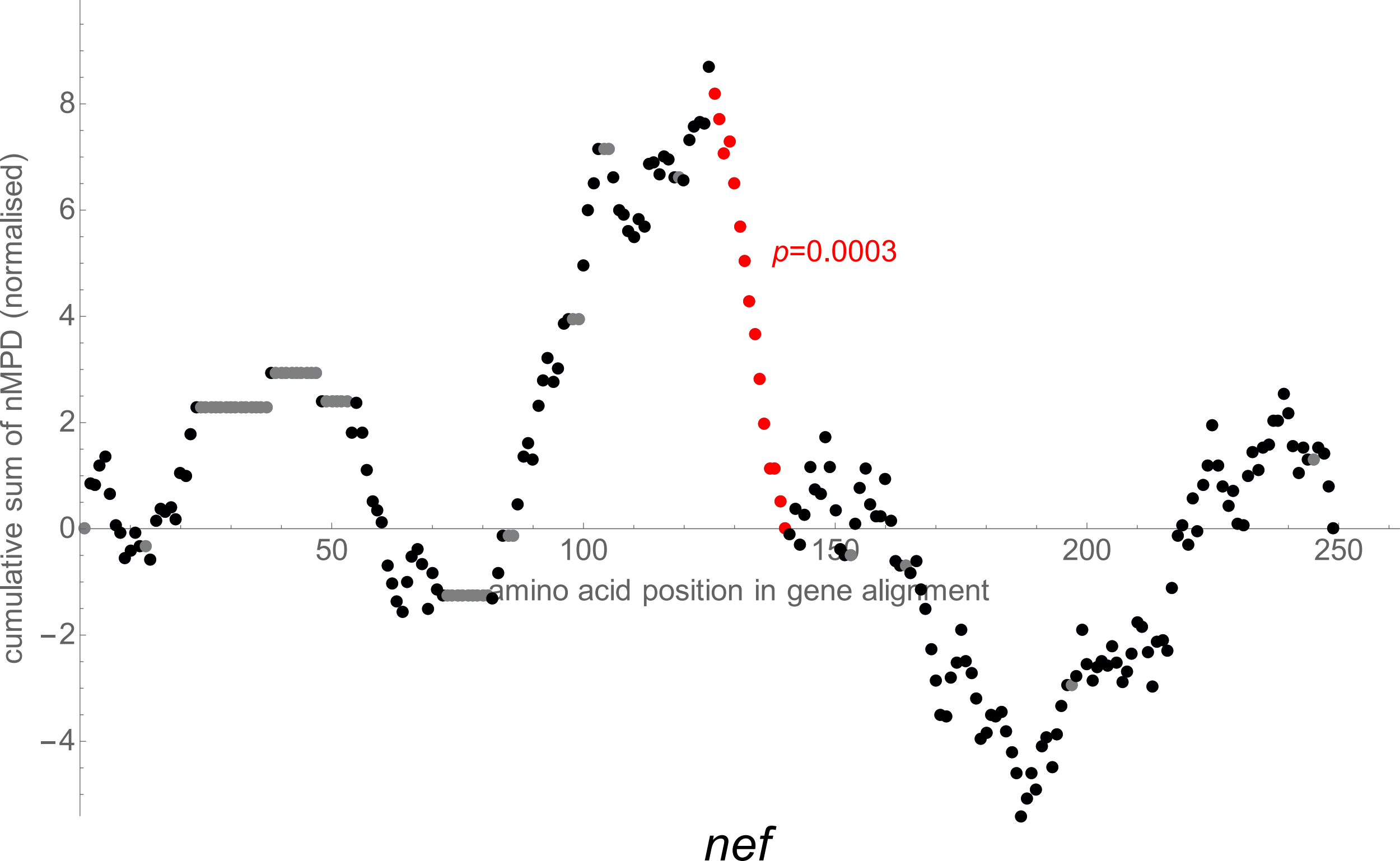

Supplement: S29 Fig — Shown in similar fashion to that of Fig 2. (PDF) [file pcbi.1007345.s029.pdf]

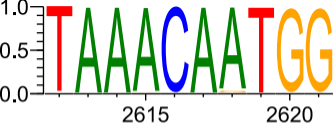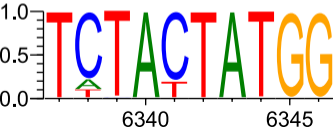

Supplement: S30 Fig — Shown in similar fashion to that of Fig 9. (PDF) [file pcbi.1007345.s030.pdf]

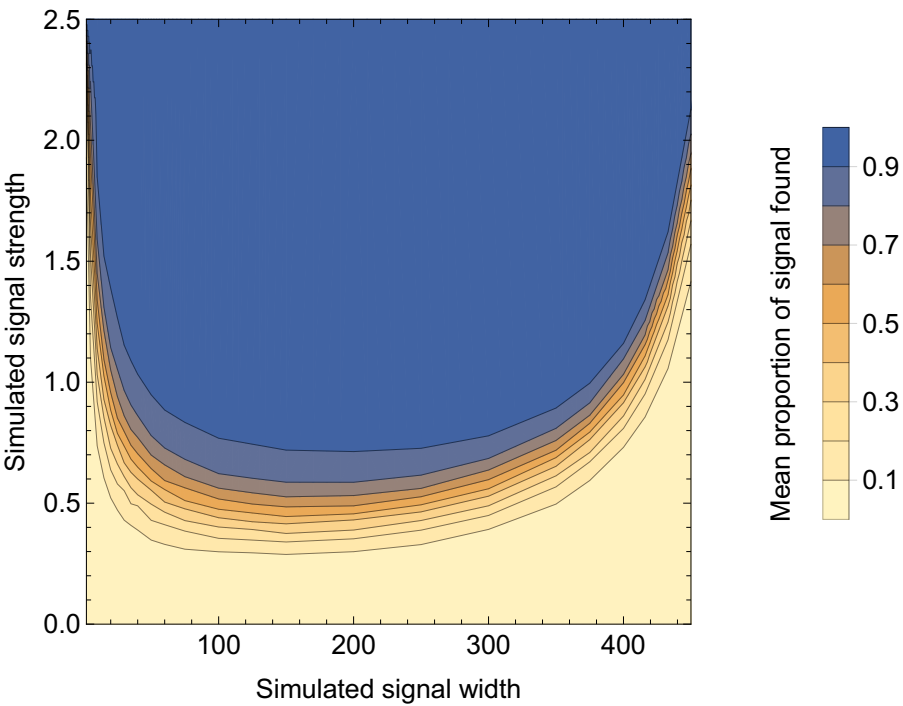

Supplement: S31 Fig — Replicating Fig 5 of the benchmarking work in [13], but here extending to include shorter signals. This shows that in the range of short signals (less than width 50), the shorter the signal, the stronger the strength of the signal must be for successful detection. Simulated data used for signal widths from 50 to 450 are identical to those used in the plot in [13]. (PDF) [file pcbi.1007345.s031.pdf]
