# Supplementary material for: A scale-free analysis of the HIV-1 genome demonstrates multiple conserved regions of structural and functional importance
Source: PLoS Comput Biol. 2019 Sep 23;15(9):e1007345. doi: 10.1371/journal.pcbi.1007345 (PMC6791557; doi:10.1371/journal.pcbi.1007345)
Supplement: S1 Table — (PDF) [file pcbi.1007345.s032.pdf]

AB074051	AB074053	AB074056	AB074058	AB074060	AB074066	AB074069	AB074071
AB078005	AB078703	AB097870	AB221005	AB221126	AB286956	AB287363	AB287364
AB287367	AB287368	AB287372	AB289588	AB289590	AB428551	AB428558	AB480692
AB480694	AB480696	AB480698	AB485640	AB564745	AB565478	AB565496	AB565497
AB565499	AB565502	AB604946	AB604948	AB641836	AF004394	AF042100	AF042101
AF042103	AF042104	AF042105	AF049494	AF069140	AF086817	AF128998	AF146728
AF224507	AF538302	AF538303	AF538304	AF538305	AF538306	AF538307	AJ271445
AJ437035	AJ437041	AJ437050	AJ437053	AY173951	AY173952	AY180905	AY206647
AY206651	AY206652	AY206653	AY206654	AY206657	AY206662	AY206664	AY275555
AY314063	AY331293	AY331296	AY332236	AY352275	AY560107	AY560108	AY560110
AY586542	AY586543	AY679786	AY751407	AY779552	AY779553	AY779559	AY786869
AY786878	AY786949	AY818644	AY835749	AY835753	AY835758	AY835761	AY835763
AY835768	AY835769	AY835773	AY835774	AY835775	AY835778	AY839827	AY970950
DQ097750	DQ127534	DQ295192	DQ295193	DQ295195	DQ322223	DQ322225	DQ358805
DQ358808	DQ358809	DQ358810	DQ396398	DQ833405	DQ833406	DQ833408	DQ833409
DQ833410	DQ833411	DQ833413	DQ833414	DQ833416	DQ833417	DQ833418	DQ833419
DQ833420	DQ833421	DQ833422	DQ833423	DQ833424	DQ833425	DQ833427	DQ833429
DQ833430	DQ833433	DQ833434	DQ833435	DQ833436	DQ837381	DQ853436	DQ854716
DQ886031	DQ886032	DQ886033	DQ886034	DQ886035	DQ886036	DQ886037	DQ990880
DQ996251	DQ996253	EF090290	EF119604	EF122510	EF122514	EF122519	EF122522
EF122529	EF122533	EF122538	EF122545	EF125633	EF175212	EF363123	EF363124
EF363126	EF363127	EF370299	EF370326	EF370327	EF370338	EF370365	EF370373
EF370375	EF394232	EF394233	EF394234	EF394235	EF514697	EF514698	EF514700
EF514704	EF514705	EF514707	EF514710	EF514711	EF514712	EF637046	EF637047
EF637048	EF637049	EF637050	EF637051	EF637053	EF637054	EF637056	EF637057
EF694037	EU100418	EU241958	EU242017	EU242026	EU242046	EU242103	EU242106
EU242122	EU242134	EU242178	EU242192	EU242197	EU242216	EU242222	EU242228
EU242248	EU242293	EU242364	EU242372	EU242380	EU242446	EU242447	EU242455
EU517768	EU517770	EU517778	EU517785	EU517791	EU517792	EU517797	EU517798
EU517799	EU517800	EU517808	EU517810	EU517811	EU517813	EU547186	EU616649
EU673412	EU673413	EU673417	EU673419	EU673422	EU673427	EU673430	EU673436
EU786672	EU786676	EU786678	EU786680	EU807832	EU807833	EU864062	FJ155120
FJ155186	FJ155192	FJ155199	FJ155200	FJ155209	FJ195086	FJ195088	FJ195089
FJ195090	FJ195091	FJ211646	FJ211777	FJ224363	FJ224364	FJ387525	FJ387526
FJ387531	FJ387533	FJ387534	FJ387535	FJ387536	FJ387541	FJ388895	FJ388898
FJ388899	FJ388904	FJ388905	FJ388910	FJ388911	FJ388914	FJ388915	FJ388916
FJ388919	FJ388924	FJ388931	FJ388933	FJ388936	FJ388957	FJ388958	FJ388959
FJ388960	FJ388962	FJ388963	FJ388964	FJ460501	FJ469683	FJ469684	FJ469686
FJ469687	FJ469689	FJ469691	FJ469692	FJ469696	FJ469697	FJ469698	FJ469699
FJ469700	FJ469701	FJ469703	FJ469704	FJ469705	FJ469707	FJ469708	FJ469710
FJ469711	FJ469713	FJ469714	FJ469715	FJ469716	FJ469717	FJ469718	FJ469719
FJ469720	FJ469721	FJ469722	FJ469723	FJ469725	FJ469726	FJ469727	FJ469729
FJ469730	FJ469731	FJ469732	FJ469734	FJ469735	FJ469737	FJ469738	FJ469739
FJ469740	FJ469741	FJ469743	FJ469744	FJ469745	FJ469747	FJ469748	FJ469749
FJ469752	FJ469753	FJ469755	FJ469758	FJ469759	FJ469760	FJ469761	FJ469763
FJ469767	FJ469768	FJ469769	FJ469770	FJ469771	FJ469772	FJ495818	FJ495941
FJ496000	FJ496078	FJ496081	FJ496145	FJ496151	FJ596180	FJ649603	FJ649604
FJ649605	FJ649606	FJ649607	FJ667208	FJ667209	FJ667212	FJ667213	FJ667214
FJ667217	FJ667218	FJ667223	FJ667229	FJ667230	FJ667232	FJ667233	FJ667234
FJ667235	FJ667237	FJ667239	FJ667244	FJ667246	FJ667247	FJ667248	FJ667250
FJ667252	FJ667255	FJ670525	FJ670531	FJ694790	FJ853620	FJ853622	GQ256628
GQ371219	GQ371224	GQ371245	GQ371249	GQ371250	GQ371275	GQ371281	GQ371286
GQ371287	GQ371290	GQ371314	GQ371341	GQ371379	GQ371402	GQ371418	GQ371425
GQ371435	GQ371453	GQ371463	GQ371493	GQ371552	GQ371561	GQ371579	GQ371583
GQ371593	GQ371598	GQ371601	GQ371605	GQ371606	GQ371615	GQ371640	GQ371641
GQ371674	GQ371696	GQ371703	GQ371712	GQ372988	GQ372990	GU177863	GU331318
GU362881	GU362883	GU362885	GU367413	GU367416	GU367418	GU367421	GU367427

GU367429	GU367434	GU367438	GU367443	GU390465	GU390467	GU390468	GU390469
GU390470	GU390472	GU390473	GU390475	GU390476	GU390481	GU390482	GU390484
GU390486	GU390488	GU390489	GU390492	GU390497	GU390499	GU390502	GU390504
GU390507	GU390510	GU390513	GU390514	GU390515	GU390528	GU561129	GU561134
GU561140	GU561146	GU561157	GU561180	GU561192	GU561195	GU561197	GU561203
GU561207	GU561215	GU561220	GU561238	GU561275	GU561286	GU561300	GU733713
HM116827	HM586187	HM586193	HM586210	HQ197984	HQ197985	HQ197986	HQ197988
HQ215554	HQ846902	JF304757	JF304758	JF304759	JF320003	JF320008	JF320013
JF320028	JF320036	JF320038	JF320043	JF320048	JF320053	JF320054	JF320059
JF320097	JF320101	JF320126	JF320130	JF320145	JF320150	JF320151	JF320159
JF320160	JF320169	JF320174	JF320182	JF320183	JF320185	JF320189	JF320197
JF320208	JF320215	JF320228	JF320244	JF320263	JF320277	JF320316	JF320347
JF320349	JF320356	JF320361	JF320363	JF320381	JF320386	JF320413	JF320424
JF320427	JF320437	JF320467	JF320484	JF320493	JF320526	JF320530	JF320563
JF320564	JF320577	JF320594	JF320613	JF320615	JF320634	JF683738	JF683742
JF683743	JF683747	JF683750	JF683751	JF683753	JF683764	JF683765	JF683769
JF683773	JF683775	JF683778	JF683781	JF683784	JF683787	JF683790	JF683794
JF683796	JF683797	JF683801	JF683805	JF683807	JF683809	JF932468	JF932469
JF932470	JF932471	JF932472	JF932473	JF932474	JF932475	JF932476	JF932477
JF932478	JF932479	JF932480	JF932481	JF932482	JF932483	JF932484	JF932485
JF932486	JF932487	JF932488	JF932489	JF932490	JF932491	JF932492	JF932493
JF932494	JF932495	JF932496	JF932497	JF932498	JF932499	JF932500	JN024100
JN024210	JN024303	JN024344	JN024363	JN024428	JN248321	JN248329	JN248333
JN248335	JN248337	JN248346	JN248347	JN248354	JN400483	JN408075	JN408076
JN408077	JN613771	JN687657	JN687665	JN687675	JN687677	JN687678	JN687690
JN687691	JN692432	JN692433	JN692435	JN692439	JN692440	JN692443	JN692444
JN692445	JN692446	JN692450	JN692451	JN692452	JN692453	JN692454	JN692455
JN692457	JN692460	JN692461	JN692462	JN692463	JN692465	JN692467	JN692468
JN692471	JN692473	JN692474	JN692475	JN692479	JN692480	JN704057	JN944897
JN944905	JN944907	JN944909	JN944911	JN944917	JN944928	JN944930	JN944936
JN944938	JQ028195	JQ316126	JQ316127	JQ316128	JQ316129	JQ316130	JQ316131
JQ316132	JQ316133	JQ316134	JQ316135	JQ341411	JQ403019	JQ403022	JQ403023
JQ403024	JQ403025	JQ403026	JQ403031	JQ403035	JQ403037	JQ403042	JQ403045
JQ403056	JQ403057	JQ403059	JQ403060	JQ403061	JQ403062	JQ403064	JQ403065
JQ403066	JQ403067	JQ403068	JQ403069	JQ403070	JQ403071	JQ403072	JQ403074
JQ403076	JQ403078	JQ403079	JQ403081	JQ403082	JQ403083	JQ403085	JQ403086
JQ403087	JQ403088	JQ403091	JQ403092	JQ403093	JQ403094	JQ403095	JQ403096
JQ403097	JQ403098	JQ403100	JQ403102	JQ403104	JQ403107	JQ416158	JQ429433
JQ647933	JQ716362	JQ846091	JQ846093	JQ846094	JQ846096	JQ846097	JQ846098
JQ846099	JQ846100	JQ846101	JQ846102	JQ846103	JQ846104	JQ846105	JQ846106
JQ846107	JQ846108	JQ846109	JQ846110	JQ846111	JQ846112	JQ846113	JQ846115
JQ846116	JQ846119	JQ846121	JQ846122	JQ846123	JQ846124	JQ846126	JQ846127
JQ846128	JQ846129	JQ846130	JQ846131	JQ846132	JQ846133	JQ846136	JQ846150
JQ846151	JQ846152	JQ846153	JQ846159	JQ846161	JQ846162	JQ846165	JQ846167
JQ846168	JQ846169	JQ846172	JQ846175	JQ846177	JQ846178	JQ846179	JQ846180
JQ846182	JQ846184	JQ846187	JQ846191	JQ846201	JQ846203	JQ846208	JQ846209
JQ846210	JQ846211	JQ846212	JQ846213	JQ846214	JQ846221	JQ846222	JQ846225
JQ846229	JQ846235	JQ846237	JQ846238	JQ846239	JQ900850	JQ900861	JQ900862
JQ900885	JQ900914	JQ900924	JX140652	JX140654	JX140657	JX140658	JX140659
JX264247	JX264249	JX264251	JX264252	JX264265	JX264268	JX264289	JX264298
JX264300	JX264307	JX264322	JX264331	JX264332	JX264354	JX264355	JX264356
JX264358	JX264363	JX264374	JX264379	JX264385	JX264386	JX264387	JX264408
JX264410	JX446800	JX446807	JX447156	JX448096	JX500707	JX500708	JX500709
JX503071	JX503075	JX960597	JX960598	JX960599	K03455	KJ140264	KJ140266
L02317	M17449	M17451	M26727	M38429	M38431	U21135	U23487
U39362	U43141	U71182					
