# Supplementary material for: A scale-free analysis of the HIV-1 genome demonstrates multiple conserved regions of structural and functional importance
Source: PLoS Comput Biol. 2019 Sep 23;15(9):e1007345. doi: 10.1371/journal.pcbi.1007345 (PMC6791557; doi:10.1371/journal.pcbi.1007345)
Supplement: S2 Table — (PDF) [file pcbi.1007345.s033.pdf]

AB078005	AB097870	AB221005	AB221126	AB286956	AB287363	AB287364	AB287367
AB287368	AB289588	AB289590	AB428551	AB480692	AB480694	AB480696	AB480698
AB564745	AB564746	AB565478	AB565496	AB565497	AB565499	AB565502	AB604946
AB604948	AF004394	AF042102	AF042103	AF042104	AF042105	AF069140	AF146728
AF156844	AF156850	AF286365	AF538302	AF538303	AF538306	AF538307	AJ271445
AY037268	AY037269	AY037270	AY037282	AY173951	AY173952	AY173953	AY173959
AY173960	AY180905	AY247251	AY314061	AY331282	AY331284	AY331289	AY331294
AY331296	AY332237	AY560107	AY560108	AY560109	AY560110	AY561236	AY561237
AY561239	AY561244	AY586542	AY713410	AY779550	AY779553	AY779557	AY781126
AY781127	AY795904	AY795905	AY818644	AY835749	AY835753	AY835758	AY835761
AY835763	AY835768	AY835769	AY835773	AY835775	AY835778	AY839827	AY945710
AY970946	D10112	DQ127534	DQ127537	DQ127542	DQ127548	DQ207942	DQ207943
DQ295193	DQ354118	DQ354119	DQ358805	DQ358808	DQ358809	DQ358810	DQ383748
DQ383749	DQ396398	DQ487188	DQ487190	DQ823362	DQ823364	DQ837381	DQ853436
DQ853463	DQ854716	DQ886031	DQ886032	DQ886033	DQ886034	DQ886035	DQ886036
DQ886037	DQ990880	EF175212	EF363123	EF363126	EF363127	EF514697	EF514698
EF514700	EF514704	EF514705	EF514710	EF637046	EF637047	EF637048	EF637049
EF637050	EF637051	EF637053	EF637054	EF637056	EF637057	EF694037	EU547186
EU616649	EU786678	EU786680	EU807784	EU807790	FJ195086	FJ195088	FJ195089
FJ195090	FJ195091	FJ388890	FJ388895	FJ388899	FJ388904	FJ388905	FJ388911
FJ388914	FJ388915	FJ388919	FJ388930	FJ388931	FJ388933	FJ388934	FJ388935
FJ388940	FJ388947	FJ388949	FJ388956	FJ388957	FJ388958	FJ388959	FJ388960
FJ388964	FJ388965	FJ403482	FJ469682	FJ469683	FJ469684	FJ469685	FJ469686
FJ469687	FJ469688	FJ469689	FJ469690	FJ469691	FJ469692	FJ469693	FJ469694
FJ469695	FJ469696	FJ469697	FJ469698	FJ469699	FJ469700	FJ469701	FJ469702
FJ469703	FJ469704	FJ469705	FJ469707	FJ469708	FJ469709	FJ469710	FJ469711
FJ469712	FJ469713	FJ469714	FJ469715	FJ469717	FJ469718	FJ469719	FJ469721
FJ469722	FJ469723	FJ469725	FJ469726	FJ469727	FJ469729	FJ469730	FJ469731
FJ469732	FJ469734	FJ469735	FJ469737	FJ469738	FJ469739	FJ469740	FJ469741
FJ469742	FJ469743	FJ469744	FJ469745	FJ469747	FJ469748	FJ469749	FJ469750
FJ469751	FJ469752	FJ469753	FJ469755	FJ469756	FJ469757	FJ469758	FJ469759
FJ469760	FJ469761	FJ469763	FJ469764	FJ469766	FJ469767	FJ469768	FJ469769
FJ469770	FJ469771	FJ469772	FJ495818	FJ496000	FJ496081	FJ496151	FJ670531
FJ694790	GQ371772	GQ371798	GQ371839	GQ371929	GQ371967	GQ372103	GQ372141
GQ372153	GQ372173	GQ372194	GQ372988	GU362881	GU362883	GU362886	GU367462
GU367465	GU367466	GU367467	GU367472	GU367474	GU367484	GU733713	HM030560
HM030561	HM030562	HM030565	HM586187	HM586193	HM586198	HQ026500	HQ026501
HQ026502	HQ026506	HQ026507	HQ026514	HQ026517	HQ026523	HQ026530	HQ026539
HQ026556	HQ026571	HQ026573	HQ026605	HQ026612	HQ026615	HQ026619	HQ026624
HQ026628	HQ026630	HQ026632	K02007	K03455	L02317	M17451	M26727
M38429	M38431	U21135	U23487	U34604	U39362	U43096	U43141
U71182							
