# Supplementary material for: A scale-free analysis of the HIV-1 genome demonstrates multiple conserved regions of structural and functional importance
Source: PLoS Comput Biol. 2019 Sep 23;15(9):e1007345. doi: 10.1371/journal.pcbi.1007345 (PMC6791557; doi:10.1371/journal.pcbi.1007345)
Supplement: S3 Table — (PDF) [file pcbi.1007345.s034.pdf]

AB034456	AB034460	AB034467	AB034469	AB034474	AB034479	AB034486	AB034489
AB034494	AB034499	AB034503	AB097870	AB221126	AB287363	AB287364	AB287367
AB287368	AB287372	AB289588	AB289590	AB428551	AB428558	AB480692	AB480696
AB480698	AB564745	AB564746	AB565478	AB565496	AB565497	AB565499	AB565502
AB604946	AB604948	AB641836	AB731663	AB731665	AB731667	AB731669	AF003887
AF004394	AF042101	AF042102	AF042103	AF042104	AF042105	AF049495	AF069140
AF086817	AF143097	AF143098	AF143099	AF143100	AF143101	AF143103	AF143104
AF143105	AF143106	AF143107	AF143108	AF143111	AF143115	AF143116	AF143119
AF143120	AF143121	AF143123	AF143125	AF143126	AF143130	AF143132	AF143134
AF143135	AF143136	AF143137	AF143138	AF143144	AF146728	AF224507	AF286365
AF538302	AF538303	AF538304	AF538305	AF538306	AF538307	AJ271445	AJ437540
AJ437542	AJ437545	AJ437551	AJ437554	AY005992	AY037268	AY037269	AY037270
AY037282	AY064706	AY173951	AY173952	AY173955	AY173956	AY173959	AY173960
AY180905	AY247251	AY314061	AY331282	AY331284	AY331287	AY331289	AY331292
AY331294	AY331296	AY332237	AY352275	AY423381	AY560107	AY560109	AY560110
AY561236	AY561237	AY561238	AY561239	AY561244	AY581328	AY581359	AY581364
AY581368	AY581369	AY581376	AY581404	AY581419	AY581421	AY586542	AY586543
AY713410	AY751407	AY779553	AY779557	AY781126	AY781127	AY795904	AY795905
AY818644	AY835749	AY835753	AY835758	AY835763	AY835768	AY835773	AY835774
AY835775	AY835778	AY835779	AY835781	AY839827	AY856961	AY857022	AY857127
AY945710	AY970946	D10112	D70858	D70867	D70874	D70887	DQ072736
DQ072738	DQ072756	DQ072766	DQ072778	DQ072784	DQ072793	DQ072796	DQ072828
DQ072835	DQ072849	DQ097754	DQ097760	DQ097763	DQ097765	DQ097767	DQ127534
DQ127537	DQ127542	DQ127548	DQ154014	DQ154022	DQ154026	DQ154028	DQ154031
DQ154032	DQ207940	DQ207942	DQ354112	DQ354119	DQ358808	DQ358809	DQ358810
DQ383746	DQ383748	DQ383749	DQ383750	DQ383751	DQ383752	DQ396398	DQ487188
DQ672623	DQ676875	DQ676886	DQ823362	DQ823363	DQ823364	DQ853463	DQ871530
DQ871540	DQ871541	DQ871542	DQ871546	DQ871564	DQ871567	DQ871568	DQ871570
DQ871572	DQ871573	DQ871575	DQ871577	DQ871578	DQ871579	DQ871580	DQ871581
DQ871582	DQ871585	DQ871586	DQ871587	DQ871589	DQ886031	DQ886032	DQ886033
DQ886034	DQ886035	DQ886036	DQ886037	DQ990880	EF125639	EF175212	EF178358
EF363123	EF363126	EF363127	EF514697	EF514698	EF514699	EF514700	EF514701
EF514704	EF514705	EF514707	EF514708	EF514709	EF514710	EF514711	EF637046
EF637047	EF637048	EF637049	EF637050	EF637051	EF637053	EF637054	EF637056
EF637057	EF694037	EU384245	EU518049	EU518051	EU518059	EU518064	EU518076
EU518077	EU518078	EU518079	EU518086	EU518087	EU547186	EU616649	EU786678
EU786679	EU839596	EU839597	EU839598	EU839600	EU839601	EU839602	EU839604
EU839605	EU839606	EU839607	EU839608	EU839609	FJ195086	FJ195088	FJ195089
FJ195090	FJ195091	FJ197321	FJ197330	FJ388890	FJ388895	FJ388898	FJ388899
FJ388904	FJ388910	FJ388911	FJ388912	FJ388927	FJ388930	FJ388931	FJ388933
FJ388934	FJ388935	FJ388936	FJ388937	FJ388940	FJ388941	FJ388949	FJ388955
FJ388956	FJ388958	FJ388959	FJ388964	FJ388965	FJ403482	FJ469684	FJ469685
FJ469686	FJ469687	FJ469688	FJ469689	FJ469690	FJ469691	FJ469692	FJ469693
FJ469694	FJ469695	FJ469696	FJ469697	FJ469698	FJ469699	FJ469700	FJ469701
FJ469702	FJ469703	FJ469704	FJ469705	FJ469707	FJ469708	FJ469710	FJ469711
FJ469712	FJ469713	FJ469714	FJ469715	FJ469716	FJ469717	FJ469718	FJ469719
FJ469720	FJ469721	FJ469722	FJ469723	FJ469725	FJ469726	FJ469727	FJ469729
FJ469730	FJ469731	FJ469732	FJ469734	FJ469735	FJ469737	FJ469738	FJ469739
FJ469740	FJ469741	FJ469742	FJ469743	FJ469744	FJ469747	FJ469748	FJ469750
FJ469751	FJ469752	FJ469753	FJ469755	FJ469756	FJ469757	FJ469758	FJ469759
FJ469760	FJ469761	FJ469763	FJ469764	FJ469766	FJ469767	FJ469768	FJ469769
FJ469770	FJ469771	FJ495818	FJ495941	FJ496000	FJ496078	FJ496081	FJ496145
FJ496151	FJ496169	FJ647145	FJ670525	FJ670531	FJ694790	FJ853622	FJ861953
GQ256639	GQ358531	GQ372988	GQ386779	GU177863	GU331147	GU331247	GU362886
GU367526	GU367529	GU367530	GU367532	GU367537	GU367538	GU367539	GU367540
GU367542	GU367544	GU367546	GU562001	GU562033	GU562058	GU562080	GU562135
GU562155	GU562236	GU562266	GU562272	GU729493	GU729498	GU729503	GU729509

GU729517	GU729556	GU729580	GU729599	GU729608	GU729665	GU729675	GU729681
GU729686	GU729687	GU729694	GU729697	GU729743	GU729754	GU729769	GU729778
GU729790	GU729794	GU729797	GU729807	GU729810	GU729815	GU729822	GU729828
GU729841	GU729843	GU729852	GU729854	GU729859	GU729862	GU729863	GU729866
GU729869	GU729871	GU729885	GU729898	GU729902	GU729905	GU729909	GU729918
GU729930	GU729935	GU729936	GU729937	GU729938	GU729950	GU729957	GU729963
GU729965	GU729970	GU729972	GU729974	GU729976	GU729982	GU729984	GU729989
GU729993	GU730004	GU730009	GU730010	GU730012	GU730023	GU730024	GU733713
HM030559	HM030560	HM030561	HM030562	HM030564	HM030565	HM586187	HM586193
HM586198	HM586210	HM586706	HQ846914	JF320003	JF320008	JF320013	JF320018
JF320028	JF320036	JF320038	JF320043	JF320045	JF320048	JF320053	JF320054
JF320059	JF320097	JF320101	JF320126	JF320130	JF320145	JF320150	JF320159
JF320160	JF320169	JF320174	JF320183	JF320184	JF320185	JF320189	JF320191
JF320197	JF320208	JF320215	JF320226	JF320228	JF320244	JF320263	JF320277
JF320307	JF320316	JF320347	JF320349	JF320356	JF320361	JF320363	JF320381
JF320386	JF320400	JF320413	JF320424	JF320427	JF320437	JF320467	JF320484
JF320493	JF320526	JF320530	JF320537	JF320563	JF320564	JF320577	JF320594
JF320613	JF320615	JF320634	JF494999	JF495000	JF495002	JF495003	JF495004
JF495005	JF495007	JF495008	JF495009	JF683736	JF683738	JF683741	JF683742
JF683743	JF683747	JF683749	JF683750	JF683751	JF683753	JF683754	JF683756
JF683764	JF683765	JF683769	JF683773	JF683775	JF683778	JF683781	JF683784
JF683785	JF683787	JF683788	JF683790	JF683793	JF683794	JF683796	JF683797
JF683801	JF683804	JF683805	JF683807	JF689852	JF689854	JF689856	JF689857
JF689859	JF689862	JF689863	JF689865	JF689866	JF689867	JF689870	JF689871
JF689872	JF689873	JF689875	JF689877	JF689879	JF689883	JF689886	JF689889
JF689890	JF689892	JF689893	JF689895	JF689896	JF932468	JF932469	JF932470
JF932471	JF932472	JF932473	JF932474	JF932475	JF932476	JF932477	JF932478
JF932479	JF932480	JF932481	JF932482	JF932483	JF932484	JF932485	JF932486
JF932487	JF932488	JF932489	JF932490	JF932491	JF932492	JF932493	JF932494
JF932495	JF932496	JF932497	JF932498	JF932499	JF932500	JF957868	JF957872
JF957873	JF957874	JF957881	JF957887	JF957888	JF957891	JF957972	JF957982
JF957983	JF957986	JF957991	JF957992	JF957993	JF957995	JF957998	JF957999
JF958001	JF958002	JF958003	JF958004	JF958006	JF958007	JF958008	JF958009
JF958011	JF958076	JN024100	JN024210	JN024303	JN024344	JN024363	JN024428
JN235958	JN235959	JN248321	JN248329	JN248333	JN248337	JN248343	JN248344
JN248346	JN248347	JN248353	JN248354	JN251901	JN397362	JN397364	JN397365
JN400469	JN599165	JN687675	JN687677	JN687678	JN687679	JN687690	JN687691
JN687749	JN692432	JN692433	JN692435	JN692439	JN692440	JN692443	JN692444
JN692445	JN692450	JN692451	JN692452	JN692453	JN692454	JN692455	JN692457
JN692459	JN692460	JN692461	JN692462	JN692463	JN692465	JN692467	JN692468
JN692470	JN692471	JN692473	JN692474	JN692475	JN692479	JN692480	JN860769
JN944897	JN944905	JN944907	JN944909	JN944911	JN944917	JN944928	JN944930
JN944936	JN944938	JQ067011	JQ067069	JQ067077	JQ067080	JQ248203	JQ248237
JQ248336	JQ248545	JQ268970	JQ269106	JQ269178	JQ269187	JQ269218	JQ269222
JQ316126	JQ316127	JQ316128	JQ316129	JQ316130	JQ316131	JQ316132	JQ316133
JQ316134	JQ316135	JQ327767	JQ341411	JQ403019	JQ403020	JQ403021	JQ403022
JQ403023	JQ403024	JQ403025	JQ403026	JQ403029	JQ403031	JQ403035	JQ403037
JQ403042	JQ403043	JQ403044	JQ403045	JQ403046	JQ403047	JQ403048	JQ403058
JQ403059	JQ403060	JQ403061	JQ403062	JQ403063	JQ403064	JQ403065	JQ403066
JQ403067	JQ403068	JQ403069	JQ403070	JQ403071	JQ403073	JQ403074	JQ403075
JQ403077	JQ403078	JQ403079	JQ403080	JQ403081	JQ403082	JQ403083	JQ403084
JQ403085	JQ403086	JQ403087	JQ403089	JQ403091	JQ403092	JQ403093	JQ403094
JQ403095	JQ403096	JQ403097	JQ403098	JQ403100	JQ403102	JQ403103	JQ403104
JQ403105	JQ403106	JQ403107	JQ409028	JQ416158	JQ429433	JX140652	JX140654
JX140656	JX140657	JX140658	JX140659	JX446800	JX446807	JX447156	JX447790
JX448096	JX500707	JX500708	JX500709	JX503075	JX960597	JX960598	JX960599
JX972342	JX974238	JX999596	JX999597	K02007	K03455	KC312332	KC312435

KC312510	KC312583	KC473824	KC473825	KC473826	KC473827	KC473828	KC473829
KC473830	KC473831	KC473832	KC473833	KC473834	KC473835	KC473841	KC473842
KC473846	KC596066	KC596067	KC596069	KC797171	KC797225	KC899011	KC935957
KC935958	KF270358	KF270396	KF270399	KF270429	KF270437	KF301724	KF301759
KF301786	KF301791	KF384798	KF384799	KF384800	KF384801	KF384802	KF384803
KF384804	KF384805	KF384806	KF384807	KF384808	KF384810	KF384811	KF384812
KF384813	KF384814	KF526141	KF526228	KF526265	KF526312	KF526323	KF561441
KF561442	KF716494	KF716495	KF716496	KF716497	KF716498	KF990605	KF990608
KJ019215	KJ140249	KJ140250	KJ140251	KJ140255	KJ140256	KJ140261	KJ140262
KJ140263	KJ140264	KJ140265	KJ140266	KJ769147	KJ849767	KJ849780	KJ849784
KJ849785	KJ849790	KJ849796	KJ849799	KJ849803	KJ849804	KJ849807	KJ849808
KJ849811	KJ849812	KJ849814	KJ849817	KJ849818	KJ849820	KJ849821	KJ849825
KJ948656	KJ948660	KM217584	KM217662	KM218138	KP109510	KP109511	KP109512
KP109514	KP109515	KP109518	KP411822	KP411823	KP411824	KP411825	KP411827
KP411828	KP411829	KP874105	KP874107	KP874109	KP874110	KP874111	KP874113
KP874114	KP874116	KP874117	KP874118	KP874121	KP874122	KP874123	KP874127
KP874129	KP874132	KP874134	KP874136	KP874137	KP874138	KP874141	KP874142
KP874143	KP874144	KP874145	KP874147	KP874151	KP874152	KP874153	KP874155
KP874156	KP874157	KP874160	KP874162	KP874164	KP874165	KP874167	KP874168
KP874169	KP874170	KP874172	KP874173	KP874174	KP874175	KP874179	KP874180
KR914675	KR914676	KT200348	KT200349	KT200350	KT200351	KT200352	KT200353
KT200354	KT200355	KT200356	KT200357	KT200358	KT276256	KT284371	KT860442
KT860444	KT860447	KT860453	KT860454	KT860456	KT860458	KT860459	KT860464
KT860466	KT860468	KT860469	KT860471	KT860473	KT860475	KT860477	KT860480
KT860482	KT860488	KT860489	KT860490	KT860491	KT860494	KT860499	KT860500
KT860502	KT860504	KT860506	L02317	M17449	M17451	M26727	M38429
M38431	U21135	U23487	U34604	U39362	U41056	U41181	U42229
U42235	U42246	U42254	U42261	U42268	U42271	U42272	U42274
U42275	U42276	U42277	U42279	U42281	U42282	U43096	U71182
Z30601	Z30602	Z30603	Z30604	Z30605	Z30606	Z30610	Z30611
Z30612	Z30613	Z30614	Z30615	Z30616	Z30617	Z30619	Z30620
Z30622	Z30623	Z30624	Z30626	Z30627	Z30628	Z30629	Z30630
Z30631	Z30633	Z30634	Z30636	Z30638	Z30639	Z30640	Z30641
Z30642	Z30661	Z30679	Z30680	Z30681	Z30683	Z30684	Z30685
Z30686	Z30687	Z30689	Z30690	Z30691	Z30692	Z30693	Z30694
Z30695	Z30696						
