# Supplementary material for: A scale-free analysis of the HIV-1 genome demonstrates multiple conserved regions of structural and functional importance
Source: PLoS Comput Biol. 2019 Sep 23;15(9):e1007345. doi: 10.1371/journal.pcbi.1007345 (PMC6791557; doi:10.1371/journal.pcbi.1007345)
Supplement: S4 Table — (PDF) [file pcbi.1007345.s035.pdf]

AB034517	AB034521	AB034524	AB034535	AB034536	AB034540	AB034543	AB034546
AB097870	AB221126	AB286956	AB287363	AB287364	AB287367	AB287368	AB287372
AB289588	AB289590	AB428551	AB428552	AB428558	AB480694	AB480696	AB480698
AB565497	AB641836	AB731663	AB731665	AB731667	AB731669	AF000323	AF042102
AF069140	AF086817	AF133402	AF133403	AF133404	AF133405	AF146728	AF224507
AF286365	AF316095	AF316096	AF316098	AF538302	AF538303	AF538304	AF538307
AJ437490	AJ437493	AJ437496	AJ437499	AJ437502	AY006014	AY037268	AY037269
AY037270	AY037282	AY064295	AY173951	AY173952	AY173953	AY173955	AY173959
AY173960	AY180905	AY314061	AY331282	AY331290	AY331292	AY331294	AY331296
AY332237	AY352275	AY423381	AY444333	AY560107	AY560109	AY560110	AY561236
AY561237	AY561238	AY561240	AY561242	AY561244	AY586542	AY713410	AY713411
AY751406	AY751407	AY779553	AY779557	AY781126	AY781127	AY795904	AY795905
AY818644	AY835753	AY835763	AY835773	AY835774	AY835781	AY839827	AY856778
AY856779	AY856782	AY856783	AY856784	AY856787	AY856788	AY856789	AY856790
AY856791	AY856792	AY856793	AY856796	AY856801	AY856802	AY856804	AY856805
AY856806	AY856808	AY856809	AY856811	AY856813	AY856814	AY856815	AY856816
AY856817	AY856818	AY856820	AY856823	AY856824	AY856827	AY856829	AY857022
AY857127	AY857144	AY857165	D10112	DQ127534	DQ127537	DQ127548	DQ207940
DQ207942	DQ295192	DQ322223	DQ322225	DQ322227	DQ354112	DQ354116	DQ354118
DQ354119	DQ358805	DQ358808	DQ358809	DQ358810	DQ383748	DQ383749	DQ383750
DQ383751	DQ396398	DQ487188	DQ487190	DQ676875	DQ823362	DQ823364	DQ837381
DQ853436	DQ854716	DQ874474	DQ874476	DQ874477	DQ874478	DQ874480	DQ874484
DQ874486	DQ874488	DQ874490	DQ874491	DQ874493	DQ874494	DQ874507	DQ874508
DQ874509	DQ874511	DQ874514	DQ874515	DQ874518	DQ874519	DQ874520	DQ874522
DQ874523	DQ874524	DQ874526	DQ874529	DQ874532	DQ886031	DQ886032	DQ886033
DQ886034	DQ886036	DQ886037	DQ990880	EF125642	EF175212	EF178310	EF178322
EF178358	EF178427	EF363123	EF363126	EF363127	EF514697	EF514698	EF514699
EF514701	EF514702	EF514703	EF514704	EF514705	EF514706	EF514709	EF514710
EF514711	EF514712	EF637046	EF637047	EF637048	EF637049	EF637051	EF637053
EF637054	EF637056	EF637057	EF694037	EU518089	EU518091	EU518095	EU518098
EU518101	EU518103	EU518104	EU518106	EU518111	EU518112	EU518128	EU616649
EU786674	EU786675	EU786676	EU786678	EU786679	EU786680	EU839596	EU839597
EU839598	EU839600	EU839602	EU839604	EU839605	EU839607	EU839608	EU839609
FJ039758	FJ039778	FJ039779	FJ039780	FJ195086	FJ195088	FJ195089	FJ195090
FJ195091	FJ388890	FJ388891	FJ388895	FJ388904	FJ388905	FJ388910	FJ388911
FJ388912	FJ388915	FJ388916	FJ388918	FJ388919	FJ388927	FJ388930	FJ388933
FJ388934	FJ388935	FJ388939	FJ388940	FJ388941	FJ388949	FJ388955	FJ388956
FJ388957	FJ388958	FJ388964	FJ388965	FJ403482	FJ460499	FJ469682	FJ469683
FJ469684	FJ469686	FJ469687	FJ469688	FJ469689	FJ469692	FJ469693	FJ469694
FJ469696	FJ469697	FJ469698	FJ469699	FJ469700	FJ469701	FJ469702	FJ469703
FJ469706	FJ469707	FJ469709	FJ469710	FJ469711	FJ469712	FJ469713	FJ469714
FJ469715	FJ469716	FJ469717	FJ469718	FJ469719	FJ469721	FJ469722	FJ469723
FJ469726	FJ469727	FJ469729	FJ469731	FJ469734	FJ469735	FJ469737	FJ469738
FJ469739	FJ469740	FJ469741	FJ469742	FJ469743	FJ469744	FJ469745	FJ469747
FJ469748	FJ469749	FJ469750	FJ469751	FJ469752	FJ469755	FJ469756	FJ469757
FJ469758	FJ469759	FJ469760	FJ469763	FJ469764	FJ469766	FJ469767	FJ469768
FJ469769	FJ469770	FJ469771	FJ495818	FJ495941	FJ496000	FJ496078	FJ496081
FJ496145	FJ853620	GQ256641	GQ358531	GQ859356	GU177863	GU331147	GU331247
GU362881	GU362883	GU367571	GU562058	GU562155	GU730556	GU730792	GU730906
GU730907	GU730976	GU731054	HM030561	HQ215554	HQ215556	HQ846896	JF320003
JF320008	JF320013	JF320018	JF320019	JF320028	JF320036	JF320038	JF320043
JF320045	JF320048	JF320053	JF320054	JF320059	JF320097	JF320101	JF320126
JF320130	JF320145	JF320150	JF320151	JF320159	JF320160	JF320169	JF320174
JF320182	JF320183	JF320184	JF320185	JF320189	JF320191	JF320197	JF320208
JF320215	JF320226	JF320228	JF320244	JF320263	JF320277	JF320307	JF320316
JF320347	JF320349	JF320356	JF320361	JF320363	JF320381	JF320386	JF320413
JF320424	JF320427	JF320437	JF320467	JF320484	JF320526	JF320530	JF320537

JF320563	JF320564	JF320577	JF320594	JF320613	JF320615	JF320634	JF683736
JF683738	JF683741	JF683742	JF683743	JF683747	JF683749	JF683750	JF683751
JF683753	JF683754	JF683756	JF683764	JF683765	JF683769	JF683773	JF683775
JF683778	JF683781	JF683784	JF683785	JF683787	JF683788	JF683790	JF683793
JF683794	JF683796	JF683797	JF683801	JF683804	JF683805	JF683807	JF689852
JF689854	JF689856	JF689857	JF689859	JF689860	JF689862	JF689863	JF689864
JF689865	JF689866	JF689867	JF689870	JF689871	JF689872	JF689874	JF689875
JF689876	JF689877	JF689883	JF689885	JF689886	JF689889	JF689890	JF689892
JF689893	JF689895	JF689896	JF932468	JF932469	JF932470	JF932471	JF932472
JF932473	JF932474	JF932475	JF932476	JF932477	JF932478	JF932479	JF932480
JF932481	JF932482	JF932483	JF932484	JF932485	JF932486	JF932487	JF932488
JF932489	JF932490	JF932491	JF932492	JF932493	JF932494	JF932495	JF932496
JF932497	JF932498	JF932499	JF932500	JF957870	JF957872	JF957874	JF957881
JF957883	JF957885	JF957889	JF957891	JF957892	JF957928	JF957931	JF957932
JF957949	JF957982	JF957984	JF957985	JF957986	JF957991	JF957993	JF957994
JF958004	JF958007	JF958011	JF958065	JF958068	JN024100	JN024210	JN024303
JN024344	JN024363	JN024428	JN125863	JN125864	JN125865	JN125866	JN125867
JN125869	JN125870	JN125871	JN125872	JN125873	JN125875	JN125876	JN125879
JN125881	JN125882	JN125883	JN125884	JN125885	JN125886	JN125887	JN125889
JN125890	JN125891	JN125892	JN125893	JN125894	JN125895	JN125896	JN125897
JN125898	JN125899	JN125900	JN125901	JN125902	JN125906	JN125907	JN125908
JN125909	JN125910	JN125911	JN125912	JN125913	JN125914	JN125915	JN125916
JN125917	JN125918	JN125919	JN125920	JN125922	JN125923	JN125924	JN125926
JN125927	JN125928	JN125930	JN125931	JN125932	JN125933	JN125934	JN125935
JN125936	JN125937	JN125938	JN125939	JN125940	JN125941	JN125942	JN125943
JN125944	JN125945	JN125947	JN125948	JN125949	JN125951	JN125952	JN125953
JN125956	JN125958	JN125959	JN125960	JN125961	JN125962	JN125963	JN125964
JN125965	JN125967	JN125968	JN125969	JN125970	JN125971	JN125972	JN125973
JN125974	JN125976	JN125977	JN125978	JN125979	JN125980	JN125981	JN125982
JN125984	JN125985	JN125986	JN125987	JN125988	JN125989	JN125990	JN125991
JN125992	JN125993	JN125994	JN125996	JN125997	JN125998	JN125999	JN126000
JN126001	JN126002	JN126003	JN126005	JN126006	JN126008	JN126010	JN126011
JN126012	JN126013	JN126014	JN126015	JN126017	JN126019	JN126020	JN126022
JN223243	JN235958	JN235965	JN248321	JN248329	JN248333	JN248335	JN248337
JN248343	JN248344	JN248346	JN248347	JN248348	JN248353	JN248354	JN251901
JN397362	JN397364	JN397365	JN400469	JN599165	JN687665	JN687675	JN687677
JN687678	JN687679	JN687680	JN687690	JN687691	JN687749	JN692432	JN692433
JN692435	JN692439	JN692440	JN692443	JN692444	JN692445	JN692447	JN692450
JN692451	JN692452	JN692453	JN692454	JN692455	JN692457	JN692459	JN692460
JN692461	JN692462	JN692463	JN692465	JN692467	JN692468	JN692470	JN692471
JN692473	JN692474	JN692475	JN692479	JN692480	JN860769	JN944897	JN944905
JN944907	JN944909	JN944911	JN944917	JN944928	JN944930	JN944936	JN944938
JQ066995	JQ067045	JQ248203	JQ248313	JQ269041	JQ269060	JQ269106	JQ269150
JQ269176	JQ269187	JQ269218	JQ316126	JQ316127	JQ316128	JQ316129	JQ316130
JQ316131	JQ316132	JQ316133	JQ316134	JQ316135	JQ327728	JQ327767	JQ327782
JQ341411	JQ403019	JQ403020	JQ403021	JQ403022	JQ403023	JQ403024	JQ403025
JQ403026	JQ403029	JQ403031	JQ403035	JQ403037	JQ403042	JQ403043	JQ403044
JQ403045	JQ403046	JQ403047	JQ403048	JQ403056	JQ403058	JQ403059	JQ403060
JQ403061	JQ403062	JQ403063	JQ403064	JQ403065	JQ403066	JQ403067	JQ403068
JQ403069	JQ403070	JQ403071	JQ403072	JQ403074	JQ403075	JQ403077	JQ403078
JQ403079	JQ403080	JQ403081	JQ403082	JQ403083	JQ403084	JQ403085	JQ403086
JQ403087	JQ403088	JQ403089	JQ403091	JQ403092	JQ403093	JQ403094	JQ403095
JQ403096	JQ403097	JQ403098	JQ403100	JQ403102	JQ403103	JQ403104	JQ403105
JQ403106	JQ403107	JQ416158	JQ429433	JX140652	JX140654	JX140656	JX140657
JX140659	JX446800	JX446807	JX447156	JX447790	JX448096	JX500707	JX500708
JX500709	JX503075	JX863919	JX863921	JX960597	JX960598	JX960599	JX972342
JX974238	KC312386	KC312435	KC312510	KC312583	KC473824	KC473825	KC473826

KC473827	KC473828	KC473829	KC473830	KC473831	KC473832	KC473833	KC473834
KC473835	KC473841	KC473842	KC473846	KC596066	KC596067	KC596069	KC797171
KC797225	KC899011	KC935957	KC935958	KC935959	KF384798	KF384799	KF384800
KF384801	KF384802	KF384803	KF384804	KF384805	KF384806	KF384807	KF384808
KF384810	KF384811	KF384812	KF384813	KF384814	KF526141	KF526228	KF526265
KF526312	KF526323	KF561441	KF561442	KF716494	KF716495	KF716496	KF716497
KF716498	KF990605	KF990608	KJ019215	KJ140249	KJ140250	KJ140251	KJ140255
KJ140256	KJ140261	KJ140262	KJ140263	KJ140264	KJ140265	KJ140266	KJ769147
KJ849767	KJ849780	KJ849784	KJ849785	KJ849788	KJ849790	KJ849796	KJ849799
KJ849801	KJ849803	KJ849804	KJ849805	KJ849807	KJ849808	KJ849811	KJ849812
KJ849814	KJ849815	KJ849817	KJ849818	KJ849820	KJ849821	KJ849825	KJ948657
KM217584	KM217662	KM217939	KM218138	KM218168	KM502968	KP109511	KP109512
KP109514	KP109515	KP109518	KP411822	KP411823	KP411824	KP411825	KP411827
KP411828	KR914678	KT200348	KT200349	KT200350	KT200351	KT200352	KT200353
KT200354	KT200355	KT200356	KT200357	KT200358	KT276256	KT276262	KT276263
KT276266	KT276267	KT276268	KT284371	L02317	M17450	M17451	M38431
U23487	U34604	U39362	U41705	U41707	U41710	U41723	U43096
U71182	U73371	U73373	Z68542	Z68543	Z68552	Z68554	Z68555
Z68556	Z68558	Z68559	Z68562	Z68566	Z68567	Z68571	Z68573
Z68575	Z68579	Z68584					
