# Supplementary material for: A scale-free analysis of the HIV-1 genome demonstrates multiple conserved regions of structural and functional importance
Source: PLoS Comput Biol. 2019 Sep 23;15(9):e1007345. doi: 10.1371/journal.pcbi.1007345 (PMC6791557; doi:10.1371/journal.pcbi.1007345)
Supplement: S5 Table — (PDF) [file pcbi.1007345.s036.pdf]

AB078005	AB221126	AB287363	AB287367	AB287368	AB287372	AB289588	AB289590
AB428551	AB428553	AB428556	AB480692	AB480694	AB480696	AF004394	AF042101
AF042102	AF042103	AF042104	AF042105	AF049495	AF069140	AF146728	AF224507
AF286365	AF491739	AF538302	AF538304	AF538305	AF538306	AF538307	AY037268
AY037269	AY037270	AY037282	AY173951	AY173952	AY173953	AY173954	AY173955
AY173959	AY173960	AY180905	AY247251	AY308760	AY314044	AY331282	AY331284
AY331285	AY331287	AY331289	AY331291	AY331292	AY331294	AY331296	AY352275
AY423381	AY560107	AY560108	AY560110	AY561236	AY561237	AY561238	AY586542
AY586543	AY608577	AY624304	AY682547	AY713410	AY751407	AY779550	AY779557
AY781126	AY781127	AY795905	AY818644	AY835749	AY835753	AY835758	AY835761
AY835763	AY835768	AY835773	AY835774	AY857022	AY857144	D10112	DQ007902
DQ127534	DQ127537	DQ207942	DQ295195	DQ295196	DQ322227	DQ354118	DQ354119
DQ358805	DQ358808	DQ358809	DQ383746	DQ383748	DQ383750	DQ383751	DQ396398
DQ672623	DQ676875	DQ823362	DQ823363	DQ823364	DQ853463	DQ886031	DQ886032
DQ886033	DQ886036	DQ886037	DQ990880	EF175212	EF178314	EF178420	EF178427
EF363123	EF363126	EF363127	EF514697	EF514698	EF514699	EF514700	EF514701
EF514703	EF514705	EF514706	EF514708	EF514711	EF637046	EF637047	EF637048
EF637049	EF637050	EF637051	EF637053	EF637054	EF637056	EF637057	EF694037
EU616649	EU786672	EU786676	EU786677	EU786679	EU786680	EU839603	EU839605
EU839607	FJ195086	FJ195088	FJ195089	FJ195090	FJ388890	FJ388891	FJ388899
FJ388904	FJ388905	FJ388910	FJ388912	FJ388915	FJ388919	FJ388927	FJ388930
FJ388933	FJ388934	FJ388935	FJ388940	FJ388949	FJ388955	FJ388956	FJ388959
FJ388960	FJ388962	FJ388963	FJ388964	FJ388965	FJ403482	FJ460499	FJ469682
FJ469683	FJ469684	FJ469685	FJ469686	FJ469687	FJ469689	FJ469690	FJ469691
FJ469692	FJ469693	FJ469695	FJ469696	FJ469697	FJ469698	FJ469699	FJ469700
FJ469701	FJ469702	FJ469703	FJ469704	FJ469705	FJ469707	FJ469708	FJ469709
FJ469710	FJ469711	FJ469712	FJ469713	FJ469714	FJ469715	FJ469716	FJ469717
FJ469718	FJ469719	FJ469721	FJ469722	FJ469723	FJ469725	FJ469726	FJ469727
FJ469728	FJ469729	FJ469730	FJ469731	FJ469734	FJ469735	FJ469737	FJ469738
FJ469739	FJ469740	FJ469741	FJ469742	FJ469743	FJ469744	FJ469747	FJ469748
FJ469750	FJ469751	FJ469752	FJ469753	FJ469756	FJ469757	FJ469758	FJ469759
FJ469760	FJ469761	FJ469763	FJ469764	FJ469766	FJ469767	FJ469768	FJ469769
FJ469770	FJ469771	FJ495818	FJ495941	FJ496000	FJ496078	FJ496145	FJ496151
FJ496169	FJ647145	K02007	KJ140249	KJ140250	KJ140251	KJ140255	KJ140256
KJ140261	KJ140262	KJ140263	KJ140264	KJ140265	KJ140266	KJ849767	KJ849780
KJ849784	KJ849785	KJ849788	KJ849790	KJ849796	KJ849799	KJ849801	KJ849803
KJ849807	KJ849808	KJ849812	KJ849814	KJ849817	KJ849820	KJ849821	KJ849825
KJ948656	KJ948660	M17449	M17450	M26727	M37573	M93258	U04908
U21135	U23487	U39362	U43141	U71182			
