# Supplementary material for: A scale-free analysis of the HIV-1 genome demonstrates multiple conserved regions of structural and functional importance
Source: PLoS Comput Biol. 2019 Sep 23;15(9):e1007345. doi: 10.1371/journal.pcbi.1007345 (PMC6791557; doi:10.1371/journal.pcbi.1007345)
Supplement: S6 Table — (PDF) [file pcbi.1007345.s037.pdf]

AB078005	AB221005	AB221126	AB287363	AB287364	AB287367	AB287368	AB287372
AB289588	AB289590	AB428551	AB428553	AB428558	AB480692	AB480694	AB480696
AB480698	AB564745	AB564746	AB565478	AB565496	AB565497	AB565502	AB604946
AB604948	AB641836	AB731663	AB731665	AB731669	AF003887	AF004394	AF042100
AF042101	AF042102	AF042103	AF042104	AF042105	AF049495	AF069140	AF086817
AF146728	AF224507	AF286365	AF490512	AF491737	AF538302	AF538304	AF538305
AF538306	AF538307	AJ271445	AY037268	AY037269	AY037270	AY037282	AY173951
AY173952	AY173953	AY173955	AY173959	AY173960	AY308760	AY314061	AY331282
AY331284	AY331287	AY331289	AY331292	AY331294	AY331296	AY332237	AY352275
AY423381	AY560107	AY560108	AY560109	AY560110	AY561236	AY561237	AY561238
AY561240	AY586542	AY586543	AY608576	AY624304	AY713410	AY751407	AY779558
AY781126	AY781127	AY795904	AY795905	AY818644	AY835749	AY835753	AY835758
AY835761	AY835768	AY835769	AY835773	AY835779	AY839827	AY857022	AY857144
AY945710	AY945711	D10112	DQ127537	DQ127548	DQ207940	DQ207942	DQ295193
DQ295195	DQ295196	DQ322223	DQ322225	DQ322227	DQ354112	DQ354118	DQ354119
DQ358805	DQ358808	DQ358809	DQ358810	DQ383746	DQ383748	DQ383749	DQ383751
DQ396398	DQ487188	DQ672623	DQ676881	DQ823364	DQ837381	DQ853463	DQ886031
DQ886032	DQ886033	DQ886034	DQ886036	DQ886037	DQ990880	EF175212	EF178314
EF178420	EF363123	EF363126	EF363127	EF514697	EF514698	EF514699	EF514700
EF514701	EF514702	EF514705	EF514706	EF514707	EF514708	EF514711	EF593269
EF593271	EF593272	EF593273	EF593274	EF593275	EF593276	EF637046	EF637047
EF637048	EF637049	EF637050	EF637051	EF637053	EF637054	EF637056	EF637057
EF694037	EU289184	EU289185	EU289186	EU289188	EU289189	EU289190	EU289191
EU289192	EU289195	EU289196	EU289197	EU289198	EU289199	EU289200	EU289201
EU363827	EU363828	EU363829	EU363830	EU547186	EU616649	EU786672	EU786676
EU786677	EU786678	EU786680	EU839601	EU839603	EU839605	EU839606	EU839607
EU839608	FJ195086	FJ195088	FJ195089	FJ195090	FJ195091	FJ388890	FJ388891
FJ388895	FJ388898	FJ388899	FJ388904	FJ388905	FJ388910	FJ388911	FJ388912
FJ388915	FJ388919	FJ388927	FJ388930	FJ388931	FJ388933	FJ388934	FJ388935
FJ388937	FJ388941	FJ388955	FJ388957	FJ388958	FJ388959	FJ388960	FJ388964
FJ388965	FJ469682	FJ469683	FJ469684	FJ469685	FJ469686	FJ469687	FJ469688
FJ469689	FJ469690	FJ469691	FJ469692	FJ469693	FJ469694	FJ469695	FJ469696
FJ469697	FJ469698	FJ469699	FJ469700	FJ469701	FJ469702	FJ469704	FJ469705
FJ469706	FJ469707	FJ469709	FJ469710	FJ469711	FJ469712	FJ469713	FJ469714
FJ469715	FJ469716	FJ469718	FJ469721	FJ469722	FJ469723	FJ469725	FJ469726
FJ469727	FJ469728	FJ469729	FJ469730	FJ469731	FJ469732	FJ469734	FJ469737
FJ469738	FJ469739	FJ469740	FJ469741	FJ469742	FJ469743	FJ469744	FJ469745
FJ469747	FJ469748	FJ469750	FJ469751	FJ469752	FJ469753	FJ469756	FJ469757
FJ469758	FJ469761	FJ469763	FJ469766	FJ469767	FJ469768	FJ469769	FJ469770
FJ469771	FJ495941	FJ496000	FJ496078	FJ496081	FJ496145	FJ496151	FJ496169
FJ853620	FJ853622	GQ372988	GQ372990	GU331147	GU331247	GU362881	GU362883
GU362885	GU362886	GU562001	GU562033	GU562058	GU562080	GU562135	GU562155
GU562236	GU562266	GU647196	GU733713	HM030559	HM030560	HM030561	HM030562
HM030564	HM030565	HM586187	HM586198	HM586209	HQ846911	JF320003	JF320008
JF320011	JF320013	JF320018	JF320019	JF320028	JF320036	JF320038	JF320043
JF320045	JF320048	JF320053	JF320054	JF320097	JF320117	JF320126	JF320131
JF320145	JF320151	JF320160	JF320169	JF320173	JF320182	JF320183	JF320184
JF320185	JF320189	JF320191	JF320197	JF320208	JF320215	JF320226	JF320230
JF320244	JF320263	JF320279	JF320315	JF320329	JF320356	JF320361	JF320363
JF320375	JF320385	JF320387	JF320394	JF320413	JF320424	JF320484	JF320502
JF320526	JF320530	JF320539	JF320563	JF320564	JF320592	JF320613	JF320615
JF320631	JF683736	JF683738	JF683741	JF683742	JF683743	JF683747	JF683749
JF683750	JF683751	JF683753	JF683754	JF683756	JF683764	JF683765	JF683769
JF683773	JF683775	JF683778	JF683781	JF683785	JF683787	JF683790	JF683793
JF683794	JF683796	JF683797	JF683801	JF683804	JF683805	JF683807	JF689852
JF689856	JF689857	JF689859	JF689860	JF689862	JF689863	JF689865	JF689866
JF689867	JF689870	JF689871	JF689872	JF689873	JF689874	JF689875	JF689876

JF689877	JF689886	JF689889	JF689890	JF689893	JF689895	JF689896	JF932468
JF932469	JF932470	JF932471	JF932472	JF932473	JF932474	JF932475	JF932476
JF932477	JF932478	JF932479	JF932480	JF932481	JF932483	JF932484	JF932485
JF932486	JF932487	JF932488	JF932489	JF932490	JF932491	JF932492	JF932493
JF932494	JF932495	JF932496	JF932497	JF932498	JF932499	JF932500	JN024100
JN024210	JN024303	JN024344	JN024428	JN235958	JN235965	JN248329	JN248333
JN248335	JN248337	JN248343	JN248346	JN248347	JN248353	JN248354	JN251896
JN251901	JN397362	JN397364	JN397365	JN400469	JN599165	JN687739	JN687749
JN687750	JN687759	JN687760	JN687761	JN687762	JN687763	JN687773	JN692432
JN692433	JN692435	JN692439	JN692440	JN692443	JN692444	JN692445	JN692447
JN692450	JN692451	JN692453	JN692454	JN692455	JN692457	JN692459	JN692460
JN692461	JN692462	JN692463	JN692465	JN692468	JN692470	JN692471	JN692473
JN692475	JN692479	JN860769	JN944897	JN944905	JN944907	JN944909	JN944911
JN944928	JN944930	JN944936	JN944938	JQ316131	JQ316134	JQ403019	JQ403021
JQ403022	JQ403023	JQ403024	JQ403026	JQ403029	JQ403031	JQ403035	JQ403037
JQ403042	JQ403043	JQ403044	JQ403045	JQ403046	JQ403047	JQ403048	JQ403058
JQ403059	JQ403060	JQ403061	JQ403062	JQ403063	JQ403064	JQ403066	JQ403067
JQ403068	JQ403070	JQ403074	JQ403075	JQ403077	JQ403078	JQ403079	JQ403080
JQ403082	JQ403083	JQ403084	JQ403085	JQ403086	JQ403087	JQ403088	JQ403089
JQ403091	JQ403092	JQ403093	JQ403095	JQ403096	JQ403097	JQ403100	JQ403102
JQ403103	JQ403104	JQ403105	JQ403106	JQ403107	JQ416158	JX140653	JX140654
JX140656	JX140657	JX140659	JX446800	JX446818	JX447156	JX447795	JX500707
JX500708	JX500709	JX863921	JX960597	JX960598	JX960599	JX972342	JX974238
K02007	K03455	KC312386	KC312435	KC312470	KC312583	KC473825	KC473826
KC473827	KC473828	KC473829	KC473830	KC473831	KC473832	KC473833	KC473834
KC473835	KC473841	KC473842	KC473846	KC596067	KC797171	KC797225	KC899011
KC935957	KC935958	KC935959	KF384798	KF384799	KF384800	KF384802	KF384803
KF384804	KF384805	KF384806	KF384807	KF384808	KF384810	KF384811	KF384812
KF384813	KF384814	KF526141	KF526228	KF526312	KF526323	KF716494	KF716495
KF716496	KF716497	KF990605	KF990608	KJ019215	KJ769147	KJ849767	KJ849780
KJ849784	KJ849785	KJ849786	KJ849788	KJ849790	KJ849796	KJ849801	KJ849803
KJ849807	KJ849808	KJ849811	KJ849817	KJ849820	KJ849821	KJ849825	KM217802
KP109514	KP109515	KP109518	KP411824	KP411828	KP411829	KR914675	KR914676
KR914678	KT200348	KT200350	KT200351	KT200352	KT200353	KT200354	KT200355
KT200357	KT200358	KT276255	KT276256	KT276262	KT276263	KT276266	KT276268
M17449	M17450	M17451	M38431	M93258	U04908	U21135	U23487
U39362	U43096	U43141	U71182				
