# Supplementary material for: A scale-free analysis of the HIV-1 genome demonstrates multiple conserved regions of structural and functional importance
Source: PLoS Comput Biol. 2019 Sep 23;15(9):e1007345. doi: 10.1371/journal.pcbi.1007345 (PMC6791557; doi:10.1371/journal.pcbi.1007345)
Supplement: S7 Table — (PDF) [file pcbi.1007345.s038.pdf]

AB034561	AB034563	AB034564	AB034565	AB034567	AB034568	AB034572	AB034576
AB034578	AB034579	AB078005	AB089254	AB089255	AB097870	AB126846	AB126849
AB221005	AB221126	AB286956	AB287363	AB287364	AB287368	AB287372	AB289588
AB289590	AB428551	AB428558	AB480692	AB480694	AB480696	AB480698	AB485638
AB564744	AB564746	AB565495	AB565497	AB565501	AB604946	AB604948	AB604950
AB731663	AB731665	AB731667	AB731669	AF003887	AF042102	AF049495	AF069140
AF086817	AF146728	AF220462	AF220463	AF220464	AF220465	AF220467	AF220468
AF220470	AF220471	AF224507	AF454471	AF454479	AF454488	AF454489	AF491740
AF538302	AF538304	AF538305	AF538306	AJ437514	AJ437519	AJ437522	AJ437523
AJ437526	AJ437529	AY006056	AY006086	AY037268	AY037269	AY037270	AY037282
AY137614	AY137615	AY137616	AY137618	AY137619	AY137621	AY137626	AY137627
AY137628	AY137629	AY137632	AY137633	AY137634	AY137636	AY137637	AY137638
AY137639	AY137641	AY137642	AY137646	AY173952	AY173954	AY173955	AY173956
AY173959	AY173960	AY180905	AY308760	AY331282	AY331284	AY331285	AY331287
AY331289	AY331294	AY331296	AY332237	AY423381	AY560107	AY560108	AY560109
AY560110	AY561236	AY561237	AY561238	AY561240	AY561242	AY561244	AY586542
AY586543	AY608577	AY624305	AY713410	AY713411	AY751406	AY751407	AY779555
AY779559	AY781126	AY781127	AY795904	AY795905	AY835434	AY835435	AY835436
AY835437	AY835438	AY835439	AY835440	AY835441	AY835443	AY835444	AY835445
AY835446	AY835447	AY835448	AY835449	AY835452	AY835749	AY835768	AY835774
AY839827	AY856961	AY857022	DQ127534	DQ127542	DQ207940	DQ207942	DQ295192
DQ295193	DQ295195	DQ295196	DQ322223	DQ322225	DQ322227	DQ339425	DQ339432
DQ339441	DQ339443	DQ339444	DQ339457	DQ354112	DQ354114	DQ354119	DQ358805
DQ358808	DQ358809	DQ383748	DQ383749	DQ383750	DQ383752	DQ396398	DQ487188
DQ487190	DQ672623	DQ676875	DQ676881	DQ676886	DQ823362	DQ823364	DQ837381
DQ853463	DQ854716	DQ874534	DQ874536	DQ874540	DQ874544	DQ874546	DQ874547
DQ874557	DQ874559	DQ874564	DQ874566	DQ874569	DQ874570	DQ874572	DQ874575
DQ874578	DQ874579	DQ874581	DQ874582	DQ874584	DQ874585	DQ874586	DQ874587
DQ874588	DQ874589	DQ874590	DQ874591	DQ874592	DQ874593	DQ874594	DQ874595
DQ874596	DQ874597	DQ874598	DQ874602	DQ886031	DQ886032	DQ886033	DQ886034
DQ886035	DQ886036	DQ886037	DQ987950	DQ988005	DQ988017	DQ988025	EF015093
EF015107	EF015139	EF119850	EF119869	EF119871	EF119872	EF119877	EF119884
EF119893	EF119898	EF119899	EF119900	EF119915	EF119916	EF119919	EF119922
EF119926	EF119929	EF119933	EF119945	EF119947	EF119949	EF119959	EF119962
EF119969	EF119973	EF119976	EF119989	EF119990	EF120023	EF120026	EF120028
EF120032	EF120033	EF120036	EF120037	EF120039	EF120042	EF120046	EF120049
EF120054	EF120055	EF120061	EF120062	EF120064	EF120072	EF120073	EF175212
EF178374	EF363126	EF363127	EF514697	EF514698	EF514699	EF514700	EF514701
EF514702	EF514704	EF514705	EF514706	EF514708	EF514709	EF514710	EF514711
EF514712	EF593269	EF593271	EF593273	EF593274	EF593275	EF593300	EF593304
EF593312	EF593314	EF593315	EF593316	EF593318	EF637047	EF637048	EF637049
EF637050	EF637051	EF637053	EF637054	EF637057	EF694037	EU023916	EU023919
EU023921	EU023922	EU023923	EU023927	EU023928	EU023929	EU023932	EU023933
EU289185	EU289188	EU289190	EU289192	EU289195	EU289196	EU289197	EU289199
EU289201	EU312189	EU363825	EU363827	EU363829	EU363830	EU517721	EU517723
EU517725	EU517731	EU517732	EU517734	EU517736	EU517737	EU517739	EU517743
EU517746	EU517747	EU517748	EU517749	EU517750	EU517751	EU517752	EU517754
EU517759	EU517760	EU517761	EU547186	EU575474	EU575593	EU575697	EU575850
EU575927	EU576016	EU577811	EU577862	EU578032	EU616649	EU786672	EU786677
EU786678	EU786679	EU786680	EU807824	EU839596	EU839604	EU839605	FJ195086
FJ195088	FJ195089	FJ195090	FJ195091	FJ388890	FJ388898	FJ388899	FJ388905
FJ388910	FJ388911	FJ388912	FJ388915	FJ388918	FJ388919	FJ388920	FJ388927
FJ388931	FJ388933	FJ388934	FJ388935	FJ388936	FJ388940	FJ388941	FJ388947
FJ388949	FJ388955	FJ388956	FJ388957	FJ388958	FJ388963	FJ388964	FJ388965
FJ403482	FJ460501	FJ469682	FJ469683	FJ469684	FJ469685	FJ469686	FJ469687
FJ469688	FJ469689	FJ469690	FJ469691	FJ469692	FJ469693	FJ469694	FJ469695
FJ469696	FJ469698	FJ469699	FJ469700	FJ469701	FJ469702	FJ469703	FJ469706

FJ469707	FJ469708	FJ469709	FJ469710	FJ469711	FJ469712	FJ469713	FJ469714
FJ469715	FJ469717	FJ469718	FJ469719	FJ469721	FJ469723	FJ469725	FJ469726
FJ469727	FJ469728	FJ469729	FJ469730	FJ469731	FJ469732	FJ469735	FJ469736
FJ469737	FJ469738	FJ469739	FJ469740	FJ469741	FJ469742	FJ469743	FJ469744
FJ469745	FJ469747	FJ469748	FJ469749	FJ469750	FJ469751	FJ469752	FJ469753
FJ469755	FJ469756	FJ469757	FJ469758	FJ469759	FJ469760	FJ469761	FJ469763
FJ469764	FJ469766	FJ469767	FJ469768	FJ469769	FJ469770	FJ469771	FJ469772
FJ495818	FJ495941	FJ496000	FJ496078	FJ496081	FJ496145	FJ496151	FJ496169
FJ515874	FJ670524	FJ670525	FJ670531	FJ694790	FJ853620	FJ853622	FJ861953
FW371650	GQ256643	GQ358530	GQ372988	GQ372990	GQ386774	GQ925947	GU362881
GU362883	GU362885	GU362886	GU367572	GU367575	GU367576	GU367581	GU367582
GU367583	GU367584	GU367585	GU367586	GU367588	GU367589	GU367592	GU453424
GU562001	GU562028	GU562058	GU562080	GU562105	GU562134	GU562153	GU562218
GU562249	GU562271	GU647196	GU730035	GU730037	GU730038	GU730040	GU730045
GU730051	GU730057	GU730060	GU730072	GU730076	GU730079	GU730080	GU730092
GU730100	GU730102	GU730105	GU730108	GU730117	GU730118	GU730127	GU730129
GU730131	GU730140	GU730145	GU730164	GU730165	GU730167	GU730189	GU730196
GU730198	GU730207	GU730210	GU730211	GU730215	GU730249	GU730251	GU730252
GU730253	GU730255	GU730256	GU730261	GU730272	GU730287	GU730289	GU730298
GU730303	GU730309	GU730318	GU730325	GU730327	GU730329	GU730333	GU730334
GU730337	GU730338	GU730340	GU730348	GU730349	GU730351	GU730354	GU730360
GU730362	GU730363	GU730366	GU730369	GU730371	GU730373	GU730375	GU730377
GU730380	GU730381	GU730382	GU730390	GU730393	GU730397	GU730403	GU730404
GU730412	GU730424	GU730425	GU730428	GU730431	GU730433	GU730435	GU730439
GU730448	GU730449	GU730453	GU730456	GU730460	GU730463	GU730469	GU730471
GU730472	GU730473	GU730480	GU730484	GU730485	GU730488	GU730492	GU730495
GU730500	GU730504	GU730509	GU730510	GU730512	GU730515	GU730522	GU730526
GU730529	GU730530	GU730533	GU730535	GU730545	GU730548	GU730550	GU730551
GU733713	HE583237	HE583239	HE583243	HE583255	HI964165	HM030560	HM030561
HM030562	HM030565	HM586187	HM586210	HQ239051	HQ258926	HQ258927	JF320014
JF320015	JF320017	JF320023	JF320024	JF320028	JF320029	JF320032	JF320034
JF320036	JF320039	JF320041	JF320045	JF320050	JF320051	JF320055	JF320056
JF320057	JF320059	JF320069	JF320081	JF320082	JF320096	JF320111	JF320117
JF320122	JF320123	JF320128	JF320134	JF320142	JF320147	JF320151	JF320158
JF320162	JF320169	JF320175	JF320179	JF320183	JF320184	JF320185	JF320187
JF320188	JF320189	JF320194	JF320196	JF320197	JF320203	JF320204	JF320205
JF320206	JF320207	JF320209	JF320211	JF320214	JF320215	JF320216	JF320217
JF320221	JF320222	JF320223	JF320224	JF320226	JF320227	JF320233	JF320238
JF320241	JF320243	JF320253	JF320259	JF320261	JF320265	JF320272	JF320273
JF320285	JF320288	JF320289	JF320291	JF320294	JF320310	JF320313	JF320317
JF320331	JF320336	JF320356	JF320361	JF320365	JF320374	JF320375	JF320380
JF320382	JF320385	JF320387	JF320398	JF320399	JF320403	JF320406	JF320422
JF320424	JF320429	JF320435	JF320440	JF320443	JF320447	JF320451	JF320452
JF320460	JF320462	JF320463	JF320464	JF320466	JF320467	JF320468	JF320470
JF320483	JF320485	JF320486	JF320487	JF320488	JF320489	JF320490	JF320491
JF320492	JF320512	JF320516	JF320529	JF320533	JF320541	JF320546	JF320547
JF320548	JF320554	JF320556	JF320562	JF320566	JF320577	JF320580	JF320583
JF320585	JF320592	JF320607	JF320614	JF320617	JF320629	JF320631	JF320632
JF320637	JF320642	JF689852	JF689854	JF689856	JF689857	JF689859	JF689860
JF689861	JF689862	JF689863	JF689865	JF689866	JF689867	JF689870	JF689871
JF689873	JF689874	JF689875	JF689876	JF689877	JF689878	JF689879	JF689880
JF689883	JF689884	JF689885	JF689886	JF689889	JF689890	JF689892	JF689893
JF689895	JF689896	JF932468	JF932469	JF932470	JF932471	JF932472	JF932473
JF932474	JF932475	JF932476	JF932477	JF932478	JF932479	JF932480	JF932481
JF932482	JF932483	JF932484	JF932485	JF932486	JF932487	JF932488	JF932489
JF932490	JF932491	JF932492	JF932493	JF932494	JF932495	JF932496	JF932497
JF932498	JF932499	JF932500	JN024100	JN024210	JN024303	JN024344	JN024428

JN024463	JN223241	JN248321	JN248329	JN248333	JN248335	JN248337	JN248344
JN248345	JN248346	JN248347	JN248353	JN248354	JN599165	JN860769	JN944897
JN944905	JN944907	JN944909	JN944911	JN944917	JN944928	JN944930	JN944936
JN944938	JQ269089	JQ316126	JQ316127	JQ316128	JQ316130	JQ316131	JQ316132
JQ316133	JQ316134	JQ316135	JQ341411	JQ403019	JQ403020	JQ403021	JQ403022
JQ403023	JQ403024	JQ403025	JQ403026	JQ403029	JQ403031	JQ403035	JQ403037
JQ403042	JQ403044	JQ403045	JQ403046	JQ403047	JQ403048	JQ403056	JQ403058
JQ403059	JQ403060	JQ403061	JQ403062	JQ403063	JQ403064	JQ403065	JQ403066
JQ403067	JQ403068	JQ403069	JQ403070	JQ403071	JQ403073	JQ403074	JQ403075
JQ403077	JQ403078	JQ403079	JQ403080	JQ403081	JQ403082	JQ403083	JQ403084
JQ403085	JQ403086	JQ403087	JQ403088	JQ403089	JQ403091	JQ403092	JQ403093
JQ403094	JQ403095	JQ403096	JQ403097	JQ403098	JQ403100	JQ403102	JQ403103
JQ403104	JQ403105	JQ403106	JQ403107	JQ416158	JQ429433	JX140652	JX140654
JX140656	JX140657	JX140658	JX140659	JX446800	JX447156	JX447795	JX448103
JX500708	JX500709	JX503071	JX503075	JX863921	JX863965	JX863966	JX863968
JX863969	JX863970	JX863971	JX863972	JX863983	JX863984	JX863985	JX863986
JX863989	JX863991	JX863992	JX863993	JX863994	JX863995	JX864006	JX864008
JX864019	JX864020	JX864021	JX864022	JX864023	JX864024	JX864025	JX864026
JX864027	JX960597	JX960598	JX960599	JX972342	JX974238	KC189066	KC189087
KC189091	KC312386	KC312435	KC312470	KC312583	KC473824	KC473825	KC473826
KC473827	KC473828	KC473829	KC473830	KC473831	KC473832	KC473833	KC473834
KC473835	KC473842	KC473846	KC596066	KC596067	KC797171	KC797225	KC899011
KC913677	KC935957	KC935958	KC935959	KF384798	KF384799	KF384800	KF384801
KF384802	KF384803	KF384804	KF384805	KF384806	KF384807	KF384808	KF384810
KF384811	KF384812	KF384813	KF384814	KF526141	KF526228	KF526265	KF526312
KF526323	KF561441	KF561442	KF716494	KF716495	KF716496	KF716497	KF716498
KF990605	KF990608	KJ019215	KJ140247	KJ140250	KJ140251	KJ140255	KJ140257
KJ140261	KJ140262	KJ140263	KJ140264	KJ140265	KJ140266	KJ849767	KJ849784
KJ849785	KJ849788	KJ849790	KJ849796	KJ849803	KJ849804	KJ849807	KJ849808
KJ849811	KJ849812	KJ849814	KJ849815	KJ849817	KJ849818	KJ849819	KJ849820
KJ849821	KJ849825	KJ948656	KJ948660	KM081852	KM081861	KM081893	KM081913
KM081963	KM082006	KM082074	KM082106	KM082128	KM082156	KM217584	KM217662
KM217802	KM217939	KM217995	KM218228	KM359883	KM359894	KM359941	KM359942
KM406317	KM656059	KM656061	KM656063	KM656064	KM656065	KM656073	KP109511
KP109512	KP109514	KP109515	KP109518	KP411822	KP411823	KP411824	KP411825
KT200348	KT200349	KT200350	KT200351	KT200352	KT200353	KT200354	KT200355
KT200356	KT200357	KT200358	KT276256	KT276262	KT276263	KT276266	KT276267
KT276268	KT284371	KT698143	KT698144	KT698145	KT698146	KT698148	KT698149
KT698150	KT698153	KT698154	KT698156	KT698158	KT698159	M21098	M38429
U24452	U39362	U43096	U71182	U84819	Z68511	Z68521	Z68522
Z68526	Z68589	Z68593	Z68604				
