# Supplementary material for: A scale-free analysis of the HIV-1 genome demonstrates multiple conserved regions of structural and functional importance
Source: PLoS Comput Biol. 2019 Sep 23;15(9):e1007345. doi: 10.1371/journal.pcbi.1007345 (PMC6791557; doi:10.1371/journal.pcbi.1007345)
Supplement: S8 Table — (PDF) [file pcbi.1007345.s039.pdf]

AB078005	AB097870	AB221126	AB262961	AB287363	AB287364	AB287367	AB287368
AB287372	AB289588	AB289590	AB480693	AB480695	AB480697	AB480698	AF025749
AF025750	AF025751	AF025752	AF025754	AF025755	AF025756	AF025759	AF025761
AF025762	AF025763	AF041125	AF041127	AF041130	AF041131	AF041133	AF041134
AF042100	AF042101	AF042102	AF042105	AF049495	AF069140	AF128126	AF146728
AF217150	AF219627	AF277055	AF277059	AF277065	AF277068	AF277070	AF277071
AF277072	AF277073	AF286365	AF321080	AF490512	AF538302	AF538304	AF538306
AJ286330	AJ286332	AJ286334	AJ286336	AJ286337	AJ286339	AJ286342	AJ417410
AJ417413	AJ417415	AJ417425	AJ417428	AJ417431	AJ418494	AJ418495	AJ418519
AJ418521	AJ418531	AJ535607	AJ535611	AJ535612	AJ535618	AY037268	AY037269
AY037270	AY037282	AY173952	AY173953	AY173956	AY173960	AY189526	AY247224
AY314061	AY331284	AY331289	AY331294	AY331296	AY332237	AY352275	AY423386
AY426119	AY426125	AY505010	AY535455	AY535471	AY560107	AY560108	AY561236
AY561237	AY561239	AY608577	AY612855	AY624305	AY713410	AY779552	AY781127
AY795904	AY795905	AY835435	AY835436	AY835437	AY835438	AY835442	AY835443
AY835444	AY835445	AY835446	AY835447	AY835449	AY835452	AY835749	AY835758
AY835761	AY835768	AY835769	AY835773	AY835775	AY835778	AY835779	AY835781
AY839827	AY842786	AY842808	AY842824	AY905493	AY905494	AY905495	AY905497
AY945710	AY945711	AY970946	D10112	DQ127534	DQ127537	DQ127548	DQ141341
DQ141342	DQ141343	DQ141345	DQ178989	DQ207940	DQ207942	DQ222211	DQ295192
DQ295195	DQ295196	DQ313246	DQ313249	DQ313250	DQ313252	DQ313253	DQ322227
DQ339425	DQ354118	DQ358805	DQ358808	DQ358809	DQ358810	DQ383746	DQ383749
DQ383751	DQ410068	DQ410115	DQ410205	DQ410260	DQ410292	DQ410356	DQ410403
DQ410422	DQ410465	DQ410498	DQ410526	DQ410535	DQ410553	DQ410562	DQ410596
DQ410616	DQ410636	DQ444262	DQ448819	DQ823362	DQ823363	DQ823364	DQ837381
DQ853463	DQ854716	DQ869014	DQ869015	DQ869016	DQ869017	DQ869019	DQ869020
DQ869021	DQ869022	DQ869023	DQ869024	DQ869025	DQ869026	DQ869027	DQ869028
DQ869029	DQ869030	DQ869031	DQ869032	DQ869033	DQ886031	DQ886034	DQ886036
DQ886037	DQ976407	DQ990880	EF033658	EF125659	EF210725	EF210726	EF210727
EF210729	EF210730	EF210731	EF210732	EF210734	EF210735	EF363123	EF363124
EF363126	EF363127	EF440778	EF514700	EF514701	EF514704	EF514706	EF514711
EF514712	EF531330	EF531331	EF531333	EF593190	EF593200	EF593201	EF593204
EF593208	EF593212	EF593222	EF593228	EF593232	EF593245	EF593254	EF593256
EF593260	EF593264	EF593267	EF593268	EF593269	EF593271	EF593273	EF593275
EF593282	EF593284	EF593288	EF593289	EF593290	EF593291	EF593293	EF593294
EF593295	EF593296	EF593297	EF593298	EF593299	EF593300	EF593304	EF593310
EF593311	EF593312	EF593314	EF593316	EF593318	EF637046	EF637047	EF637048
EF637049	EF637050	EF637051	EF637053	EF637054	EF637056	EU023916	EU023919
EU023921	EU023922	EU023927	EU023929	EU023932	EU023933	EU131788	EU131789
EU131790	EU131791	EU131792	EU131796	EU131797	EU131798	EU131800	EU131802
EU131803	EU131804	EU131805	EU131807	EU131808	EU131809	EU191616	EU289185
EU289186	EU289188	EU289189	EU289190	EU289191	EU289192	EU289195	EU289196
EU289197	EU289198	EU289199	EU363825	EU363827	EU363828	EU363829	EU363830
EU575474	EU575579	EU575670	EU575850	EU575927	EU576016	EU576292	EU576345
EU576383	EU576617	EU577148	EU577426	EU577469	EU577629	EU577792	EU577811
EU577862	EU577873	EU577917	EU577947	EU577989	EU578004	EU578032	EU578047
EU578061	EU578122	EU578223	EU578230	EU578260	EU578386	EU578429	EU578460
EU578539	EU578580	EU578603	EU578629	EU578667	EU604556	EU604640	EU616649
EU744052	EU744081	EU744114	EU744159	EU786676	EU786679	EU786680	EU807762
EU839596	EU839598	EU839600	EU839602	EU839605	EU839608	EU850430	FJ152546
FJ152547	FJ195086	FJ195088	FJ195089	FJ195090	FJ388890	FJ388895	FJ388898
FJ388905	FJ388910	FJ388912	FJ388914	FJ388915	FJ388916	FJ388918	FJ388920
FJ388924	FJ388927	FJ388935	FJ388936	FJ388937	FJ388955	FJ388960	FJ388964
FJ469683	FJ469684	FJ469685	FJ469686	FJ469687	FJ469688	FJ469689	FJ469690
FJ469691	FJ469692	FJ469693	FJ469694	FJ469695	FJ469696	FJ469697	FJ469698
FJ469699	FJ469701	FJ469702	FJ469703	FJ469704	FJ469705	FJ469706	FJ469707
FJ469708	FJ469709	FJ469710	FJ469711	FJ469712	FJ469713	FJ469716	FJ469718

FJ469719	FJ469721	FJ469722	FJ469723	FJ469724	FJ469725	FJ469729	FJ469730
FJ469731	FJ469732	FJ469733	FJ469737	FJ469738	FJ469739	FJ469741	FJ469742
FJ469744	FJ469745	FJ469747	FJ469748	FJ469750	FJ469752	FJ469753	FJ469754
FJ469755	FJ469757	FJ469759	FJ469760	FJ469761	FJ469762	FJ469763	FJ469764
FJ469765	FJ469766	FJ469767	FJ469768	FJ469769	FJ469770	FJ469771	FJ469772
FJ495941	FJ496085	FJ496150	FJ496157	FJ496174	FJ653127	FJ653159	FJ653196
FJ653236	FJ653258	FJ653360	FJ653390	FJ653426	FJ653437	FJ653474	FJ653478
FJ653506	FJ653547	FJ653571	FJ653573	FJ653597	FJ687543	FJ798397	FJ798416
GQ153937	K02007	K03455	L02317	L07421	L08655	M17451	M21098
M26727	M38430	M38431	M95292	U04908	U15030	U21135	U23487
U32396	U34603	U36863	U36869	U36879	U36880	U36882	U39362
U43096	U43141	U69584	U84819	U90935	Y13716	Y13719	
