# Supplementary material for: A scale-free analysis of the HIV-1 genome demonstrates multiple conserved regions of structural and functional importance
Source: PLoS Comput Biol. 2019 Sep 23;15(9):e1007345. doi: 10.1371/journal.pcbi.1007345 (PMC6791557; doi:10.1371/journal.pcbi.1007345)
Supplement: S9 Table — (PDF) [file pcbi.1007345.s040.pdf]

AB012839	AB034257	AB034258	AB034263	AB034272	AB034273	AB034279	AB034286
AB034295	AB078005	AB097870	AB221005	AB221126	AB287363	AB287364	AB287367
AB287368	AB287372	AB289588	AB289590	AB428551	AB480692	AB480694	AB480696
AB480698	AB731663	AB731667	AB873603	AB873604	AB873605	AB873606	AB873608
AB873609	AB873610	AB873612	AB873615	AB873616	AB873618	AB873620	AB873622
AB873623	AB873626	AB873627	AB873628	AB873629	AB873632	AB873635	AB873636
AB873638	AB873639	AB873641	AB873645	AB873646	AB873647	AB873653	AB873654
AB873655	AB873657	AB873661	AB873666	AB873673	AB873674	AB873677	AB873679
AB873680	AB873681	AB873683	AB873684	AB873692	AB873695	AB873697	AB873698
AB873700	AB873710	AB873712	AB873713	AB873714	AB873717	AB873718	AB873720
AB873724	AB873725	AB873730	AB873734	AB873742	AB873745	AB873751	AB873752
AB873753	AB873755	AB873756	AB873758	AB873761	AB873766	AB873769	AB873776
AB873780	AB873784	AB873792	AB873795	AB873799	AB873801	AB873805	AB873807
AB873808	AB873809	AB873815	AB873817	AB873819	AB873824	AB873826	AB873843
AB873847	AB873850	AB873851	AB873853	AB873858	AB873863	AB873872	AB873878
AB873887	AB873895	AB873899	AB873900	AF004394	AF011471	AF011474	AF011482
AF011486	AF033567	AF042101	AF047080	AF047081	AF047082	AF047085	AF049495
AF063915	AF063916	AF063919	AF063927	AF064660	AF064667	AF064676	AF069140
AF129333	AF129334	AF129335	AF129337	AF129338	AF129339	AF129340	AF129341
AF129342	AF129343	AF129345	AF129346	AF129347	AF129348	AF129349	AF129350
AF129351	AF129352	AF129353	AF129354	AF129355	AF129356	AF129357	AF129358
AF129359	AF129360	AF129361	AF129362	AF129363	AF129364	AF129365	AF129367
AF129368	AF129369	AF129370	AF129371	AF129372	AF129373	AF129374	AF129375
AF129376	AF129377	AF129378	AF129379	AF129380	AF129381	AF129382	AF129383
AF129384	AF129385	AF129386	AF129387	AF129388	AF129390	AF129391	AF129392
AF129393	AF129394	AF146728	AF147731	AF203108	AF203111	AF203116	AF203117
AF203126	AF203137	AF203141	AF203145	AF203154	AF203161	AF203165	AF203169
AF203172	AF203175	AF203180	AF203183	AF203192	AF203194	AF219672	AF219695
AF219703	AF219709	AF219714	AF219728	AF219761	AF219770	AF219771	AF219777
AF219782	AF219786	AF219796	AF219807	AF219831	AF219845	AF219851	AF219862
AF224507	AF252925	AF252937	AF272003	AF286365	AF462701	AF462704	AF462705
AF462706	AF462707	AF462714	AF462715	AF462726	AF462748	AF462749	AF462750
AF462751	AF462754	AF462758	AF462759	AF462760	AF462763	AF462764	AF462769
AF462778	AF462782	AF462788	AF462789	AF462793	AF538303	AF538304	AF538305
AF538306	AF538307	AJ271445	AJ430648	AJ430663	AJ430666	AJ430669	AJ850861
AJ850870	AJ850871	AJ850883	AJ850885	AJ850886	AJ850906	AJ850907	AJ850908
AJ850909	AJ850911	AJ850913	AY037268	AY037269	AY037270	AY037282	AY093617
AY116676	AY116694	AY116714	AY116729	AY116734	AY116748	AY116818	AY116831
AY116843	AY121441	AY121455	AY121476	AY173951	AY173952	AY173953	AY180905
AY221654	AY221675	AY221698	AY221705	AY260780	AY260782	AY260788	AY260789
AY260812	AY308760	AY314061	AY331282	AY331284	AY331285	AY331287	AY331289
AY331292	AY331294	AY331296	AY332237	AY352275	AY363365	AY444308	AY444310
AY444311	AY444312	AY444313	AY444317	AY560107	AY560108	AY560110	AY561237
AY561239	AY561240	AY561245	AY586543	AY701281	AY701282	AY713408	AY713410
AY739034	AY739035	AY739036	AY739040	AY739041	AY739046	AY779552	AY779553
AY779559	AY781127	AY786611	AY786651	AY786773	AY795904	AY795905	AY818644
AY819715	AY835749	AY835753	AY835761	AY835763	AY835768	AY835773	AY835778
AY835779	AY856759	AY856762	AY856765	AY856766	AY856767	AY856768	AY856769
AY856770	AY856771	AY856773	AY856776	AY857022	AY857144	AY899354	AY899356
AY899359	AY899382	AY905389	AY905390	AY905392	AY905394	AY905395	AY905396
AY905400	D10112	DQ007901	DQ007903	DQ121678	DQ121681	DQ121756	DQ121762
DQ121801	DQ121881	DQ121912	DQ121927	DQ121957	DQ121963	DQ121974	DQ121984
DQ121991	DQ121999	DQ122019	DQ122028	DQ122032	DQ122045	DQ122061	DQ122075
DQ122088	DQ122095	DQ122099	DQ122106	DQ122111	DQ122112	DQ122115	DQ122119
DQ122122	DQ127535	DQ127537	DQ127540	DQ127548	DQ207940	DQ207942	DQ242535
DQ322227	DQ322239	DQ354112	DQ354116	DQ354118	DQ354119	DQ357219	DQ357221
DQ358805	DQ358808	DQ358809	DQ358810	DQ383746	DQ383748	DQ383749	DQ383750

DQ383751	DQ396398	DQ400962	DQ400966	DQ400968	DQ400970	DQ484085	DQ484102
DQ484108	DQ484287	DQ484357	DQ484423	DQ484434	DQ484451	DQ484523	DQ484574
DQ484580	DQ484582	DQ484583	DQ484587	DQ484593	DQ484596	DQ484600	DQ484614
DQ484625	DQ484636	DQ484647	DQ484699	DQ484706	DQ484711	DQ484714	DQ484718
DQ484719	DQ484733	DQ484741	DQ484795	DQ484815	DQ484818	DQ484831	DQ484833
DQ484834	DQ484919	DQ484920	DQ484927	DQ484929	DQ484934	DQ484994	DQ484997
DQ485003	DQ485011	DQ485017	DQ485020	DQ485076	DQ485078	DQ485084	DQ485087
DQ485094	DQ485095	DQ485097	DQ487190	DQ659700	DQ659701	DQ659703	DQ659710
DQ659725	DQ659731	DQ659733	DQ659737	DQ823362	DQ823364	DQ837381	DQ854716
DQ886031	DQ886032	DQ886033	DQ886034	DQ886035	DQ886036	DQ886037	DQ990880
DQ996257	DQ996259	DQ996267	EF125654	EF175212	EF363123	EF363124	EF363126
EF363127	EF462995	EF462996	EF462998	EF462999	EF463000	EF463002	EF463003
EF514697	EF514699	EF514700	EF514701	EF514704	EF514706	EF514707	EF514711
EF514712	EF637046	EF637047	EF637048	EF637049	EF637050	EF637051	EF637053
EF637054	EF637056	EF637057	EF694037	EU312170	EU312171	EU312176	EU327409
EU327415	EU327420	EU327428	EU327452	EU327490	EU410053	EU432531	EU432532
EU432533	EU432534	EU432538	EU432539	EU432540	EU432542	EU432543	EU432544
EU432545	EU616642	EU786674	EU786676	EU786678	EU786679	EU786680	EU807781
FJ195086	FJ195088	FJ195089	FJ195090	FJ195091	FJ201781	FJ201791	FJ201812
FJ201857	FJ201870	FJ201895	FJ201920	FJ423554	FJ423555	FJ423560	FJ423562
FJ423568	FJ423569	FJ460499	FJ460500	FJ460501	FJ469682	FJ469683	FJ469684
FJ469685	FJ469686	FJ469688	FJ469689	FJ469690	FJ469691	FJ469692	FJ469693
FJ469694	FJ469695	FJ469696	FJ469697	FJ469699	FJ469700	FJ469701	FJ469702
FJ469703	FJ469704	FJ469705	FJ469706	FJ469707	FJ469708	FJ469710	FJ469711
FJ469712	FJ469713	FJ469714	FJ469715	FJ469716	FJ469717	FJ469718	FJ469719
FJ469721	FJ469722	FJ469723	FJ469725	FJ469726	FJ469727	FJ469728	FJ469730
FJ469731	FJ469735	FJ469737	FJ469738	FJ469739	FJ469740	FJ469741	FJ469742
FJ469743	FJ469745	FJ469747	FJ469749	FJ469750	FJ469751	FJ469752	FJ469753
FJ469755	FJ469756	FJ469758	FJ469759	FJ469760	FJ469761	FJ469763	FJ469764
FJ469766	FJ469767	FJ469768	FJ469771	FJ469772	FJ495818	FJ495941	FJ496000
FJ496078	FJ496081	FJ496145	FJ496151	FJ496169	FJ647145	FJ798362	FJ798370
FJ798391	FJ798414	FJ798482	FJ798513	FJ798530	FJ798566	GQ256631	GQ372332
GQ372373	GQ372378	GQ372392	GQ372402	GQ372410	GQ372424	GQ372446	GQ372472
GQ372477	GQ372518	GQ372540	GQ372567	GQ372585	GQ372589	GQ372605	GQ372613
GQ372628	GQ372636	GQ372655	GQ372660	GQ372664	GQ372674	GQ372682	GQ372688
GQ372689	GQ372691	GQ372693	GQ372695	GQ372698	GQ372707	GQ372713	GQ372749
GQ372751	GQ372774	GQ372790	JF283660	JF283667	JF283678	JX140659	JX171201
JX171215	JX171217	JX171218	JX171222	JX171227	JX171229	JX171230	JX171231
JX171232	JX171233	JX171234	JX171237	JX171238	JX171239	JX440930	JX440934
JX440935	JX440937	JX440939	JX440941	JX440945	JX440946	JX440948	JX440957
JX440958	JX440961	JX440963	JX440964	JX440965	JX440967	JX440968	JX440969
JX972342	K02007	KC312386	KC312435	KC312470	KC312583	KC473824	KC473825
KC473826	KC473827	KC473828	KC473829	KC473830	KC473831	KC473832	KC473833
KC473835	KC473841	KC473842	KC473846	KC596066	KC596067	KC596069	KC797171
KC797225	KC899011	KC906900	KC906901	KC906902	KC906903	KC906907	KC906908
KC906910	KC906912	KC906916	KC906917	KC906918	KC906920	KC906921	KC906922
KC906923	KC906925	KC906926	KC906927	KC906928	KC906930	KC906931	KC906932
KC906933	KC906934	KC906936	KC906939	KC906940	KC906941	KC906942	KC906943
KC906945	KC906947	KC906948	KC906949	KC906951	KC906953	KC906954	KC906955
KC906956	KC906957	KC906958	KC906959	KC906960	KC906962	KC906963	KC906964
KC906965	KC906966	KC906967	KC906968	KC906969	KC906970	KC906971	KC906972
KC906973	KC906974	KC906979	KC906981	KC906982	KC906983	KC906984	KC906987
KC906989	KC906990	KC935958	KC935959	KF384800	KF384804	KF384808	KF384811
KF384812	KF384813	KF384814	KF526228	KF526265	KF526312	KF526323	KF561441
KF561442	KF716494	KF716495	KF716497	L07425	L15476	L15483	L15492
L15500	L15509	L15515	L15518	M17451	M21098	M26727	M38429
M58206	M84627	M84630	M84631	M93258	U03375	U16863	U16869

U16875	U16893	U16898	U16909	U16917	U16921	U16934	U23487
U24455	U26074	U26098	U26112	U26119	U26141	U34604	U39362
U43096	U43108	U44450	U44465	U44468	U52491	U61833	U66543
U71182	U73339	U73370	X63041	X63044	X64747	Y15122	Z98019
Z98022	Z98024	Z98027	Z98029	Z98030	Z98032	Z98034	
