# Supplementary material for: A scale-free analysis of the HIV-1 genome demonstrates multiple conserved regions of structural and functional importance
Source: PLoS Comput Biol. 2019 Sep 23;15(9):e1007345. doi: 10.1371/journal.pcbi.1007345 (PMC6791557; doi:10.1371/journal.pcbi.1007345)
Supplement: S10 Table — (PDF) [file pcbi.1007345.s041.pdf]

AB097870	AB221005	AB221125	AB286955	AB287363	AB287364	AB287366	AB287368
AB287370	AB289588	AB289590	AB428554	AB480692	AB480694	AB480696	AB480698
AB564745	AB565478	AB565496	AB565497	AB565502	AB604946	AB604948	AB641836
AB731663	AB731667	AB731669	AF004394	AF042100	AF042101	AF042104	AF042105
AF069140	AF146728	AF286365	AF538302	AF538305	AF538306	AF538307	AJ271445
AY037268	AY037269	AY037270	AY037282	AY173952	AY173953	AY173959	AY173960
AY180905	AY314056	AY331284	AY331296	AY332237	AY352275	AY560107	AY560108
AY560109	AY560110	AY561237	AY713408	AY713410	AY779553	AY781127	AY795904
AY795905	AY835749	AY835753	AY835758	AY835761	AY835763	AY835768	AY835769
AY835773	AY835774	AY835775	AY835777	AY835779	AY835781	AY839827	AY945710
AY945711	D10112	DQ007903	DQ127534	DQ127537	DQ127549	DQ207942	DQ207943
DQ295192	DQ358805	DQ358808	DQ358809	DQ358810	DQ383748	DQ383749	DQ396398
DQ823362	DQ823363	DQ823364	DQ837381	DQ853463	DQ854716	DQ886031	DQ886032
DQ886033	DQ886034	DQ886035	DQ886036	DQ886037	DQ990880	EF175212	EF363123
EF363124	EF363126	EF363127	EF514701	EF514704	EF637046	EF637047	EF637048
EF637049	EF637050	EF637051	EF637053	EF637054	EF637056	EF637057	EF694037
EU547186	EU616649	EU786679	FJ195086	FJ195088	FJ195089	FJ195090	FJ195091
FJ388890	FJ388895	FJ388898	FJ388899	FJ388904	FJ388905	FJ388911	FJ388914
FJ388915	FJ388916	FJ388919	FJ388924	FJ388927	FJ388930	FJ388931	FJ388933
FJ388934	FJ388935	FJ388936	FJ388940	FJ388947	FJ388955	FJ388957	FJ388958
FJ388960	FJ388962	FJ388963	FJ388964	FJ388965	FJ469682	FJ469683	FJ469684
FJ469685	FJ469686	FJ469687	FJ469688	FJ469689	FJ469690	FJ469691	FJ469692
FJ469693	FJ469694	FJ469695	FJ469696	FJ469697	FJ469698	FJ469699	FJ469700
FJ469701	FJ469702	FJ469703	FJ469704	FJ469705	FJ469706	FJ469707	FJ469708
FJ469709	FJ469710	FJ469711	FJ469712	FJ469713	FJ469714	FJ469715	FJ469716
FJ469717	FJ469718	FJ469719	FJ469721	FJ469722	FJ469723	FJ469725	FJ469726
FJ469727	FJ469728	FJ469729	FJ469730	FJ469731	FJ469732	FJ469734	FJ469735
FJ469737	FJ469738	FJ469739	FJ469740	FJ469741	FJ469742	FJ469743	FJ469744
FJ469745	FJ469747	FJ469748	FJ469749	FJ469750	FJ469752	FJ469753	FJ469755
FJ469756	FJ469757	FJ469758	FJ469759	FJ469760	FJ469761	FJ469763	FJ469764
FJ469766	FJ469767	FJ469768	FJ469769	FJ469770	FJ469771	FJ469772	FJ495818
FJ495941	FJ496000	FJ496081	FJ670531	FJ694790	GU177863	GU733713	JF320003
JF320008	JF320036	JF320038	JF320043	JF320048	JF320053	JF320054	JF320059
JF320097	JF320126	JF320145	JF320150	JF320151	JF320160	JF320169	JF320185
JF320189	JF320208	JF320215	JF320226	JF320244	JF320263	JF320356	JF320361
JF320363	JF320427	JF320467	JF320484	JF320526	JF320530	JF320563	JF320613
JF320615	JF683765	JF683797	JF683804	JF683805	JF683807	JF689852	JF689857
JF689859	JF689860	JF689862	JF689865	JF689867	JF689872	JF689877	JF689883
JF689886	JF689890	JF689895	JF932469	JF932470	JF932471	JF932472	JF932473
JF932474	JF932475	JF932476	JF932477	JF932479	JF932480	JF932481	JF932483
JF932484	JF932485	JF932486	JF932487	JF932488	JF932489	JF932490	JF932491
JF932492	JF932493	JF932494	JF932495	JF932496	JF932497	JF932498	JF932499
JF932500	JN024100	JN024210	JN024303	JN024344	JN024363	JN024428	JN235958
JN248329	JN248333	JN248337	JN248346	JN248347	JN248353	JN248354	JN251901
JN397362	JN599165	JN692432	JN692433	JN692435	JN692439	JN692440	JN692443
JN692444	JN692445	JN692447	JN692450	JN692451	JN692452	JN692453	JN692454
JN692455	JN692457	JN692460	JN692461	JN692462	JN692463	JN692465	JN692467
JN692468	JN692470	JN692471	JN692473	JN692474	JN692475	JN692479	JN692480
JN944897	JN944911	JN944917	JN944930	JQ316126	JQ316127	JQ316128	JQ316130
JQ316131	JQ316132	JQ316133	JQ316134	JQ316135	JQ341411	JQ403019	JQ403021
JQ403022	JQ403023	JQ403024	JQ403025	JQ403026	JQ403029	JQ403031	JQ403035
JQ403037	JQ403042	JQ403045	JQ403046	JQ403047	JQ403048	JQ403059	JQ403060
JQ403061	JQ403062	JQ403064	JQ403066	JQ403067	JQ403068	JQ403069	JQ403070
JQ403071	JQ403074	JQ403075	JQ403078	JQ403079	JQ403082	JQ403083	JQ403085
JQ403086	JQ403087	JQ403088	JQ403089	JQ403091	JQ403092	JQ403093	JQ403094
JQ403095	JQ403096	JQ403097	JQ403098	JQ403100	JQ403102	JQ403104	JQ403105
JQ403106	JQ403107	JQ416158	JQ429433	JX140652	JX140654	JX140657	JX140658

JX500708	JX500709	JX503075	JX960597	JX960599	K02007	K03455	KC473824
KC473825	KC473826	KC473827	KC473828	KC473829	KC473831	KC473833	KC473835
KC473842	KC473846	KC596067	KC596069	KC797171	KC797225	KC899011	KC935957
KF384798	KF384799	KF384800	KF384801	KF384802	KF384803	KF384804	KF384805
KF384806	KF384807	KF384808	KF384810	KF384811	KF384812	KF384813	KF384814
KF526141	KF526228	KF526265	KF526312	KF526323	KF561442	KF716494	KF716495
KF716496	KF716497	KF716498	KF990605	KF990608	KJ140250	KJ140251	KJ140255
KJ140261	KJ140263	KJ140264	KJ140265	KJ140266	KJ769147	KJ849784	KJ849807
KJ849808	KJ849811	KJ849814	KJ948656	KJ948660	KP109511	KP109512	KP109514
KP109515	KP109518	KP411823	KP411824	KP411825	KP411829	KT200348	KT200349
KT200350	KT200351	KT200352	KT200353	KT200354	KT200355	KT200356	KT200357
KT200358	KT284371	L02317	M17449	M17451	M26727	M38431	M93258
U21135	U23487	U34604	U39362	U43096	U43141	U63632	U69591
U71182							
