# Supplementary material for: A scale-free analysis of the HIV-1 genome demonstrates multiple conserved regions of structural and functional importance
Source: PLoS Comput Biol. 2019 Sep 23;15(9):e1007345. doi: 10.1371/journal.pcbi.1007345 (PMC6791557; doi:10.1371/journal.pcbi.1007345)
Supplement: S11 Table — (PDF) [file pcbi.1007345.s042.pdf]

AB098331	AB098333	AB253421	AB253429	AB287377	AB287379	AB485632	AF004885
AF069671	AF286237	AF286247	AF286247	AF361873	AM000053	AM000053	AM000055
AM000055	AY322185	AY322190	AY322193	AY772953	AY772954	AY772955	AY772958
AY772985	DQ396400	EF122512	EU242119	EU446023	EU446023	EU673416	EU673418
EU673439	FJ388892	FJ388903	FJ388925	FJ388932	FJ388942	FJ623475	FJ623477
FJ623478	FJ623479	FJ623480	FJ623481	FJ623482	FJ623483	FJ623487	FJ853508
FJ853511	FJ853518	FJ853537	FJ853571	FJ853583	FJ853589	FJ864679	GQ429817
GQ429905	GQ429911	GQ429918	GQ429926	GQ429942	GQ430033	GQ430063	GQ430176
GQ430216	GQ430223	GQ430293	GQ430383	GQ430447	GQ430614	GQ430627	GQ430674
GQ430676	GQ430706	GQ430768	GQ430783	GQ430800	GQ430863	GQ430867	GQ431002
GQ431005	GQ431019	GQ431220	GQ431224	GQ431310	GQ431316	GQ431357	GQ431370
GQ431425	GQ431662	GQ431663	GQ431672	GQ431676	GQ431679	GQ431692	GQ431724
GQ431783	GQ431844	GQ431845	GQ431939	GQ431965	GQ431986	GQ432033	GQ432034
GQ432094	GQ432180	GQ432382	GQ432385	GQ432411	GQ432440	GQ432476	GQ432503
GQ432562	GQ432576	GQ432594	GQ432657	GQ432683	GQ432715	GU332524	GU367443
JF683760	JF683767	JF683779	JF683783	JF683798	JQ292893	JQ403028	JX236669
JX236671	JX236676	JX236677	JX236678	JX244900	JX244906	JX500694	JX500695
JX500696							
