# Supplementary material for: A scale-free analysis of the HIV-1 genome demonstrates multiple conserved regions of structural and functional importance
Source: PLoS Comput Biol. 2019 Sep 23;15(9):e1007345. doi: 10.1371/journal.pcbi.1007345 (PMC6791557; doi:10.1371/journal.pcbi.1007345)
Supplement: S12 Table — (PDF) [file pcbi.1007345.s043.pdf]

AB098331	AB098333	AB253421	AB253429	AB287377	AB287379	AB485632	AF004885
AF069669	AF069670	AF069671	AF286237	AF286240	AF361872	AF413987	AF457052
AF457053	AF457055	AF457063	AF457065	AF457066	AF457067	AF457069	AF457070
AF457075	AF457077	AF457079	AF457080	AF457081	AF457083	AF457084	AF457086
AF484478	AF484493	AF484508	AF484509	AF539405	AM000053	AM000054	AM000055
AY253305	AY253314	AY322184	AY322190	AY322193	AY521630	AY521631	AY713406
DQ396400	FJ388892	FJ388893	FJ388903	FJ388909	FJ388925	FJ388942	FJ623475
FJ623476	FJ623478	FJ623479	FJ623480	FJ623481	FJ623482	FJ623483	FJ623485
FJ623486	FJ623487	FJ647146	FJ647148	FJ670523	GU201516	M62320	
