# Supplementary material for: A scale-free analysis of the HIV-1 genome demonstrates multiple conserved regions of structural and functional importance
Source: PLoS Comput Biol. 2019 Sep 23;15(9):e1007345. doi: 10.1371/journal.pcbi.1007345 (PMC6791557; doi:10.1371/journal.pcbi.1007345)
Supplement: S13 Table — (PDF) [file pcbi.1007345.s044.pdf]

AB098330	AB098332	AB253421	AB253429	AB253429	AB287377	AB287379	AF004885
AF069670	AF069671	AF069673	AF107771	AF200477	AF200477	AF233689	AF233689
AF286237	AF286238	AF286240	AF286241	AF361872	AF361873	AF413987	AF457052
AF457053	AF457063	AF457066	AF457067	AF457068	AF457069	AF457070	AF457075
AF457077	AF457079	AF457080	AF457081	AF457083	AF457084	AF457086	AF457089
AF484478	AF484493	AF484507	AF484509	AF484512	AF539405	AM000053	AM000053
AM000054	AM000054	AM000055	AM000055	AY253305	AY253314	AY322193	AY521630
AY521630	AY521631	AY521631	AY713406	AY905588	AY905605	DQ072819	DQ396400
DQ871534	DQ871534	EU110094	EU110095	EU542007	EU839407	EU839412	EU839413
EU839414	EU839415	EU839417	EU861977	FJ388892	FJ388893	FJ388903	FJ388909
FJ388925	FJ388938	FJ388942	FJ623475	FJ623476	FJ623477	FJ623478	FJ623479
FJ623481	FJ623483	FJ623485	FJ623486	FJ623487	FJ623488	FJ647148	FJ670519
FJ882080	GU201516	HM027824	JF683737	JF683748	JF683759	JF683760	JF683763
JF683767	JF683779	JF683782	JF683783	JF683789	JF683798	JF957866	JF957884
JQ248225	JQ292891	JQ292896	JQ292897	JQ292900	JQ403028	JX236669	JX236671
JX236676	JX236677	JX236678	JX500694	JX500695	K03455	KF716472	KF716474
KF716475	KF716478	KF716486	KF716491	KF716492	KF859745	KP109490	KP718918
KP718928	KT022360	KT022361	KT022363	KT022364	KT022365	KT022367	KT022368
KT022369	KT022370	KT022372	KT022373	KT022374	KT022375	KT022376	KT022377
KT022378	KT022380	KT022381	KT022382	KT022383	KT152841	KT152842	KT152844
KT152846	KT183312	M62320	M62320	X91352	X91352	X91354	X91354
X91359	X91359	X91364	X91364	X91368	X91368	X91375	X91375
X91376	X91376	X91378	X91378	Z30637	Z30637		
