# Supplementary material for: A scale-free analysis of the HIV-1 genome demonstrates multiple conserved regions of structural and functional importance
Source: PLoS Comput Biol. 2019 Sep 23;15(9):e1007345. doi: 10.1371/journal.pcbi.1007345 (PMC6791557; doi:10.1371/journal.pcbi.1007345)
Supplement: S14 Table — (PDF) [file pcbi.1007345.s045.pdf]

AB098330	AB098332	AB253421	AB253429	AB287379	AB485632	AF004885	AF069669
AF069670	AF069671	AF069673	AF107771	AF286237	AF286238	AF286240	AF361872
AF361873	AF413973	AF457052	AF457053	AF457063	AF457065	AF457066	AF457067
AF457068	AF457069	AF457079	AF457081	AF457083	AF457084	AF457086	AF457089
AF484478	AF484493	AF484507	AF484509	AF484512	AF539405	AM000053	AM000053
AM000054	AM000054	AM000055	AM000055	AY253305	AY253314	AY322184	AY322193
AY521630	AY521630	AY521631	AY521631	AY713406	DQ396400	EF545108	EU110094
EU110095	EU110097	EU861977	FJ388893	FJ388909	FJ388925	FJ388932	FJ388938
FJ623475	FJ623476	FJ623477	FJ623478	FJ623479	FJ623480	FJ623481	FJ623482
FJ623483	FJ623485	FJ623486	FJ623487	FJ623488	FJ647148	FJ670519	FJ670523
GU201516	JF683737	JF683748	JF683759	JF683760	JF683763	JF683767	JF683779
JF683782	JF683783	JF683789	JF683798	JQ292891	JQ292893	JQ292894	JQ292895
JQ292896	JQ292897	JQ292900	JQ403028	JX236669	JX236671	JX236676	JX236677
JX236678	JX500694	JX500695	JX500696	KF716472	KF716474	KF716475	KF716478
KF716486	KF716491	KF716492	KF859745	KP109490	KP718918	KP718928	KT022360
KT022361	KT022363	KT022364	KT022365	KT022367	KT022368	KT022369	KT022370
KT022372	KT022373	KT022374	KT022375	KT022376	KT022377	KT022378	KT022380
KT022381	KT022382	KT022383	KT152839	KT152841	KT152842	KT152844	KT152846
KT183312							
