# Supplementary material for: A scale-free analysis of the HIV-1 genome demonstrates multiple conserved regions of structural and functional importance
Source: PLoS Comput Biol. 2019 Sep 23;15(9):e1007345. doi: 10.1371/journal.pcbi.1007345 (PMC6791557; doi:10.1371/journal.pcbi.1007345)
Supplement: S15 Table — (PDF) [file pcbi.1007345.s046.pdf]

AB098330	AB098332	AB253429	AB287377	AB287379	AF004885	AF069669	AF069670
AF069671	AF069673	AF107771	AF286237	AF286238	AF286241	AF361872	AF361873
AF413987	AF457052	AF457053	AF457055	AF457063	AF457065	AF457066	AF457069
AF457070	AF457075	AF457077	AF457079	AF457080	AF457083	AF457084	AF457086
AF457089	AF484493	AF484507	AF484508	AF484509	AF484512	AF539405	AM000053
AM000054	AM000055	AY253305	AY253314	AY322184	AY322190	AY322193	AY521630
AY521631	AY713406	AY829210	DQ396400	DQ823357	DQ823367	EU110088	EU110092
EU861977	FJ388892	FJ388893	FJ388903	FJ388906	FJ388909	FJ388925	FJ388932
FJ388938	FJ388951	FJ647148	KJ948658	L22951	L22957	M62320	
