# Supplementary material for: A scale-free analysis of the HIV-1 genome demonstrates multiple conserved regions of structural and functional importance
Source: PLoS Comput Biol. 2019 Sep 23;15(9):e1007345. doi: 10.1371/journal.pcbi.1007345 (PMC6791557; doi:10.1371/journal.pcbi.1007345)
Supplement: S16 Table — (PDF) [file pcbi.1007345.s047.pdf]

AB098330	AB098332	AB253421	AB253429	AB253429	AB287377	AB287379	AF004885
AF069669	AF069670	AF069671	AF069673	AF082486	AF107771	AF219261	AF286238
AF286241	AF361872	AF361873	AF457052	AF457053	AF457063	AF457065	AF457066
AF457068	AF457069	AF457070	AF457075	AF457077	AF457079	AF457080	AF457081
AF457083	AF457084	AF457086	AF457089	AF484493	AF484507	AF484508	AF484509
AF484512	AF539405	AM000053	AM000053	AM000054	AM000054	AM000055	AM000055
AY253305	AY253314	AY322193	AY521631	AY521631	AY713406	AY829205	DQ396400
DQ823366	DQ823367	EU110092	EU110094	EU861977	FJ388892	FJ388893	FJ388903
FJ388906	FJ388909	FJ388925	FJ388932	FJ388938	FJ388942	FJ388950	FJ388951
FJ443124	FJ623475	FJ623476	FJ623477	FJ623478	FJ623479	FJ623481	FJ623482
FJ623485	FJ623487	FJ623488	FJ647148	FJ670523	FJ866111	FJ866115	FJ866117
FJ866118	FJ866119	FJ866121	GU201516	HM027824	HM027846	JF683737	JF683748
JF683763	JF683779	JF683782	JF683783	JF683789	JF683798	JQ292891	JQ292893
JQ292895	JQ292897	JQ292898	JQ292900	JQ403028	JX236669	JX236671	JX236676
JX236677	JX236678	JX500694	JX500695	JX500696	K03455	KF716472	KF716474
KF716475	KF716478	KF716486	KF716491	KF716492	KF859745	KJ948658	KP109490
KP718918	KP718928	KT022360	KT022361	KT022363	KT022364	KT022365	KT022367
KT022368	KT022369	KT022370	KT022372	KT022373	KT022374	KT022375	KT022376
KT022377	KT022378	KT022380	KT022381	KT022382	KT022383	KT152842	KT152844
KT152846	KT183312	L22951	L22957	M62320	M62320		
