# Supplementary material for: A scale-free analysis of the HIV-1 genome demonstrates multiple conserved regions of structural and functional importance
Source: PLoS Comput Biol. 2019 Sep 23;15(9):e1007345. doi: 10.1371/journal.pcbi.1007345 (PMC6791557; doi:10.1371/journal.pcbi.1007345)
Supplement: S17 Table — (PDF) [file pcbi.1007345.s048.pdf]

AB098330	AB098332	AB253421	AB253429	AB253429	AB287377	AB287379	AF004885
AF069671	AF069673	AF082486	AF107771	AF219261	AF219265	AF286237	AF286238
AF286241	AF410441	AF457055	AF457063	AF457065	AF457068	AF457069	AF457079
AF457081	AF457083	AF457084	AF484478	AF484493	AF484507	AF484508	AF484509
AM000053	AM000053	AM000054	AM000054	AM000055	AM000055	AY253305	AY322184
AY322190	AY322193	AY521630	AY521630	AY521631	AY521631	AY713406	DQ396400
DQ676873	DQ823357	EF545108	EU110094	EU836349	EU836364	EU836378	EU836404
EU836406	EU836438	EU836442	EU836463	EU836467	EU836476	EU836484	EU836515
EU836524	EU836528	EU836534	EU836544	EU836553	EU861977	EU875267	FJ388893
FJ388903	FJ388909	FJ388925	FJ388932	FJ388950	FJ623475	FJ623477	FJ623478
FJ623479	FJ623481	FJ623482	FJ623484	FJ623485	FJ623486	FJ623488	FJ647148
FJ670523	FJ864679	FJ866111	FJ866115	FJ866117	GU201516	HM027823	HM027846
HQ834965	HQ834967	HQ834967	JQ292891	JQ292897	JQ292898	JQ292900	JQ403028
JQ966758	JX236669	JX236671	JX236676	JX236677	JX236678	JX500694	JX500695
JX500696	KC332898	KF716472	KF716474	KF716475	KF716478	KF716486	KF716491
KF716492	KF859745	KJ948658	KP109490	KP718918	KP718928	KT012691	KT022360
KT022361	KT022363	KT022364	KT022365	KT022367	KT022368	KT022369	KT022370
KT022372	KT022373	KT022374	KT022375	KT022376	KT022377	KT022378	KT022380
KT022381	KT022382	KT022383	KT152842	KT152844	KT183312	L22957	
