# Supplementary material for: A scale-free analysis of the HIV-1 genome demonstrates multiple conserved regions of structural and functional importance
Source: PLoS Comput Biol. 2019 Sep 23;15(9):e1007345. doi: 10.1371/journal.pcbi.1007345 (PMC6791557; doi:10.1371/journal.pcbi.1007345)
Supplement: S18 Table — (PDF) [file pcbi.1007345.s049.pdf]

AB098330	AB253421	AB253429	AB287377	AB287379	AF004885	AF069670	AF069671
AF107771	AF219261	AF286237	AF286238	AF286241	AF361872	AF361873	AF407157
AF407162	AF457053	AF457055	AF457070	AF457075	AF457080	AF457081	AF457084
AF457086	AF484493	AF484507	AF484509	AF539405	AM000053	AM000054	AM279348
AY253305	AY253314	AY322184	AY322190	AY322193	AY494973	AY521629	AY521630
AY521631	AY713406	AY736809	AY736812	AY736814	AY736815	AY736816	DQ208448
DQ208458	DQ208467	DQ208501	DQ823357	EU191615	EU852954	EU852958	EU853030
EU853062	FJ388892	FJ388894	FJ388903	FJ388925	FJ388938	FJ388942	FJ396018
FJ647148	FJ864679	FJ866112	FJ866117	FJ866118	FJ866120	FJ866121	FM165628
FM165645	L07082	L22943	L22957	Y13717	Y13718		
