# Supplementary material for: A scale-free analysis of the HIV-1 genome demonstrates multiple conserved regions of structural and functional importance
Source: PLoS Comput Biol. 2019 Sep 23;15(9):e1007345. doi: 10.1371/journal.pcbi.1007345 (PMC6791557; doi:10.1371/journal.pcbi.1007345)
Supplement: S19 Table — (PDF) [file pcbi.1007345.s050.pdf]

AB098331	AB098333	AB253421	AB253429	AB287377	AB287379	AB485632	AF004885
AF069670	AF069671	AF069673	AF107771	AF286237	AF286238	AF361872	AF361873
AF425872	AF425880	AF425882	AF425885	AF425891	AF425897	AF457052	AF457053
AF457055	AF457063	AF457066	AF457067	AF457068	AF457069	AF457070	AF457075
AF457077	AF457079	AF457080	AF457081	AF457083	AF457084	AF457086	AF462696
AF462724	AF484478	AF484493	AF484507	AF484508	AF484509	AF484512	AF539405
AJ850901	AM000053	AM000054	AM000055	AY121459	AY121463	AY253314	AY265064
AY265067	AY265071	AY265086	AY265103	AY322184	AY322190	AY322193	AY521629
AY521630	AY521631	AY584800	AY713406	AY803410	AY803411	AY803418	AY803429
AY803432	AY803434	AY803437	AY803438	AY803444	AY803447	AY803449	AY803450
AY803453	AY803454	AY803457	AY803459	AY887102	AY899370	AY905393	AY905399
DQ122025	DQ396400	DQ659684	DQ659693	DQ823366	EF186119	EF186130	EF186180
EF186200	EU110092	EU110094	FJ647148	FJ864679	KC906806	KC906807	KC906809
KC906811	KC906812	KC906813	KC906814	KC906817	KC906818	KC906819	KC906820
KC906821	KC906822	KC906824	KC906825	KC906826	KC906827	KC906828	KC906829
KC906830	KC906831	KC906832	KC906833	KC906834	KC906835	KC906836	KC906838
KC906840	KC906841	KC906842	KC906844	KC906845	KC906846	KC906847	KC906848
KC906849	KC906850	KC906851	KC906853	KC906854	KC906855	KC906856	KC906857
KC906858	KC906861	KC906862	KC906864	KC906866	KC906867	KC906868	KC906869
KC906871	KC906872	KC906873	KC906874	KC906875	KC906876	KC906878	KC906879
KC906880	KC906883	KC906884	KC906885	KC906886	KC906887	KC906888	KC906889
KC906890	KC906891	KC906893	KC906894	KC906895	KC906896	KC906897	KC906898
KC906899	KF716474	KF716475	KF716478	KF716486	KF716491	KF716492	KF859745
M62320							
