# Supplementary material for: A scale-free analysis of the HIV-1 genome demonstrates multiple conserved regions of structural and functional importance
Source: PLoS Comput Biol. 2019 Sep 23;15(9):e1007345. doi: 10.1371/journal.pcbi.1007345 (PMC6791557; doi:10.1371/journal.pcbi.1007345)
Supplement: S20 Table — (PDF) [file pcbi.1007345.s051.pdf]

AB098331	AB098333	AB253421	AB253429	AB253429	AB287376	AB287379	AB485632
AF004885	AF069670	AF069671	AF069673	AF107771	AF286237	AF286238	AF361873
AF413987	AF457052	AF457053	AF457055	AF457063	AF457066	AF457069	AF457075
AF457077	AF457079	AF457080	AF457081	AF457083	AF457084	AF457086	AF484493
AF484507	AF484508	AF484509	AF484512	AF539405	AM000053	AM000054	AY253305
AY253306	AY253314	AY322185	AY322190	AY322193	AY521629	AY521630	AY521631
AY713406	DQ396400	DQ823366	FJ388893	FJ388903	FJ388906	FJ388909	FJ388925
FJ388938	FJ388942	FJ388950	FJ388951	FJ623475	FJ623477	FJ623478	FJ623479
FJ623481	FJ623486	FJ623487	GU201516	JF683767	JF683779	JF683783	JQ403028
JX236669	JX236671	JX236676	JX236677	JX236678	JX500695	KF716472	KF716474
KF716475	KF716478	KF716486	KF716491	KF716492	KF859745	KP718918	KP718928
KT022361	KT022363	KT022364	KT022367	KT022369	KT022373	KT022374	KT022375
KT022376	KT022377	KT022378	KT022380	KT022381	KT022382	KT022383	KT152844
KT152846	KT183312	M62320					
