# Supplementary material for: A scale-free analysis of the HIV-1 genome demonstrates multiple conserved regions of structural and functional importance
Source: PLoS Comput Biol. 2019 Sep 23;15(9):e1007345. doi: 10.1371/journal.pcbi.1007345 (PMC6791557; doi:10.1371/journal.pcbi.1007345)
Supplement: S21 Table — See main text for further discussion. (PDF) [file pcbi.1007345.s052.pdf]

Gene	Order found within gene	HXB2 location	NL4-3 location	Z	p	Comment
gag	2	1549–1635	1549–1635	3.76	0.0083	Matches non-significant conserved region in B sequences
	1	2251–2277	2251–2277	3.63	0.0153	Matches non-significant conserved region in B sequences
pol	1	4749–4982	4749–4982	5.55	< 0.0001	Found in B sequences
tat	1	5951–5986	5950–5985	3.61	0.0007	Found in B sequences
vpu	1	6194–6226	6190–6222	4.05	0.0003	Found in B sequences
env	4	6339–6599	6335–6595	3.62	0.0439	Found in B sequences
	5	6840–6896	6830–6882	3.80	0.0175	Found in B sequences
	3	6945–7022	6935–7012	4.06	0.0040	Found in B sequences
	1	7656–8042	7646–8032	6.88	< 0.0001	Found in B sequences
	2	8265–8591	8255–8581	4.75	0.0003	Found in B sequences
nef	1	9052–9096	9042–9086	4.17	0.0003	Found in B sequences
