# Supplementary material for: A scale-free analysis of the HIV-1 genome demonstrates multiple conserved regions of structural and functional importance
Source: PLoS Comput Biol. 2019 Sep 23;15(9):e1007345. doi: 10.1371/journal.pcbi.1007345 (PMC6791557; doi:10.1371/journal.pcbi.1007345)
Supplement: S22 Table — Not all of the sequences whose accession numbers are listed in S10 Table contain a fully sequenced 5′ header; only those listed here are therefore used in the production of S16–S22 Figs. (PDF) [file pcbi.1007345.s053.pdf]

AB221005	AB221125	AB286955	AB287363	AB287364	AB287366	AB287368	AB287370
AB289588	AB289590	AB480692	AB480694	AB480696	AB480698	AB564745	AB565478
AB565496	AB565497	AB565502	AB604946	AB604948	AB641836	AB731663	AB731667
AB731669	AF004394	AF042100	AF042101	AF069140	AF286365	AF538302	AF538305
AF538306	AF538307	AJ271445	AY352275	AY835749	AY835753	AY835758	AY835761
AY835763	AY835768	AY835769	AY835773	AY835774	AY835775	AY835777	AY835779
AY835781	AY839827	D10112	DQ007903	DQ295192	DQ358809	DQ837381	DQ854716
DQ990880	EF363123	EF363124	EF363126	EF363127	GU177863	GU733713	JN397362
JN692465	JN692473	JN692475	JN944897	JN944911	JN944917	JN944930	JQ316126
JQ316127	JQ316128	JQ316130	JQ316131	JQ316132	JQ316133	JQ316134	JQ316135
JQ341411	JQ429433	JX500708	JX500709	K02007	K03455	KF384798	KF384799
KF384800	KF384801	KF384802	KF384803	KF384804	KF384805	KF384806	KF384807
KF384808	KF384810	KF384811	KF384812	KF384813	KF384814	KF561442	KF990605
KF990608	KJ140250	KJ140251	KJ140255	KJ140261	KJ140263	KJ140264	KJ140265
KJ140266	KT284371	L02317	M17449	M17451	M26727	M38431	M93258
U21135	U23487	U34604	U39362	U43096	U69591		
