# Supplementary material for: A scale-free analysis of the HIV-1 genome demonstrates multiple conserved regions of structural and functional importance
Source: PLoS Comput Biol. 2019 Sep 23;15(9):e1007345. doi: 10.1371/journal.pcbi.1007345 (PMC6791557; doi:10.1371/journal.pcbi.1007345)
Supplement: S23 Table — A number of the “complete genome” sequences in fact have a sequenced region that terminates upstream of the end of the region given by HXB2 nucleotide reference 9052–9093. These sequences have therefore not been used in the production of S27 Fig. The GenBank accession numbers of these sequences are given above. One of these sequences (accession number AF042104) does have sequence data downstream of the end of the region of interest, but there is a large piece of sequence data missing within the region itself, and so the entire sequence is omitted. (PDF) [file pcbi.1007345.s054.pdf]

AF042104	FJ388890	FJ388895	FJ388898	FJ388899	FJ388904	FJ388905	FJ388911
FJ388914	FJ388915	FJ388916	FJ388919	FJ388924	FJ388927	FJ388930	FJ388931
FJ388933	FJ388934	FJ388935	FJ388936	FJ388940	FJ388947	FJ388955	FJ388957
FJ388958	FJ388960	FJ388962	FJ388963	FJ388964	FJ388965	JF683765	JF683797
JF683804	JF683805	JF683807	JQ403097	JQ403098	KJ849807	KJ948656	KJ948660
M38431							
